# Cytosine base editors with minimized unguided DNA and RNA off-target events and high on-target activity

**DOI:** 10.1038/s41467-020-15887-5

**Published:** 2020-04-28

**Authors:** Yi Yu, Thomas C. Leete, David A. Born, Lauren Young, Luis A. Barrera, Seung-Joo Lee, Holly A. Rees, Giuseppe Ciaramella, Nicole M. Gaudelli

**Affiliations:** Beam Therapeutics, Cambridge, MA USA

**Keywords:** Genomics, CRISPR-Cas systems

## Abstract

Cytosine base editors (CBEs) enable efficient, programmable reversion of T•A to C•G point mutations in the human genome. Recently, cytosine base editors with rAPOBEC1 were reported to induce unguided cytosine deamination in genomic DNA and cellular RNA. Here we report eight next-generation CBEs (BE4 with either RrA3F [wt, F130L], AmAPOBEC1, SsAPOBEC3B [wt, R54Q], or PpAPOBEC1 [wt, H122A, R33A]) that display comparable DNA on-target editing frequencies, whilst eliciting a 12- to 69-fold reduction in C-to-U edits in the transcriptome, and up to a 45-fold overall reduction in unguided off-target DNA deamination relative to BE4 containing rAPOBEC1. Further, no enrichment of genome-wide C•G to T•A edits are observed in mammalian cells following transfection of mRNA encoding five of these next-generation editors. Taken together, these next-generation CBEs represent a collection of base editing tools for applications in which minimized off-target and high on-target activity are required.

## Abstract (web summary)

Cytosine base editors have been reported to induce off-target mutations in DNA and RNA. Here the authors identify next-generation CBEs with reduced guide-independent off-target editing profiles and retain high on-target editing activity.

## Introduction

Base editors are gene editing tools that enable efficient and programmable correction of point mutations for both research and therapeutic applications^1,2^. Unlike CRISPR-associated nuclease gene editing approaches, base editors do not create double-stranded DNA breaks and therefore minimize the formation of undesired editing byproducts, including insertions, deletions, translocations, and other large-scale chromosomal rearrangements^1–4^. Cytosine base editors (CBEs) are comprised of a cytidine deaminase fused to an impaired form of Cas9 (D10A nickase) tethered to one (BE3) or two (BE4) monomers of uracil glycosylase inhibitor (UGI)^5^. This architecture of CBEs enables the conversion of C•G base pairs to T•A base pair in human genomic DNA, through the formation of a uracil intermediate^1^.

Although CBEs lead to robust on-target DNA base editing efficiency in a variety of contexts (e.g., rice, wheat, human cells, and bacteria, reviewed here^3^), recent reports have demonstrated that treatment of cells with high doses of BE3 can lead to low, but detectable cytosine deamination in both DNA^6–8^ and cellular RNA^9^ in a guide-independent fashion. Specifically, Zuo et al. observed substantial off-target single-nucleotide variants (SNVs) in mouse embryos upon treatment with BE3 containing rAPOBEC1^7^. A mutation frequency of one in ten million bases was detected, resulting in ~300 additional SNVs compared with untreated cells^4^. Even though this mutation frequency is within the range of somatic mutations that occurs naturally in mouse and human cells^10,11^, given the therapeutic importance of CBEs, we were motivated to develop next-generation CBEs that function efficiently at their on-target loci with minimal off-target edits relative to the foundational base editors, BE3/4, which contain rAPOBEC1.

To mitigate guide-independent off-target editing events, we a develop sensitive, high-throughput cellular assay to select next-generation CBEs that display reduced guide-independent off-target editing profiles relative to rAPOBEC1-based CBEs, whilst maintaining equivalent or superior on-target editing frequencies. We screen 153 cytidine deaminases with diverse sequences within the context of a base editor and identify four CBEs with the most promising on/off-target editing profile. These next-generation CBEs (BE4 with either RrA3F, AmAPOBEC1, SsAPOBEC3B, or PpAPOBEC1) are further optimized for superior on- and off-target DNA editing profiles through structure-guided mutagenesis of the deaminase domain. The resulting next-generation CBEs (BE4 with either RrA3F [wt, F130L], AmAPOBEC1, SsAPOBEC3B [wt, R54Q], or PpAPOBEC1 [wt, H122A, R33A]) display high DNA on-target editing activity and minimized unguided DNA and RNA off-target activity.

## Results

### *Incis*/*in trans*-assay for base editors

Since both the reported DNA^6,7^ and RNA^9^ off-target deamination events result from unguided, Cas9-independent deamination events, we hypothesize that these undesired byproducts are caused by the intrinsic ssDNA/ssRNA binding affinities of the cytidine deaminase itself. The canonical CBE base editor BE3, used in the aforementioned genome-wide deamination reports^6,7,9^, contains an N-terminal cytidine deaminase rAPOBEC1, an enzyme that has been extensively characterized to deaminate both DNA^12,13^ and RNA^10,14^ when expressed in mammalian, avian, and bacterial cells. Indeed, CBEs containing rAPOBEC1 (BE3^1^, BE4^5^, and BE4max^15^) are widely utilized base editing tools due to their overall high on-target DNA editing efficiencies. Thus far, it has not been extensively evaluated whether existing, and/or engineered deaminases might provide similarly high on-target DNA editing efficiency whilst preserving a minimized unguided, deaminase dependent, off-target editing profile.

**Fig. 1:**
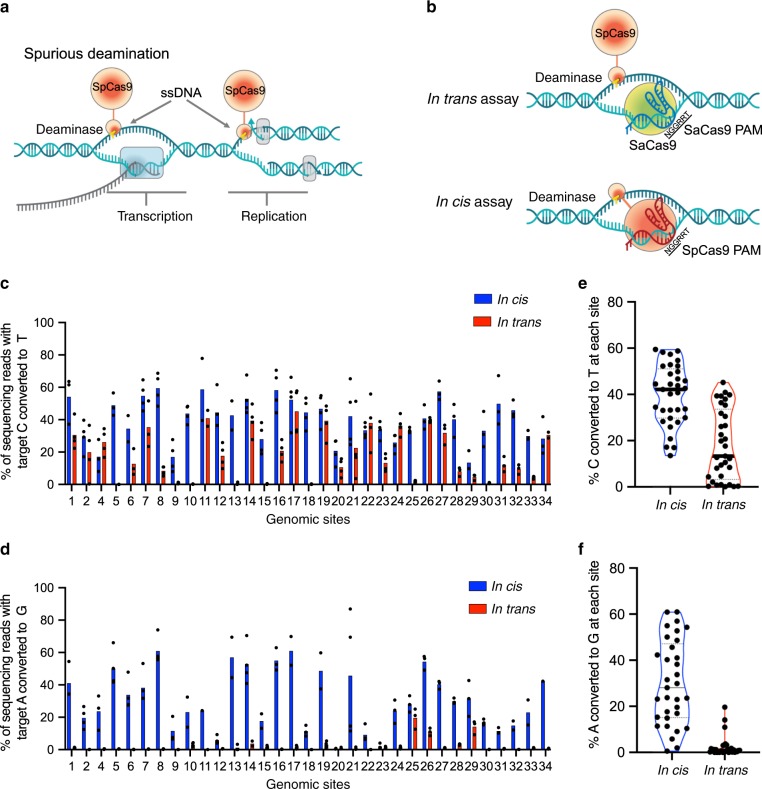
Unguided ssDNA deamination and *in cis/in trans*-assay. **a** potential ssDNA formation in the genome during transcription or replication. **b** experimental design of *in cis/in trans*-assay. Separate constructs encoding SaCas9, gRNA for SaCas9 and base editor were used to transfect HEK293T cells. *In cis*- and *in trans*-activity were measured in different transfections at the target site with NGGRRT PAM sequence. *In cis/in trans*-activities of BE4 with rAPOBEC1 (**c, e**) or ABE7.10 (**d, f**) at 33 genomic sites (site 1, 2, 4–34). Blue bars indicate *in cis*, on-target editing, red bars indicate *in trans*-editing; base editing efficiencies were reported for the most edited base in the target sites. Individual data points are shown for *n* = 4 independent biological replicates, expect for conditions with data points not included due to insufficient reads (<5000). The bar for each condition represents the mean value. Each dot in the violin plots represents the mean editing efficiency at one target site shown in the bar graphs. All data presented are provided as Source Data.

In order to screen a wide range of next-generation CBE candidates for preferred on- and off-target editing profiles, we established a high-throughput assay to evaluate unguided ssDNA deamination. Since genome-wide deamination has been reported to occur most frequently in highly transcribed regions of the genome^6,7^, it is tempting to hypothesize that rAPOBEC1 can preferentially access transiently available ssDNA that is generated during DNA replication or transcription (Fig. 1a). Therefore, we sought to mimic the availability of genomic ssDNA by presenting this substrate via a secondary R-loop generated by an orthogonal SaCas9/sgRNA complex and quantifying the amount of unguided editing on this ssDNA substrate with fully intact CBEs (Fig. 1b). In this *in cis*/*in trans*-assay, the same target site followed by “NGGRRT” sequence was used for testing *in trans*-activity and *in cis*-activity in separate transfections. Here, “*in cis*” activity refers to on-target DNA base editing (directed by SpCas9 nickase that recognizes the NGG PAM) and “*in trans*” activity refers to base editing in the secondary SaCas9-induced R-loop (SaCas9 recognizes the NNGRRT PAM), to which the base editor is not directed by its own sgRNA, mimicking the transient, unguided off-targeting editing events in the genome observed in mouse embryos^7^ and rice^6^. Doman et al. used a similar assay to examine guide-independent deamination, with the distinction that *in cis*- and *in trans*-activities of CBEs were measured in the same transfection but at different target sites^8^.

Crucially, we assessed the validity and sensitivity of our on- and off-target editing evaluation assay using BE4- and ABE7.10-treated cells. Previously, it has been shown that cells treated with BE3 (CBE with rAPOBEC1), but not ABE7.10, display an increase in guide-independent, genome-wide transition mutations^6,7^. Consistent with these findings, our assay also showed that cells treated with BE4 (with rAPOBEC1) led to much greater levels of *in trans*-editing than ABE7.10 (Fig. 1c–f). The sensitivity of the assay is demonstrated by the result that treatment of cells with BE4 led to >5% C-to-T editing at 24 of 33 loci tested *in trans*, up to a maximum of 57.8% (Fig. 1c, e). The mean *in cis/in trans*-activity across 33 target sites is 39.8%/18.2% for BE4 (Fig. 1c, e) and 30.3%/2.2% for ABE7.10 (Fig. 1d, f). We speculate that the sensitivity of our assay may be attributed to both the presentation of the ssDNA substrate via a stable R-loop generated by catalytically impaired SaCas9 nickase with two UGI attached (SaCas9(D10A)-2XUGI) and the fact that the deamination events are measured by Illumina amplicon sequencing with at least 5,000 mapped reads per sample.

### Mutagenesis of rAPOBEC1 in BE4

First, we used this cellular assay to test if mutagenesis of deaminases is an effective strategy toward reducing guide-independent DNA off-target activity. Utilizing a homology model of rAPOBEC1 aligned with the crystal structure of hA3C^16,17^ (Supplementary Fig. 1), we chose 15 residues that may be involved in ssDNA binding and 8 that likely affect catalytic activity. Through mutagenesis of these 23 residues, preferentially into alanine, we identified seven high-fidelity (HiFi) mutations in rAPOBEC1 (R33A, W90F, K34A, R52A, H122A, H121A, and Y120F) that greatly reduce *in trans*-activity without dramatically reducing *in cis*-activity (Supplementary Fig. 2). Further, we identify two mutations (T36A and R126A) that moderately reduce *in trans*-DNA off-target activity (Supplementary Fig. 2). Previously described rAPOBEC1 mutations in BE4 constructs are reported to improve its RNA off-target editing profile (e.g., “SECURE” CBEs containing R33A or [R33A, K34A] mutations)^9^, or alter the editing window (e.g., BE4-YE1 containing W90Y and R126E substitutions)^18^ and have recently been identified to reduce genome-wide unguided C-to-T editing^8^. However, at ten loci we evaluated, SECURE-CBE (R33A) and SECURE-CBE (R33A, K34A) retained only ~53% and ~18% on-target activity, respectively, as compared with BE4 with rAPOBEC1 (Supplementary Fig. 3).

Among the seven HiFi mutants, BE4-rAPOBEC1-H122A was the only CBE that showed equivalent *in cis*-activity compared with BE4-rAPOBEC1 at ten target sites tested (the remaining HiFi mutants yielded a range of 18–71% *in cis*-activity as compared with BE4). Of note, the average *in cis/in trans*-editing efficiency is 32.3%/11.8% for BE4-rAPOBEC1 and 34.5%/4.2% for BE4-rAPOBEC1-H122A (Supplementary Fig. 3). This mutagenic study, in which we included the SECURE-CBEs^9^, indicated that BE4-rAPOBEC1-H122A was the most promising CBE among the group tested with respect to its relative *in cis/in trans*-editing profiles.

### Cytidine deaminase screening round 1

**Fig. 2:**
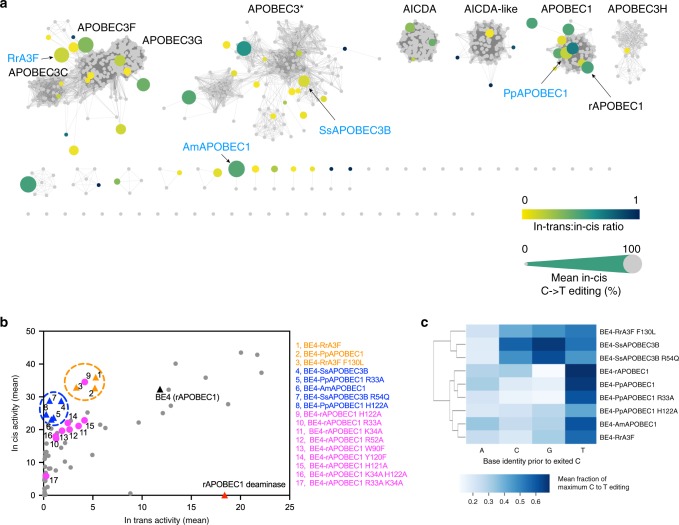
Deaminase similarity network and next-generation CBEs with high *in cis*-activities and reduced *in trans*-activities. **a** similarity network of APOBEC-like deaminases. This network was constructed using methods described in Supplementary Note 1. The size of the dots represents the average *in cis*-activity; the color of the dots represents the average *in trans/in cis*-ratio. Mean *in cis*/*in trans*-activities were calculated based on data from *n* = 3 independent biological replicates on-target sites 1, 4, 6, and the individual editing efficiencies are displayed in Supplementary Figs. 4, 7, and 8. **b** *In cis*-activity vs. *in trans*-activity of CBEs tested. The three orange dots represent next-generation CBEs with equivalent *in cis*-activity to BE4 with rAPOBEC1; the five blue dots represent next-generation CBEs with minimized *in trans*-activity; the nine pink dots represent BE4- rAPOBEC1 mutants; gray dots represent other CBEs screened. Base editing efficiencies were reported for the most edited base in the target sites. The mean activities were calculated based on data from of *n* = 4 independent biological replicates (orange and blue dots), *n* = 3 independent biological replicates (pink and gray dots) on-target sites 1, 4, and 6. **c** mean *in cis*-activity at cytosines prior to different bases at target sites 1–10. Values reflect the mean of *n* = 4 independent biological replicates. All data presented are provided as Source Data.

Next, we began a broader search for next-generation CBEs with a preliminary screen of CBEs containing cytidine deaminases from well-characterized families including APOBEC1, APOBEC2, APOBEC3, APOBEC4, AID, CDA, etc. Among these deaminases, four APOBEC1s (MdAPOBEC1, PpAPOBEC1, OcAPOBEC1, and hAPOBEC1) showed a high *in cis/in trans*-ratio at select sites (Fig. 2a and Supplementary Figs. 4 and 5). A CBE containing PpAPOBEC1 deaminase (67% sequence identity to rAPOBEC1) showed comparable on-target DNA activity to BE4 with rAPOBEC1 and on average 2.3-fold decrease in *in trans*-activity across ten sites tested (Supplementary Fig. 6). HiFi mutations of PpAPOBEC1 were predicted based on sequence alignment (Supplementary Fig. 5), and BE4 with PpAPOBEC1 containing either H122A or R33A mutations displayed desirable editing profiles (Supplementary Fig. 6), with 76% and 73% average *in cis*-activities and 45- and 12-fold reduction in average *in trans*-activities as compared with BE4 with rAPOBEC1, respectively. Together, these data established BE4 with PpAPOBEC1 as the preferred CBE from our first round of screening.

### Cytidine deaminase screening round 2

Encouraged by these results, a second round of screening of 43 APOBEC-like cytidine deaminases with broad sequence diversity was performed (Fig. 2a, b, Supplementary Fig. 7, and Supplementary Note 1). We performed a protein BLAST with hAPOBEC1 as the query sequence to generate a sequence similarity network with the top 1000 sequences, enabling us to select cytidine deaminases with broad sequence diversity. From this screening campaign, three constructs (BE4s with RrA3F, AmAPOBEC1, or SsAPOBEC3B) showed robust on-target DNA editing activities that are comparable with BE4 (with rAPOBEC1) with 111%, 72%, and 89% average *in cis*-activities, respectively, and 2.2-, 14.8-, and 6.6-fold decrease on average *in trans*-activity (Fig. 2b and Supplementary Figs. 8–10). Notably, the sequence identity of these editors to rAPOBEC1 is 23%, 31%, 20%, respectively. BE4 constructs with either RrA3F or SsAPOBEC3B displayed comparably higher editing frequencies at GC target sites that are not well edited with BE4 (with rAPOBEC1) (Fig. 2c). In addition, we observed variations in editing windows of *in cis*- and *in trans*-editing with these editors (Supplementary Fig. 9). Finally, we expanded our screen again to interrogate a set of 80 putative cytidine deaminases from other protein families that has less sequence homology to APOBECs. None of these deaminases showed >1% editing efficiency in the context of BE4 at the site tested (Supplementary Fig. 11).

### Mutagenesis of next-generation CBEs

We further engineered our BE4 editors (containing RrA3F, AmAPOBEC1, or SsAPOBEC3B) via rational mutagenesis for optimal on- and off-target editing outcomes (Supplementary Figs. 2 and 3**)**. We installed select HiFi mutations from our rAPOBEC1 studies into these BE4 editors based on homology modeling of existing crystal structures of similar proteins (Supplementary Fig. 12). Two engineered CBEs containing RrA3F F130L and SsAPOBEC3B R54Q, emerged from our high-throughput screen that demonstrated improved on- to off-target editing profiles (Fig. 2b and Supplementary Fig. 8), with 102% and 89% average *in cis*-activities and 3.6- and 19.6-fold decrease in average *in trans*-activities, respectively, relative to BE4 with rAPOBEC1. These two next-generation CBEs with optimal *in cis*/*in trans*-editing profiles were used in further studies. 

The eight next-generation editors [BE4 with PpAPOBEC1 (wt, H122A, or R33A), RrA3F (wt), AmAPOBEC1 (wt), and SsAPOBEC3B (wt)] can be divided into two groups based on their *in cis/in trans*-activity (Fig. 2b): (1) contains three CBEs with high on-target editing efficiency (101% to 111% *in cis*-activity relative to BE4 containing rAPOBEC1) and reduced *in trans*-activity (2.2- to 3.6-fold compared with BE4 with rAPOBEC1), and (2) contains five CBEs with slightly reduced on-target editing efficiency (71 to 89% *in cis*-activity relative to BE4 containing rAPOBEC1) and minimized *in trans*-activity (6.6- to 45-fold decrease compared with BE4 with rAPOBEC1). Since the majority of rAPOBEC1-BE4 variants tested resulted in an inferior on- to off-target editing ratio, as compared with BE4 constructs containing alternative cytidine deaminase (Fig. 2b), we advanced our work by refining and characterizing BE4 constructs containing non-rAPOBEC1 cytidine deaminases in order to best optimize CBEs for high on-target gene editing activity and minimized off-target, guide-independent deamination.

### Evaluation of guided DNA off-target editing

With these next-generation CBEs in hand, we selected a subset [BE4 with PpAPOBEC1 (wt, H122A or R33A), RrA3F (wt), AmAPOBEC1 (wt), SsAPOBEC3B (wt)] to further characterize their off-target editing activity. We began by evaluating guide-dependent DNA off-target editing at known Cas9 off-target loci^19^ associated with three SpCas9 sgRNAs. Because base editing at Cas9-guided off-target sites relies on the interplay between the inherent properties of a given deaminase and Cas9 binding at the mismatched loci, exchanging the deaminase within base editor architecture may lead to different editing outcomes^18^. Guide-dependent off-target activities of BE4 with PpAPOBEC1 were similar to BE4 with rAPOBEC1 (Supplementary Fig. 13). Interestingly, some next-generation CBEs showed reduced guide-dependent off-target editing for at least one sgRNA tested, and our HiFi mutations also reduced guide-dependent off-target editing efficiency (Supplementary Fig. 13). For example, at three of the most highly edited off-target sites (HEK2, site 1; HEK3, site3; and HEK4, site 1), cells treated with BE4- containing AmAPOBEC1 demonstrated 19-, 27-, and 3.3-fold reduction in guide-dependent off-target editing than BE4 with rAPOBEC1 respectively (Supplementary Fig. 13). Notably, BE4 with PpAPOBEC1 H122A showed greater than threefold reduction in guide-dependent off-target editing than BE4 with PpAPOBEC1 at these three sites, while no observable decrease was found in on-target editing (Supplementary Fig. 13). These data indicate that next-generation CBEs can yield more favorable or equivalent guided off-target editing profiles as compared with BE4 containing rAPOBEC1. Furthermore, to validate that base editing outcomes resultant from our next-generation CBES were not due to differences in editor expression, we quantified the amount of protein produced from cells transfected with our next-generation CBEs and BE4 (rAPOBEC1) and show that next-generation CBE protein levels are comparable with amounts observed for BE4 with rAPOBEC1 (See Supplementary Note 2 and Supplementary Fig. 14).

### Evaluation of unguided RNA and DNA off-target editing

**Fig. 3:**
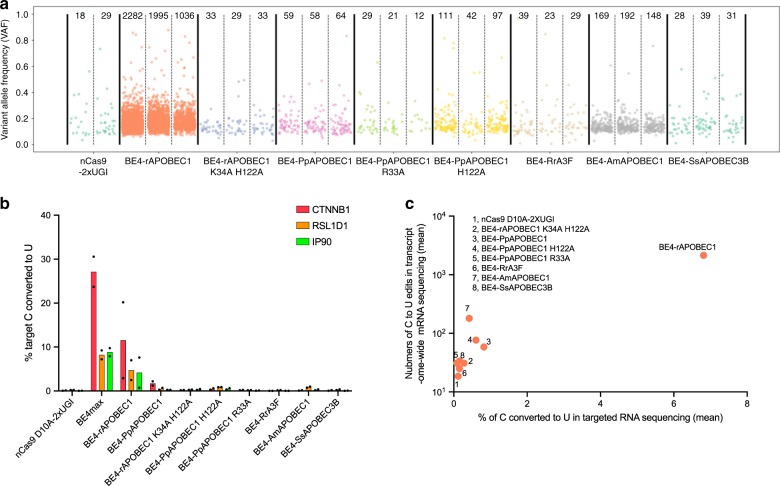
Next-generation CBEs with reduced RNA off-target editing efficiency relative to BE4 in mammalian cells. Transcriptome-wide mRNA sequencing (**a**) and targeted RNA sequencing (**b**) of HEK293T cells expressing next-generation CBEs. Base editing efficiencies were reported for the most edited base in the target sites. Values in **b** reflect the mean of *n* = 2 independent biological replicates. **c** correlation of the numbers of C-to-U mutations observed in transcriptome-wide mRNA sequencing with percentage of C-to-U conversion in targeted RNA sequencing. Values for *x*-axis reflect the mean editing efficiency at sites shown in **b** from *n* = 2 independent biological replicates. Values for *y*-axis reflect the mean of *n* = 3 biological replicates shown in **a**. All data presented are provided as Source Data.

We further characterize off-target RNA editing activity of selected next-generation CBEs. Plasmid-based overexpression of BE3 containing rAPOBEC1 has previously been shown to induce transcriptome-wide RNA cytosine deamination, and as such we evaluated our next-generation CBEs in similar conditions^9,20^. Satisfyingly, six next-generation BE4s tested showed 12 to 69-fold reduction in C-to-U edits as compared with BE4 with rAPOBEC1 (Fig. 3a). Notably, treatment of cells with BE4s containing RrA3F, SsAPOBEC3B, and PpAPOBEC1 R33A led to frequencies of C-to-U edits that are comparable with cells treated with nCas9 (D10A)–2×(UGI). In addition, deep-sequencing analysis of selected regions in the transcriptome reveal C-to-U editing outcomes that were consistent with transcriptome-wide mRNA sequencing data (Fig. 3b, c). Taken together, these data suggest that next-generation CBEs result in reduced transcriptome-wide RNA editing compared with BE3 or BE4 containing rAPOBEC1.

Next, we investigated if our next-generation CBEs can reduce guide-independent off-target editing in the genome. Whole genome sequencing (WGS) experiments were performed on base-editor-treated HEK293T cells grown by clonally expanding single cells (Supplementary Fig. 15a)^21^. We compared the frequency of CBE-induced mutations in cells where base editors were delivered as either plasmid or mRNA. Four to five biological replicates (independent clonal expansions) for each treatment group were sequenced.

**Fig. 4:**
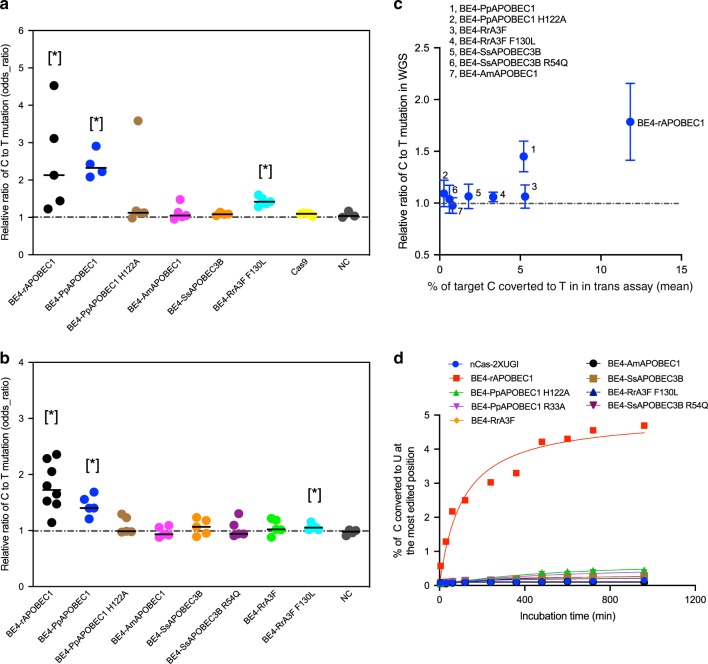
Next-generation CBEs with reduced DNA off-target editing efficiency relative to BE4 in HEK293T cells. Relative ratio of C-to-T mutation (odds ratio) of single cell expansions treated with base editor plasmid (**a**) and mRNA (**b**). Each dot represents one WGS sample from different single cell expansions. The odds ratios quantify the fold change in mutation rates for the editor-induced mutation type (C->T), with an odds ratio of 1 implying no change from untreated cells. The black line represents the median of *n* = 3, 4, 5, and 8 biological replicates, as can be determined for each sample by the number of included data in the graph. *P* values were calculated using one-sided Mann Whitney *U* test between the test group and the untreated group, and *P value < 0.05. *P* value: **a** BE4-rAPOBEC1, 0.018; BE4-PpAPOBEC1, 0.026; BE4-BE4-RrA3F F130L, 0.018; **b** BE4-rAPOBEC1, 0.004; BE4-PpAPOBEC1, 0.010; and BE4-RrA3F F130L, 0.010. **c** correlation between relative ratio of C-to-T mutation in each WGS sample treated with base editor mRNA and percentage of C-to-T conversion in *in trans*-assay shown in Supplementary Fig. 8. The values and error bars for *y*-axis reflect the mean and s.d. of *n* = 3, 4, 5, and 8 biological replicates, as can be determined for each sample by the number of included data in the **b**. Value for *x*-axis reflect the mean editing efficiency at targets 1–10 from *n* = 4 independent biological replicates. **d** C-to-U editing efficiency of selected CBEs on DNA oligo 1 in in vitro enzymatic assay. Sequence of the oligo is listed in Supplementary Table 3. Values and error bars reflect the mean and s.d of *n* = 2 independent biological replicates from *y* = 2 experiments. All data presented are provided as Source Data.

Across cells treated with plasmids encoding CBEs, we found no statistically significant increase in the ratio of C-to-T mutations following treatment with non-rAPOBEC1 containing CBEs (BE4-PpAPOBEC1 H122A, BE4-AmAPOBEC1, or BE4-SsAPOBEC3B) (Fig. 4a). However, we identified a statistically significant increase in the ratio of C-to-T mutations following cellular treatment with BE4-rAPOBEC1 (as previously reported^6–8,21^), BE4-PpAPOBEC1, and BE4-RrA3F F130L compared with untreated controls (*P* value = 0.018, 0.026, and 0.018, respectively, one-sided Wilcoxon–Mann–Whitney *U* test) (Fig. 4a). For mRNA delivery, only samples treated with BE4-rAPOBEC1, BE4-PpAPOBEC1, and BE4-RrA3F F130L showed a significant increase in the ratio of C-to-T mutations compared with untreated controls (*P* value = 0.004, 0.010, and 0.010, respectively, one-sided Wilcoxon–Mann–Whitney *U* test) (Fig. 4b). Notably, although cells treated with BE4-RrA3F F130L showed a significant increase in the ratio of C-to-T mutations, the absolute value of the increase is very small (the mean odds ratio is 1.06, compared with untreated control of 0.97). Across all editors, a lower level of C-to-T mutations was detected in cells treated with mRNA as compared with plasmid delivery (*P* value = 0.0074, one-sided Wilcoxon–Mann–Whitney *U* test). Notably, the reduction in genome-wide cytosine deamination with mRNA delivery was not a result of decrease in on-target editing efficiency: a higher mean on-target editing was observed with mRNA delivery compared with plasmid delivery (Supplementary Fig. 15b, c). These results showed that combining mRNA delivery and the use of next-generation CBEs is a highly effective strategy to eliminate or decrease detectable genome-wide DNA off-target editing activity while maintaining or increasing on-target editing.

Our results suggest that replacing rAPOBEC1 in canonical BE3 and BE4 with next-generation deaminases, such as BE4-PpAPOBEC1 H122A, BE4-AmAPOBEC1, BE4-SsAPOBEC3B (wt, R54Q), and BE4-RrA3F (wt, F130L), leads to a more favorable on-target vs. off-target editing profile. We also demonstrated that there is a relationship between C-to-T mutations detected from WGS and *in trans*-activity (Fig. 4c). As others have found^8^, we also demonstrate here that our cost-effective, high-throughput *in trans*-activity assay can be used as a valid and sensitive method to evaluate the unguided ssDNA deamination activity of base editors.

Furthermore, we evaluated the unguided deamination activity of CBEs in an in vitro assay comprised of base editor protein and synthetic ssDNA substrate (Fig. 4d and Supplementary Fig. 16). From this assay we observed 7.5- to 12-fold more C-to-U modified ssDNA at 5 min and 10- to 50-fold more product formed at 16 h by BE4 with rAPOBEC1 compared with our next-generation CBEs (Fig. 4d). Together with our *in cis/in trans*-assay and WGS experiments, these data further validate that our next-generation CBEs display reduced activity on exposed ssDNA, a feature that is especially important for both research and therapeutic applications.

### On-target activity comparison with published CBEs

**Fig. 5:**
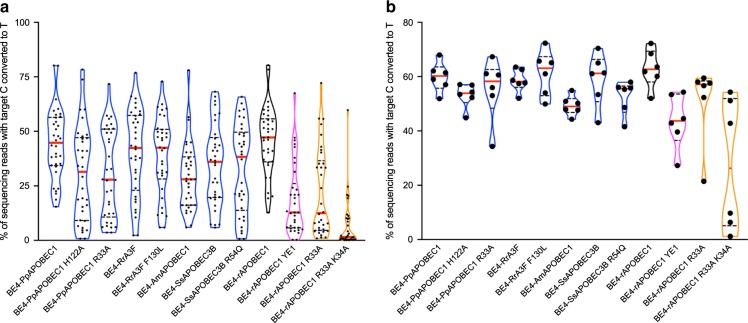
Next-generation CBEs showed higher DNA on-target editing efficiencies than SECURE-CBEs and BE4-YE1. C-to-T editing efficiencies of next-generation CBEs, SECURE-BE4s, and BE4-YE1 at 33 genomic sites (site 1–22 and 24–34) (**a**) and 6 previously published sites (**b**). Base editing efficiencies are reported for the base within the target site with the highest editing frequency. Next-generation CBEs reported here are in blue, BE4 (containing rAPOBEC1)^5^ is in black, BE4-YE^19^ is in pink, and SECURE-CBEs^9^ are in orange. The red horizontal line across each plot represent the mean of *n* = 3 independent biological replicates, expect for conditions with data points not included due to insufficient reads (<5000). All data presented are provided as Source Data.

Recently, engineered CBEs displaying minimized unguided C-to-U editing events in the transcriptome (“SECURE” CBEs, with R33A or [R33A, K34A] substitutions)^9,20^, or minimized genome-wide unguided cytosine deamination (BE4-YE1, with W90Y and R126E substitutions)^18^ were reported. Since these recently published CBEs, as well as the CBEs reported here, all result in highly minimized unguided off-target deamination events, we were motivated to compare their relative on-target editing activities on various genomic sites. When our eight next-generation CBEs, SECURE-BE4, and BE4-YE1 were tested for on-target editing at the 33 sequence-diverse loci used in this study, we observed that our next-generation CBEs enabled higher mean editing efficiencies compared with SECURE-BE4 and BE4-YE1 (Fig. 5). Next-generation CBEs showed 64.0–96.8% relative mean on-target editing activity to BE4-rAPOBEC1 (Fig. 5a). In contrast, the relative mean on-target editing activities of BE4-rAPOBEC1 YE1, BE4-rAPOBEC1 R33A, and BE4- rAPOBEC1 R33A K34A are 41.1%, 49.0%, and 15.4%, respectively (Fig. 5a). When the collection of off-target minimized CBEs were tested on six previously published sites (FANCF, RNF2, EMX1, HEK2, HEK3, and HEK4)^8^, our next-generation CBEs showed 78.2–97.3% mean on-target editing activity as compared with BE4-rAPOBEC1, while the relative mean on-target editing activities of BE4-rAPOBEC1 YE1, BE4- rAPOBEC1 R33A, and BE4- rAPOBEC1 R33A K34A are 69.3%, 80.1%, and 43.8%, respectively (Fig. 5b). BE4-rAPOBEC1 showed high (>50%) on-target activity at these six previously published sites^5,8^ compared with more varied editing outcomes from the 33 guides we tested in this study (<5% to >80% editing efficiency) (Fig 5).

Since base editing outcomes are site- and context-dependent^1,5,18^, identification of the most generalizable tools can best be achieved through survey of a large number of sequence-diverse sites that display variable on-target editing frequencies when targeted with the current standard set of BEs. Comparisons between genome editing tools where only a few sites are interrogated may be misleading for those who hope to perform efficient base editing at novel loci. Our comparison highlights that, across a diverse range of target sites, the eight off-target minimized, next-generation CBEs reported in this work enable higher on-target editing efficiencies than previously published CBEs with minimized DNA/RNA off-target editing profiles (Fig. 5).

## Discussion

Through an extensive and systematic screening campaign, we discovered and subsequently characterized eight next-generation CBEs. We developed high-throughput assays to evaluate unguided ssDNA editing efficiency and from a total of 153 deaminases screened, four enzymes (PpAPOBEC1, RrA3F, AmAPOBEC1, and SsAPOBEC3B) were identified to have reduced off-target editing whilst maintaining equivalent or superior on-target editing. Together with structure-guided mutagenesis on these four, non-rAPOBEC1 containing, constructs we highlight eight next-generation CBEs (BE4-PpAPOBEC1 [wt, H122A, R33A], BE4-RrA3F [wt, F130L], BE4-AmAPOBEC1, and BE4-SsAPOBEC3B[wt, R52Q]) with reduced to minimized off-target editing efficiency and comparable on-target editing efficiency to BE4 containing rAPOBEC1. Transcriptome-wide RNA deamination associated with expression of a subset of our next-generation CBEs (BE4-RrA3F, BE4-SsAPOBEC3B, and BE4-PpAPOBEC1 R33A) was comparable with that of nCas9(D10A)-2xUGI, whilst the average on-target editing was about 3.9- to 5.7-fold higher than the previously published SECURE-CBE (BE4 with rAPOBEC1 [R33A,K34A])^9^.

Most importantly, no significant enrichment of genome-wide, guide-independent C-to-T edits was observed in cells transfected with mRNA encoding either BE4-RrA3F(wt, F130L), BE4-AmAPOBEC1, or BE4-SsAPOBEC3B (wt, R54Q). Since the most efficient next-generation CBE can be site- and application-dependent, our strategy to explore the evolutionary diverse families of deaminases has enabled a significantly expanded tool-box of CBE editors that can be used to successfully edit the genome at therapeutically relevant sites.

## Methods

### General methods

Constructs used in this study were obtained by USER assembly, Gibson assembly, or synthesized by Genscript. Gene fragments used for PCR were purchased as mammalian codon-optimized gene fragments from IDT, Thermo Fisher Scientific and Twist Bioscience. PCR were performed with primers ordered from IDT using either Phusion U DNA Polymerase Green MultiPlex PCR Master Mix (Thermo Fisher) or Q5 Hot Start HiFi 2x Master Mix (New England Biolabs). Endo-free plasmids used for mammalian transfection were prepped using ZymoPURE II Plasmid Midiprep (Zymo Research Corporation) from 50 mL Mach1 (Thermo Fisher) culture. mRNA was synthesized by IVT reactions, and gRNA with standard modification was purchased from Axolabs. Sequences for CBEs, protospacer sequences for sgRNA, and oligos used in this study can be found in Supplementary Table 1–4. Prism8 (v 8.3.0), Excel (v16.32), R (v3.4.3) were used for data analysis in addition to data analysis listed in Supplementary Note 1 and 3–5.

### HEK293T cell culture

HEK293T cells [CLBTx013, American Type Cell Culture Collection (ATCC)] were cultured in Dulbecco’s modified Eagles medium plus Glutamax (10566-016, Thermo Fisher Scientific) with 10% (v/v) fetal bovine serum (A31606-02, Thermo Fisher Scientific) following culture method on ATCC website. Cell culture incubator was set to 37 °C with 5% CO_2_. Cells were tested negative for mycoplasma after receipt from supplier.

### Transfection conditions and harvest of cells

HEK293T cells were seeded onto 96- or 48-well poly-d-lysine treated BioCoat plates (Corning) at a density of 12,000 cells/well (96-well) or 35,000 cells/well (48-well). Transfection of HEK293T cells were done after 18–24 h. To each well of cells in 96-well (48-well) plate, 90 ng (300 ng) base editor or control plasmid, 30 ng (100 ng) sgRNA plasmid and 1 μL (1.5 μL) Lipofectamine 2000 (Thermo Fisher Scientific) were added following the manufacturer’s instructions. For *in trans*-editing experiments, 60 ng (180 ng) nSaCas9 (D10A)-2xUGI plasmid were added to the transfection mixture.

After ~64 h of incubation, cells were harvested. For NGS amplicon sequencing, media were aspirated and 50–100 μL QuickExtract™ DNA Extraction Solution (Lucigen) were added to each well. gDNA extraction was performed according to the manufacturer’s instructions; For RNA extraction (48-well plate), 300 μL RTL plus buffer (RNasy Plus 96 kit, Qiagen) was added to each well. RIPA buffer (100 μL per well, Thermo Fisher Scientific) was used to lysis the cells for protein quantification purpose. For in vitro enzymatic assays (48-well plate), each well of cells were lysed with 100 μL M-per buffer (Thermo Fisher Scientific).

### Next-generation sequencing for DNA editing

Genomic DNA samples were extracted using QuickExtract DNA extract solution (Lucigen) following the manufacturer’s instructions. Overall, 2 μL of gDNA was added to a 25 μL PCR reaction containing Phusion U Green Multiplex PCR Master Mix and 0.5 μM of each forward and reverse primer. Following amplification, PCR products were barcoded using unique Illumina barcoding primer pairs. Barcoding reactions contained 0.5 μM of each Illumina forward and reverse primer, 1 μL of PCR mixture containing amplified genomic site of interest, and Q5 Hot Start HiFi 2x Master Mix in a total volume of 25 μL. PCR were carried out using thermocyclers with the following program: 95 °C for 3 min, 30 cycles (amplification)/7 cycles (barcoding) of 95 °C for 15 s, 62 °C for 20 s, and 72 °C for 20 s, and then 72 °C for 2 min. Primers used for site-specific mammalian cell genomic DNA amplification are listed in Supplementary Table 3.

NGS data were analyzed by performing four general steps: (1) Illumina demultiplexing, (2) read trimming and filtering, (3) alignment of all reads to the expected amplicon sequence, and (4) generation of alignment statistics and quantification of editing efficiencies. Each step is described in more detail in Supplementary Note 2.

### Transcriptome sequencing for RNA off-target editing

Total RNA extraction was done using the Rnasy Plus 96 kit (Qiagen) following the manufacturer’s protocol. An extra on-column Dnase I (Rnase-Free Dnase Set, Qiagen) digestion step was added before the washing step following the manufacturer’s instructions.

cDNA samples were generated from the isolated mRNA using SuperScript IV One-Step RT-PCR System (Thermo Fisher Scientific) according to the manufacturer’s instructions. NGS for targeted RNA sequencing was performed using the same protocol as for DNA editing. For whole transcriptome sequencing, mRNA isolation was performed from 100 ng total RNA was done using NEBNext Poly(A) mRNA Magnetic Isolation Module (NEB). Exome sequencing library preparation was performed using the NEBNext^®^ Ultra™ II Directional RNA Library Prep Kit for Illumina following manufacturer’s instructions. The optional second SPRI beads selection was performed to remove residue adaptor contamination. The libraries made were analyzed using fragment analyzer (Agilent) and sequencing was conducted at Novogene on NovaSeq S4 flow cell. Data analysis was performed as described in Supplementary Note 4.

### In vitro enzymatic assays

Cells were lysed in M-per buffer and concentration of Cas9 was performed using automated Ella assay using Ella instrument (Protein Simple). An aliquot of 5 μL cell lysate or Cas9 standard solution was mixed with 45 μL sample diluent (D-13) and the mixture was added to 48-digoxigenin cartridges. Cas9 in base editor complex were quantified using anti-Cas9 antibody (7A9-A3A, Novus Biologicals). The protein concentration was adjusted to 0.1 nM (final concentration) and mixed with 1 μL oligo (oligo sequence included in Supplementary Table 3) at 0.5 μM concentration in reaction buffer (20 mM Tris pH 7.5, 150 mM NaCl, 1 mM DTT, 10% glycerol) for indicated amount of time. The assay was quenched by heat inactivation at 95 °C for 3 min and the product formation was quantified using percentage of C-to-T conversion (NGS) and input amount of oligos.

### Whole genome sequencing for DNA off-target editing

HEK293T cells were seeded onto six-well cell-bind cell culture plates (Corning) at a density of 200,000 cells/well. Transfection of HEK293T cells were done after 18–24 h. For plasmid transfection, 1500 ng base editor or control plasmid, 500 ng sgRNA plasmid, and 10 μL Lipofectamine 2000 (Thermo Fisher Scientific) were used in each transfection; for mRNA transfection, 2000 ng base editor or Cas9 mRNA, 500 ng sgRNA guide and 7.5 μL Lipofectamine messengermax (Thermo Fisher Scientific) were used.

After 120 h (plasmid transfection) or 60 h (mRNA transfection) of incubation, the cells were stained using B2M antibody (Cell signaling). B2M knockout cells were sorted as single cells into 96-well plate. After 7 days, colonies expanded from single cells were treated by TrypLE and dispersed on the same plate. After 3–4 days, cells were harvested from each well and genomic DNA were isolated using the BD DNA Advance kit (Beckman Coulter) following the manufacturer’s instructions. The editing of B2M site was verified by sanger sequencing and colonies that have 100% editing at target position were used for WGS library prep. WGS library prep was performed using the Nextera flex genomic DNA library kit (Illumina) with 100–500 ng genomic DNA input and barcoded using Nextera CD 96 indexes (Illumina). The sequencing of WGS libraries were carried out at Novogene using NovaSeq S4 flow cells (Illumina). Data analysis was performed as described in Supplementary Note 5.

### Reporting summary

Further information on research design is available in the Nature Research Reporting Summary linked to this article.

## Supplementary information

Supplementary Information

Reporting Summary

## Data Availability

Core next-generation CBEs described in this work are deposited on Addgene. Figures 1c–f–5 and Supplementary Figs. 2–4, 6–10, 13, and 14b–16 are provided as Source Data files. High-throughput sequencing data are available in the NCBI Sequence Read Archive ([https://www.ncbi.nlm.nih.gov/sra/?term=PRJNA595157]). Any other relevant data are available from the authors upon reasonable request.

## Source data

Source Data

## Code availability

All software tools used for data analysis are publicly available. Detailed information about versions and parameters used, as well as shell commands, are provided in Supplementary Note 3–5.
