# Supplementary material for: Cytosine base editors with minimized unguided DNA and RNA off-target events and high on-target activity
Source: Nat Commun. 2020 Apr 28;11:2052. doi: 10.1038/s41467-020-15887-5 (PMC7189382; doi:10.1038/s41467-020-15887-5)
Supplement: Supplementary file 1 — Supplementary Information [file 41467_2020_15887_MOESM1_ESM.pdf]

Supplementary Information

Cytosine base editors with minimized unguided DNA and RNA off-target events and high on-target activity

Authors:

Yi Yu, Thomas C. Leete, David A. Born, Lauren Young, Luis A Barrera, Seung-Joo Lee, Holly A. Rees, Giuseppe Ciaramella*, Nicole M. Gaudelli*

*correspondence: ngaudelli@beamtx.com, gciaramella@beamtx.com

Affiliations: Beam Therapeutics (Cambridge, MA, USA)

Table of Contents

Supplementary Figure 1: rAPOBEC1 homology model generated by SWISSMODEL using hAPOBEC3C structure……………………………………………………………………………………………..……4

Supplementary Figure 2: *in cis/in trans* editing activities of BE4 with rAPOBEC1 mutants shown in Supplementary Figure 1 at site 1, 4, 6…...……...………………………...……………………………………5

Supplementary Figure 3: *in cis/in trans* editing activities of BE4-rAPOBEC1 with HiFi mutations at site 1 to 10………...………………………………………………………………………..…………..……6

Supplementary Figure 4: *in cis/in trans* editing activities of CBEs tested in 1st round screening at site 1, 4, 6…..…………………..………………………………………………………………………………….7

Supplementary Figure 5: Sequence alignment of CBEs tested in the 1st round screening…………...….9

Supplementary Figure 6: *in cis/in trans* activities of BE4-PpAPOBEC1 and BE4-PpAPOBEC with HiFi mutations at site 1 to 10….……………………………………………………………………………10

Supplementary Figure 7: *in cis/in trans* editing activities of CBEs tested in 2st round screening at site 1, 4, 6……11

Supplementary Figure 8: *in cis/in trans* editing activities of next generation CBEs at site 1 to 10……..……………………………………………………………………………………………….…13

Supplementary Figure 9: Editing window of CBEs shown in Supplementary Figure 8 at site 1 to 10……….………………………………………………………………………………………………….…14

Supplementary Figure 10: Indel rates of CBEs shown in Supplementary Figure 8 at site 1 to 10..………16

Supplementary Figure 11: Percentage of max C-to-T editing efficiency of CBEs tested in 3st round screening at site 1, or 24 (*).………………………………………………………………………………17

Supplementary Figure 12: Homology models of next generation CBEs based on existing crystal structures.………..……………………………………………………………………………………………18

Supplementary Figure 13: Guided off-target editing of selected next generation CBEs.…………………19

Supplementary Figure 14. Quantification of mRNA and protein concentration of base editors in HEK293T cells transfected with plasmids encoding base editors.………………...20

Table of Contents Continued

Supplementary Figure 15: Whole genome sequencing experiments to evaluate guide-independent cytidine deamination activity from base editors ………………………………………………………….....…21

Supplementary Figure 16: C to U editing efficiency of selected CBEs on ssDNA substrates in *in vitro* enzymatic assay…………………………………………………………………………………………….22

Supplementary Table 1: Amino acid sequences of all deaminases tested in this study…….………..…..23

Supplementary Table 2: DNA sequences of sgRNA used in this study……………………………..……..33

Supplementary Table 3: DNA sequences of oligos used in this study……………………………………….34

Supplementary Table 4: DNA sequences of mammalian expression plasmids for the core CBEs showed in this study.………………………………………………………………………………….……...……37

Supplementary Note 1: Methods of creating similarity network of cytidine deaminases...........................40

Supplementary Note 2: Discussion about protein expression level of base editors. ………...…....…..…41

Supplementary Note 3: Targeted NGS analysis details…………..……..…………………………...……...42

Supplementary Note 4: Transcriptome sequencing analysis methods ….………………………..………...44

Supplementary Note 5: Whole genome NGS analysis details.…..……………………….………...............45

References: ………………………………………………………………………………...……….…………..46

Supplementary Figure 1. rAPOBEC1 homology model generated by SWISSMODEL using hAPOBEC3C structure (PDB ID 3VM8). ssDNA from hAPOBEC3A structure (PDB ID 5SWW) is manually docked. **a,** mutations predicated to affect ssDNA binding. **b,** mutations predicted to affect catalytical activity.

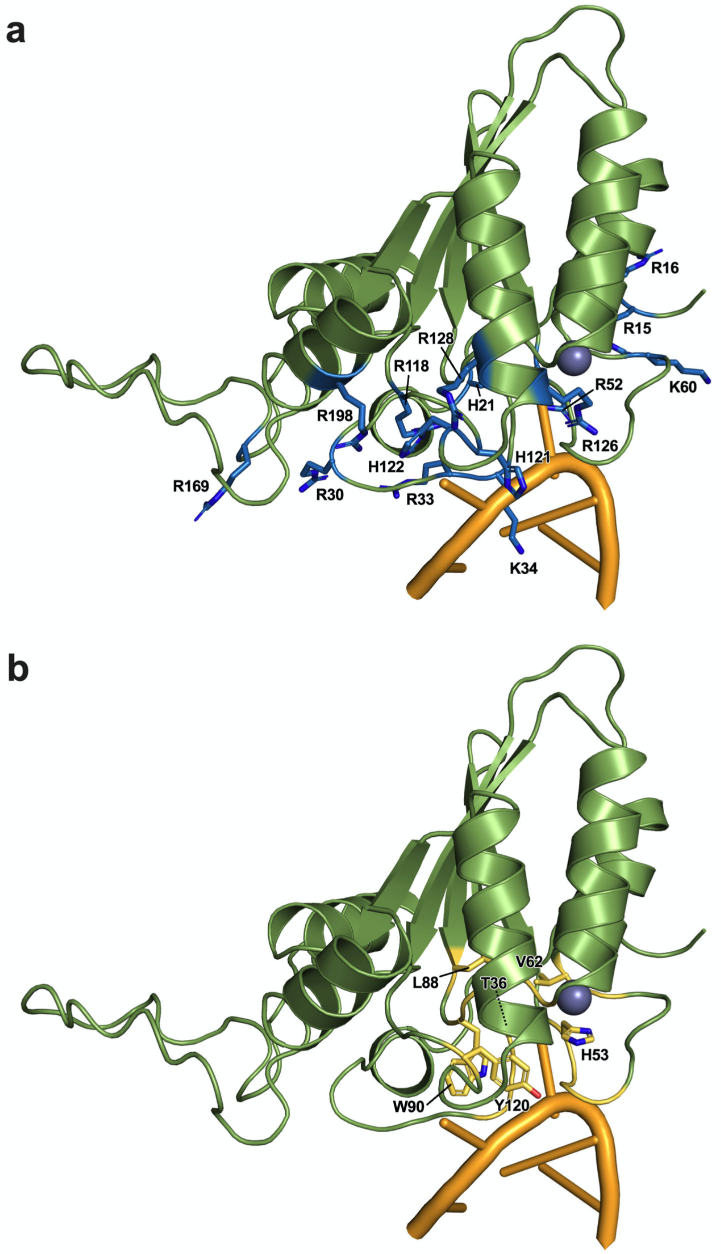

Supplementary Figure 2. *In cis* and *in trans* editing activities of BE4 with rAPOBEC1 mutants shown in Supplementary Figure 1 at site 1, 4, and 6. Base editing efficiencies were reported for the most edited base in the target sites. Values reflect the mean of n=3 independent biological replicates. All data presented are provided as source data.

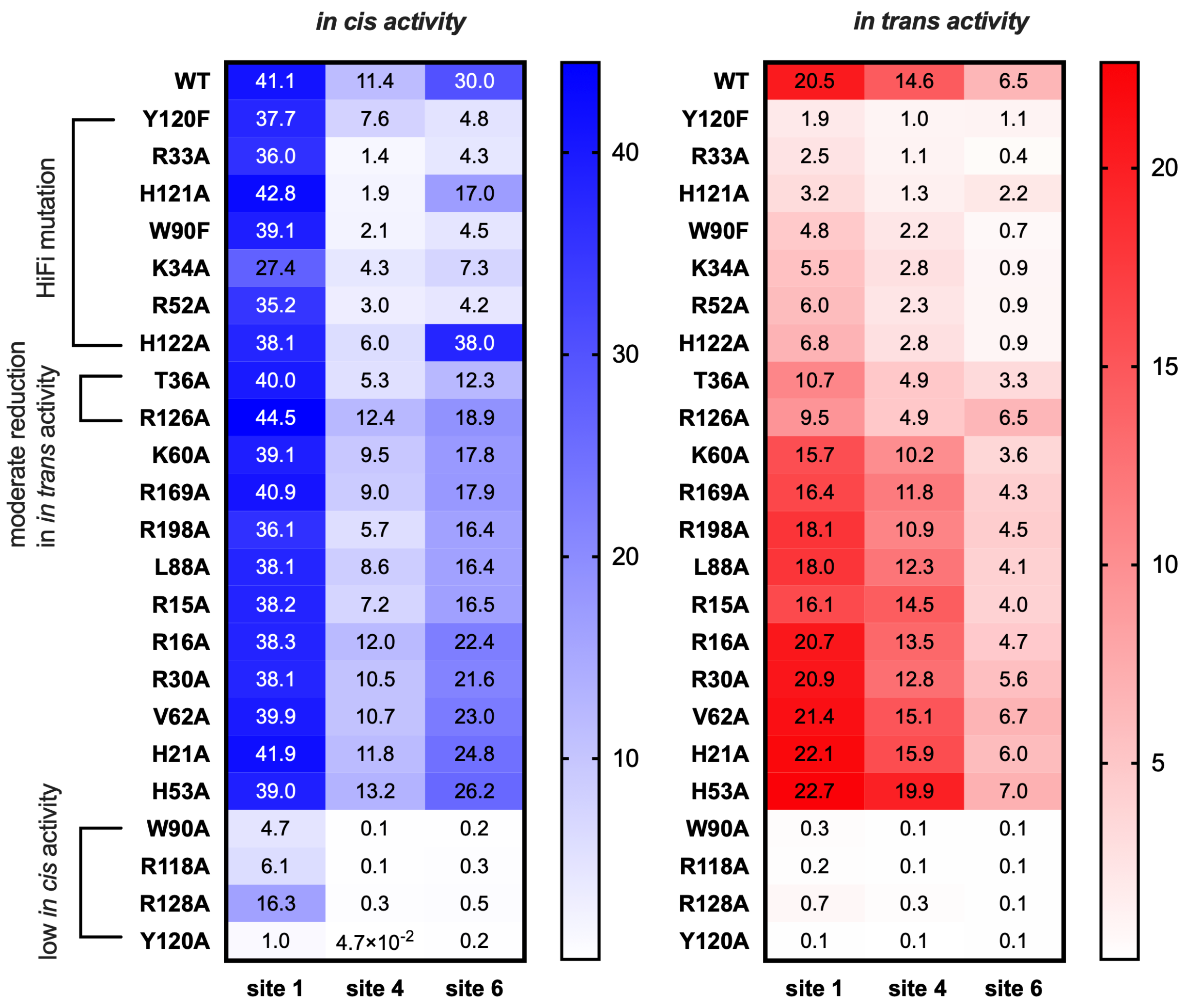

Supplementary Figure 3. *In cis/in trans* editing activities of BE4-rAPOBEC1 with HiFi mutations at site 1 to 10. Base editing efficiencies were reported for the most edited base in the target sites. Values and error bars reflect the mean and s.d. of n=4 independent biological replicates. All data presented are provided as source data.

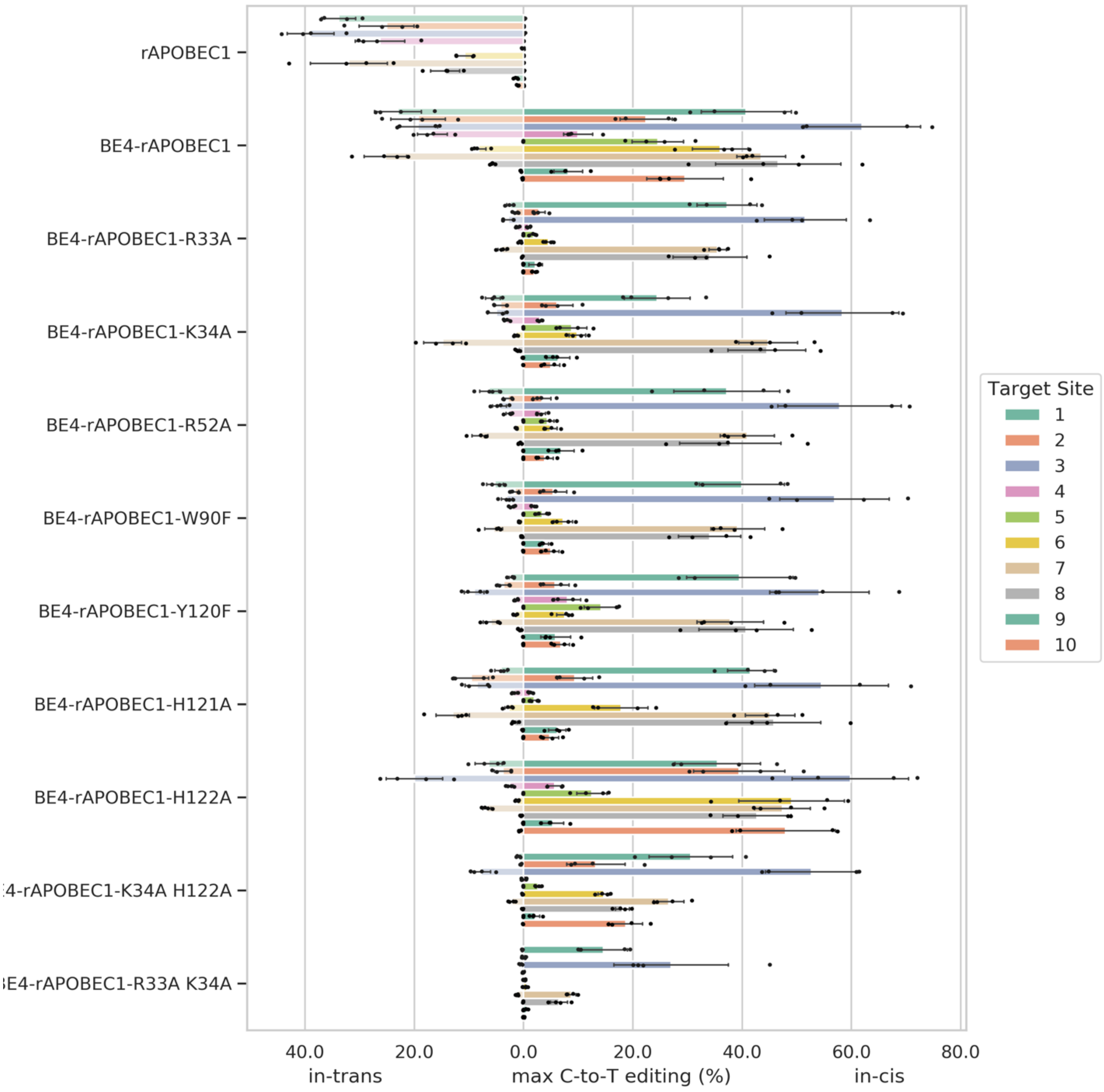

Supplementary Figure 4. *In cis/in trans* editing activities of CBEs tested in 1st round screening at site 1, 4, and 6. Base editing efficiencies were reported for the most edited base in the target sites. Values and error bars reflect the mean and s.d. of n=3 independent biological replicates. All data presented are provided as source data.

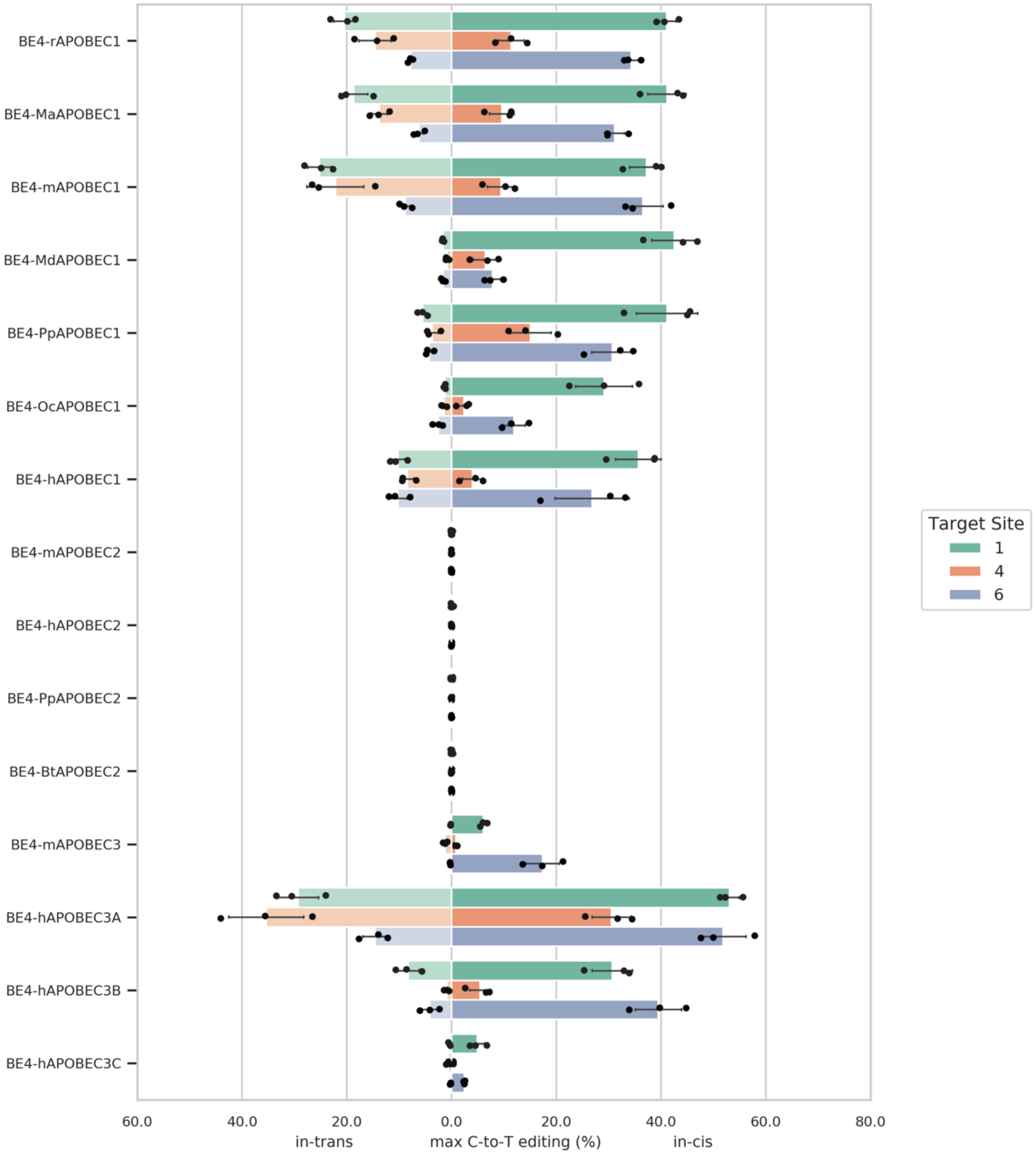

Supplementary Figure 4 continued. *In cis/in trans* editing activities of CBEs tested in 1st round screening at site 1, 4, 6. Base editing efficiencies were reported for the most edited base in the target sites. Values and error bars reflect the mean and s.d. of n=3 independent biological replicates. All data presented are provided as source data.

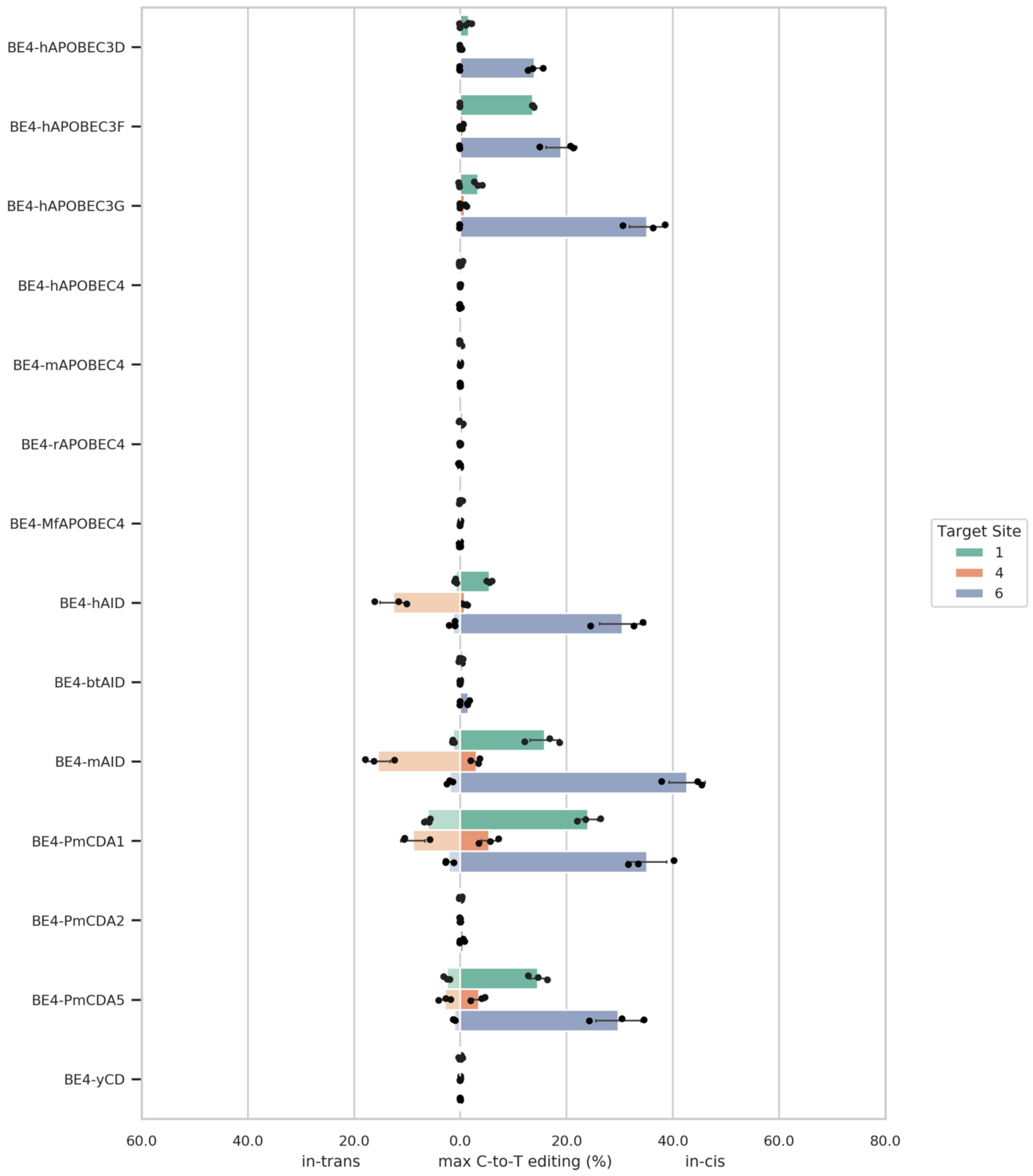

Supplementary Figure 5. *S*equence alignment of CBEs tested in the 1st round screening.

The amino acid residues that align to HiFi mutations in rAPOBEC1 are highlighted.

```
                 33                  52               90                       120
rAPOBEC1   -ELRKETCLLYEINWG...IWRHTSQNTNK...CSITWFLSWSPC...RYPHVTLFIYIARLYHHA-
MdAPOBEC1  -ELRKETCLLYEIKW-...IWRHSNQNTSQ...CSITWFLSWSPC...HYPNVTLAIFISRLYWHM-
MaAPOBEC1  -ELRKETCLLYEIRW-...IWRHTGQNTSR...CSIVWFLSWSPC...GHPNVTLFIYAARLYHHT-
mAPOBEC1   -ELRKETCLLYEINW-...VWRHTSQNTSN...CSITWFLSWSPC...RHPYVTLFIYIARLYHHT-
OcAPOBEC1  -ELRKEACLLYEIKW-...TWRSSGKNTTN...CSITWFLSWSPC...QHPGVTLIIFVARLFQHM-
hAPOBEC1   -ELRKEACLLYEIKW-...IWRSSGKNTTN...CSITWFLSWSPC...RHPGVTLVIYVARLFWHM-
PpAPOBEC1  -ELRKETCLLYEIKW-...IWRSSGKNTTN...CSITWFLSWSPC...QHPGVTLVIYVARLFWHW-
mAPOBEC3   -YHRMKPYLCYQLEQ-...---KGCLLSEK...VTITCYLTWSPC...DRPDLILHIYTSRLYFHW-
hAPOBEC3D  -CGRNESWLCFTMEV-...FRKRGVFRNQV...YEVTWYTSWSPC...RHSNVNLTIFTARLCYFW-
hAPOBEC3F  -YGRNESWLCFTMEV-...SWKRGVFRNQV...YEVTWYTSWSPC...RHSNVNLTIFTARLYYFW-
hAPOBEC3C  -NDRNETWLCFTVEG-...SWKTGVFRNQV...YQVTWYTSWSPC...RHSNVNLTIFTARLYYFQ-
hAPOBEC3G  -RGRHETYLCYEVER-...NQRRGFLCNQA...YRVTCFTSWSPC...KNKHVSLCIFTARIYDD--
hAPOBEC3A  -IGRHKTYLCYEVER-...DQHRGFLHNQA...YRVTWFISWSPC...ENTHVRLRIFAARIYDY--
hAPOBEC3B  -LRRRQTYLCYEVER-...DQHMGFLCNEA...YRVTWFISWSPC...ENTHVRLRIFAARIYDY--
rAPOBEC4   -TYPQTKHLTFYELR-...GLASNCTGSHT...RHIILYSNNSPC...NYPEVTLSVFFSQLYHTEM
hAPOBEC4   -TFPQTKHLTFYELK-...GHASSCTGNYI...RHIILYSNNSPC...TYPGITLSIYFSQLYHTEM
mAPOBEC4   -KGRHETYLCYVVKR-...SLDFGHLRNKS...YRVTWFTSWSPC...WNPNLSLRIFTARLYFCE-
MfAPOBEC4  -TYPQTKHLTFYELK-...GHASSCTGNYI...RHIILYCNNSPC...TYPGITLSIYFSQLYHTEM
PmCDA2     -QKPRGTVILFYVEG-...AVNYNKQGTSI...CTLHCYSTYSPC...-STGVRVVIHCCRIYELDV
PmCDA1     -TERHRTYVIFDVKP-...LW--GYIINNP...YAMTWYMSWSPC...EEQGHTLTMHFSRIYDRDR
PmCDA5     -TERHRTYVIFDVKP-...LW--GYIINNP...YAMTWYMSWSPC...EEQGHTLMMHFSRLYDRDR
hAID       -KGRRETYLCYVVKR-...SLDFGYLRNKN...YRVTWFTSWSPC...GNPNLSLRIFTARLYFCE-
mAID       -KGRRETYLCYVVKR-...SLDFGYLRNKN...YRVTWFTSWSPC...GNPNLSLRIFTARLYFCE-
BtAID      -KGRHETYLCYVVKR-...SLDFGHLRNKA...YRVTWFTSWSPC...GYPNLSLRIFTARLYFCDK
BtAPOBEC2  -SGRNKTFLCYVVEA-...QASRGYLEDEH...YMVTWYVSSSPC...KTKNLRLLILVGRLFMWE-
mAPOBEC2   -SGRNKTFLCYVVEV-...QATQGYLEDEH...YNVTWYVSSSPC...KTKNLRLLILVSRLFMWE-
hAPOBEC2   -SGRNKTFLCYVVEA-...QASRGYLEDEH...YNVTWYVSSSPC...KTKNLRLLILVGRLFMWE-
PpAPOBEC2  -SGRNKTFLCYVVEA-...QASRGYLEDEH...YNVTWYVSSSPC...KTKNLRLLILVGRLFMWE-
yCD        -----EAALGYKEGG-...NKDGSVLGRGH...KDTTLYTTLSPC...---GIPRCVVGENVNF---
```

Supplementary Figure 6. *In cis/in trans* activities of BE4-PpAPOBEC1 and BE4-PpAPOBEC with HiFi mutations at site 1 to 10. Base editing efficiencies were reported for the most edited base in the target sites. Values and error bars reflect the mean and s.d. of n=4 independent biological replicates. All data presented are provided as source data.

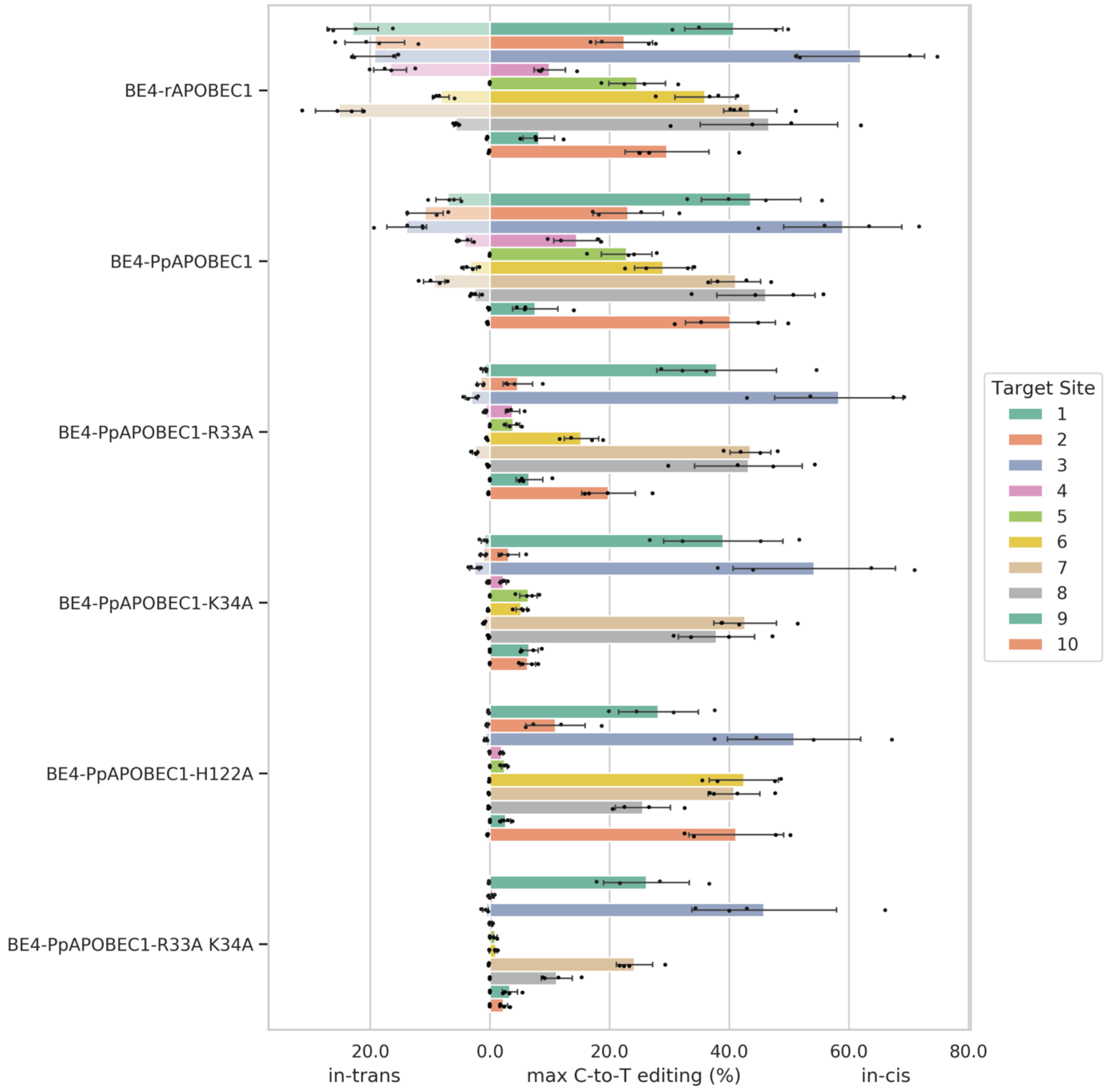

Supplementary Figure 7. *In cis/in trans* editing activities of CBEs tested in 2st round screening at site 1, 4, and 6. Base editing efficiencies were reported for the most edited base in the target sites. Values and error bars reflect the mean and s.d. of n=3 independent biological replicates. All data presented are provided as source data.

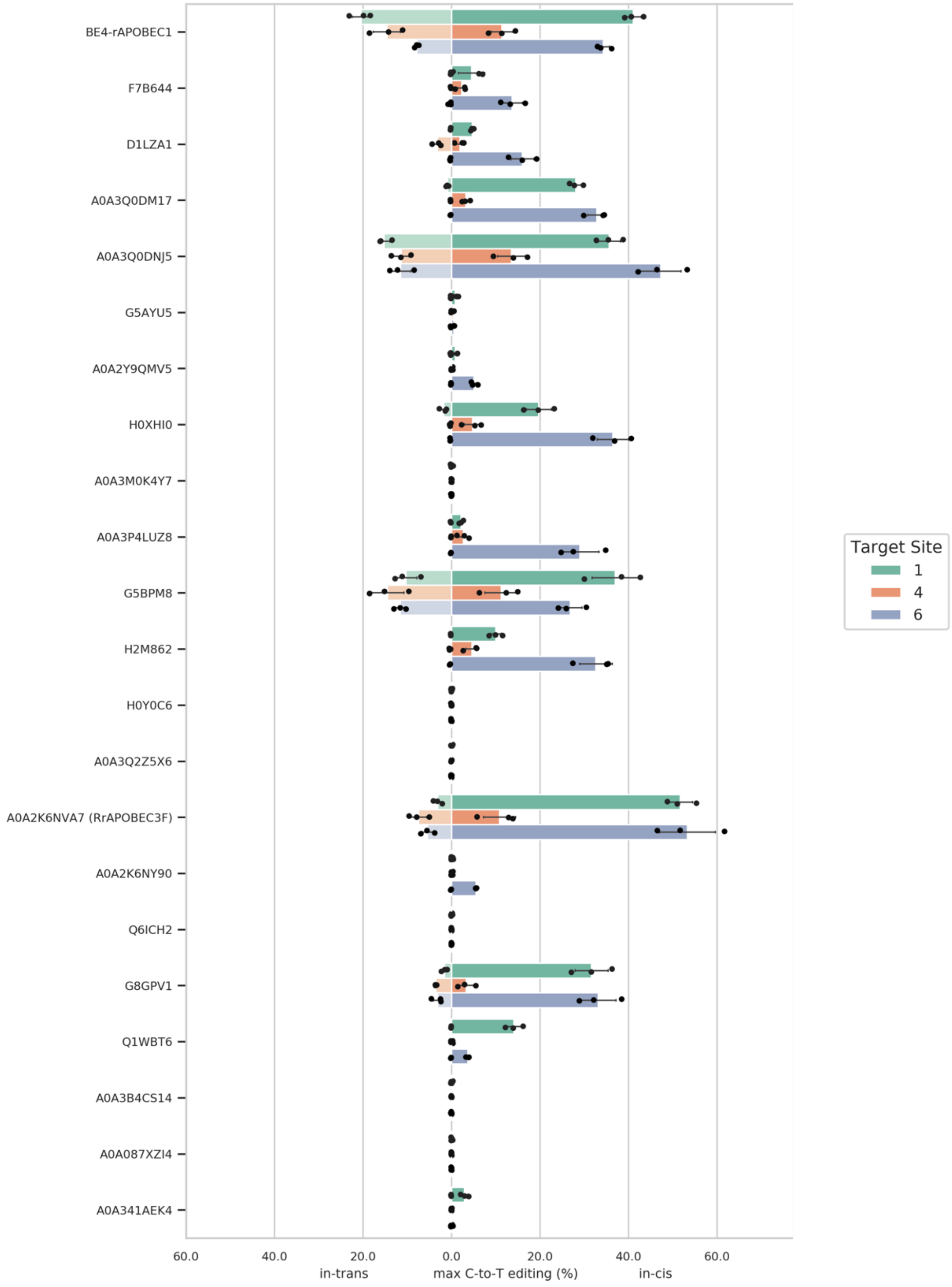

Supplementary Figure 7 continued. *In cis/in trans* editing activities of CBEs tested in 2st round screening at site 1, 4, and 6. Base editing efficiencies were reported for the most edited base in the target sites. Values and error bars reflect the mean and s.d. of n=3 independent biological replicates. All data presented are provided as source data.

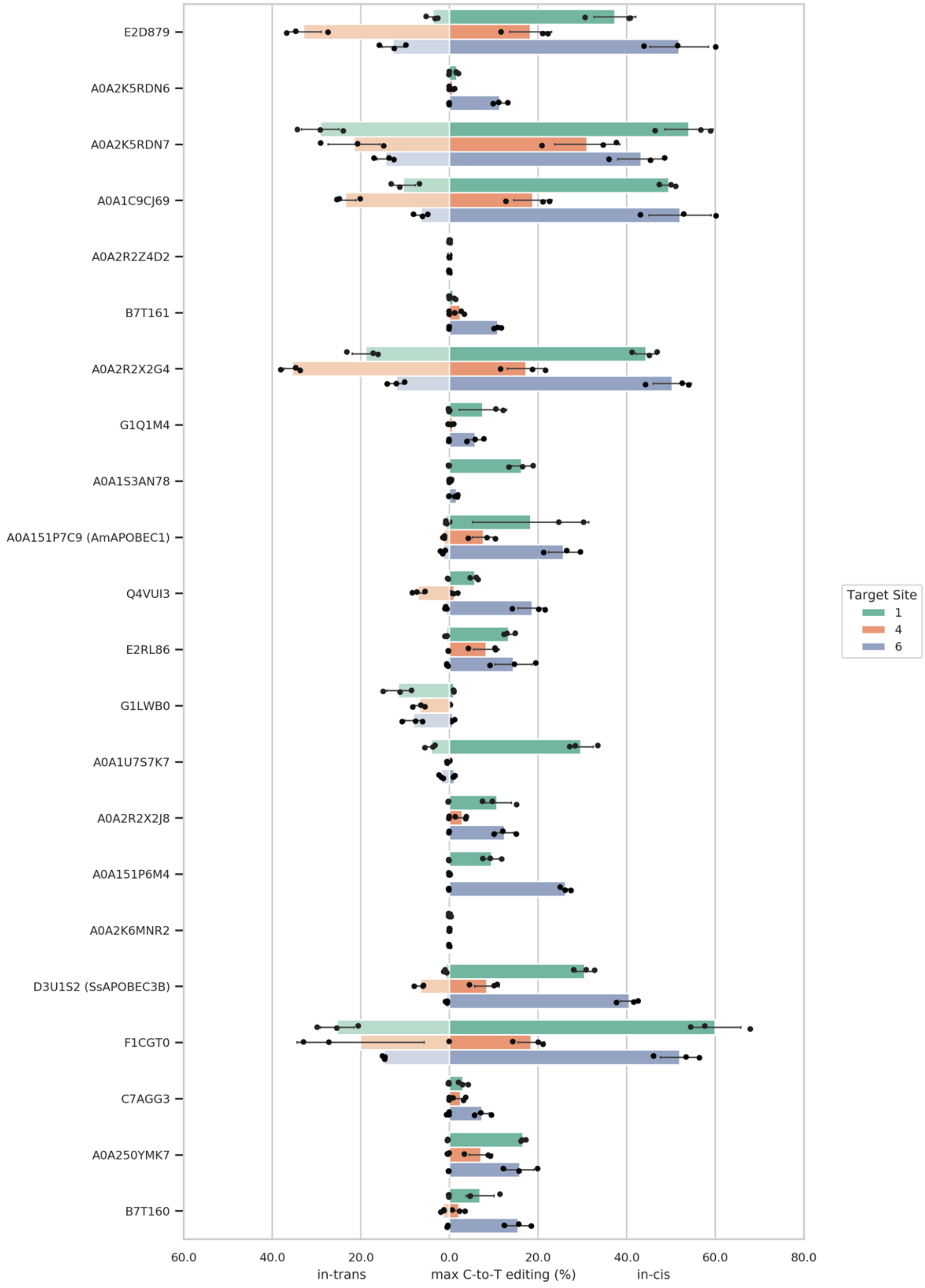

Supplementary Figure 8. *In cis/in trans* editing activities of next generation CBEs at site 1 to 10. Base editing efficiencies were reported for the most edited base in the target sites. Values and error bars reflect the mean and s.d. of n=4 independent biological replicates. All data presented are provided as source data.

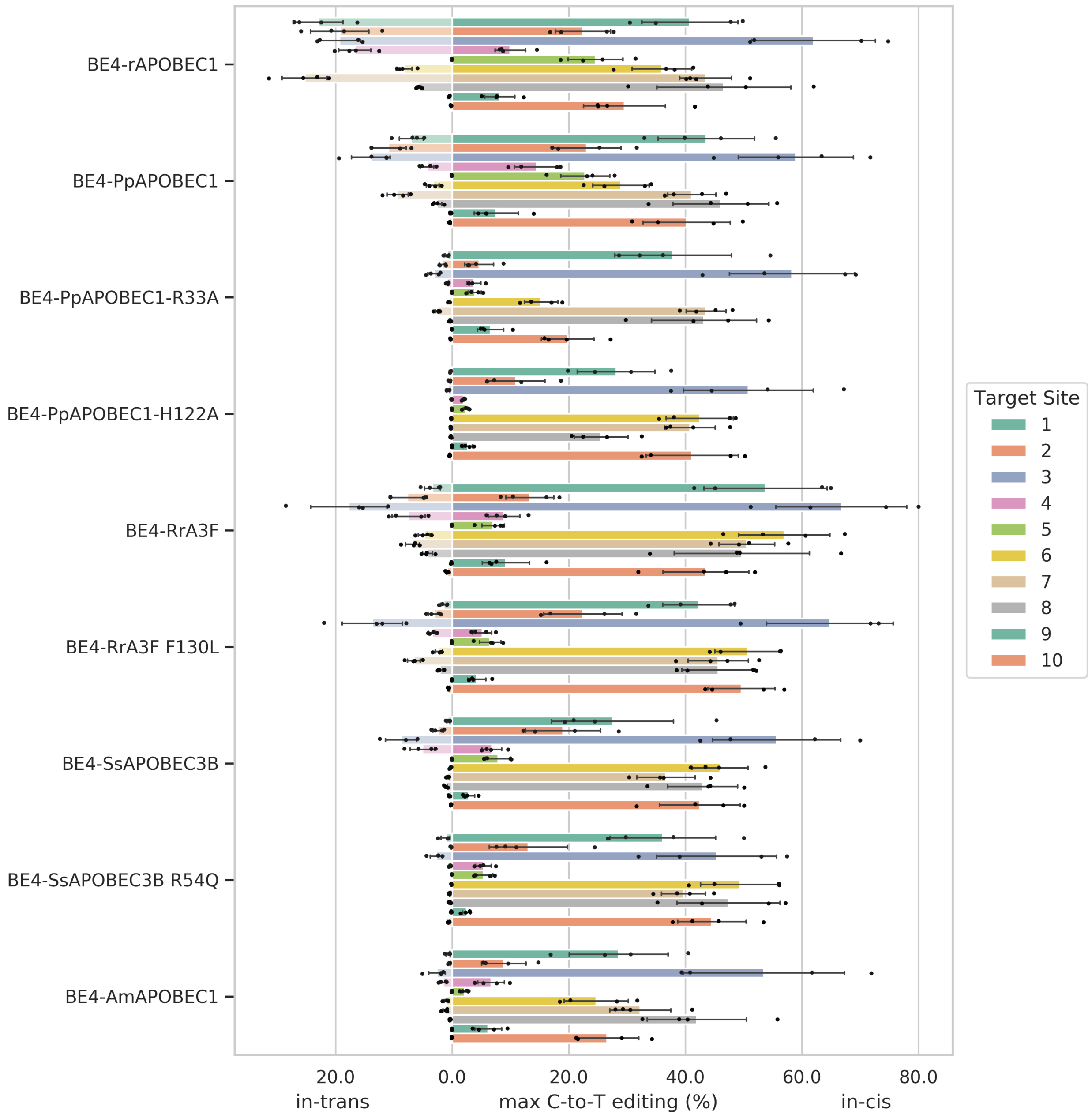

Supplementary Figure 9. Editing window of CBEs shown in Supplementary Figure 8 at site 1 to 10. Values reflect the mean of n=4 independent biological replicates. *In cis* and *in trans* editing are presented as blue and orange heatmaps respectively. All data presented are provided as source data.

site 1

	tC8t	aC11t	aC17t	tC8t	aC11t	aC17t
BE4-rAPOBEC1	40.76%	9.25%	0.10%	22.99%	1.23%	0.29%
BE4-PpAPOBEC1	43.62%	11.28%	0.21%	7.00%	1.35%	1.16%
BE4-PpAPOBEC1-R33A	37.89%	1.49%	0.02%	0.87%	0.07%	0.11%
BE4-PpAPOBEC1-H122A	28.15%	1.46%	0.02%	0.26%	0.06%	0.07%
BE4-RrA3F	53.76%	4.39%	0.02%	3.39%	0.53%	0.23%
BE4-RrA3F F130L	42.27%	7.46%	0.04%	1.40%	0.37%	0.15%
BE4-SsAPOBEC3B	27.50%	5.47%	0.25%	0.66%	0.09%	0.05%
BE4-SsAPOBEC3B R54Q	36.13%	2.33%	0.07%	1.12%	0.04%	0.05%
BE4-AmAPOBEC1	26.75%	28.55%	2.50%	0.13%	0.65%	0.38%

site 2

	gC2a	aC4c	cC5c	cC6a	tC14t	gC17a	gC2a	aC4c	cC5c	cC6a	tC14t	gC17a
BE4-rAPOBEC1	0.32%	8.05%	10.91%	18.19%	22.44%	0.07%	0.02%	0.06%	0.06%	0.29%	19.28%	0.08%
BE4-PpAPOBEC1	0.45%	7.30%	7.41%	10.31%	23.07%	0.08%	0.02%	0.05%	0.03%	0.06%	10.89%	0.15%
BE4-PpAPOBEC1-R33A	0.08%	1.48%	1.60%	3.30%	4.64%	0.04%	0.02%	0.02%	0.03%	0.03%	1.62%	0.08%
BE4-PpAPOBEC1-H122A	3.33%	0.78%	3.04%	10.94%	1.16%	0.53%	0.03%	0.01%	0.03%	0.07%	0.27%	0.46%
BE4-RrA3F	3.57%	1.47%	6.43%	8.48%	13.34%	5.21%	0.08%	0.04%	0.27%	0.12%	7.62%	1.84%
BE4-RrA3F F130L	9.10%	4.78%	17.59%	22.45%	6.10%	4.39%	0.07%	0.01%	0.14%	0.09%	3.04%	0.80%
BE4-SsAPOBEC3B	18.75%	13.22%	18.53%	17.01%	11.58%	4.76%	0.06%	0.07%	0.16%	0.14%	1.23%	2.39%
BE4-SsAPOBEC3B R54Q	10.54%	9.33%	13.05%	11.85%	4.70%	0.82%	0.06%	0.04%	0.11%	0.09%	0.18%	0.15%
BE4-AmAPOBEC1	0.53%	1.00%	2.53%	5.38%	8.88%	2.03%	0.03%	0.03%	0.02%	0.08%	0.46%	0.19%

site 3

	gC2a	tC6c	cC7a	aC9t	tC11c	cC12g	tC15c	cC16g	gC18c	gC2a	tC6c	cC7a	aC9t	tC11c	cC12g	tC15c	cC16g	gC18c
BE4-rAPOBEC1	0.13%	59.18%	61.98%	30.28%	26.37%	8.34%	3.88%	0.74%	0.01%	0.03%	19.12%	18.67%	13.95%	18.93%	17.33%	9.56%	2.43%	0.03%
BE4-PpAPOBEC1	0.08%	55.05%	58.98%	44.33%	13.33%	3.79%	2.84%	0.15%	0.04%	0.03%	13.95%	5.88%	7.66%	9.19%	3.80%	8.07%	1.82%	0.01%
BE4-PpAPOBEC1-R33A	0.07%	56.63%	58.23%	15.98%	6.85%	1.47%	0.57%	0.05%	0.02%	0.03%	3.12%	0.41%	0.83%	1.55%	0.53%	1.66%	0.60%	0.01%
BE4-PpAPOBEC1-H122A	2.74%	50.84%	40.99%	14.04%	1.88%	0.99%	0.40%	0.07%	0.36%	0.06%	0.72%	0.50%	0.40%	0.20%	0.32%	0.23%	0.63%	0.16%
BE4-RrA3F	0.67%	63.30%	66.77%	24.05%	10.51%	3.35%	0.49%	0.25%	0.06%	0.04%	17.69%	9.48%	1.03%	2.96%	6.14%	5.14%	1.54%	0.08%
BE4-RrA3F F130L	1.57%	60.15%	64.80%	35.41%	10.59%	3.76%	0.37%	0.28%	0.14%	0.03%	13.68%	5.75%	0.41%	1.27%	5.73%	2.42%	1.07%	0.05%
BE4-SsAPOBEC3B	23.00%	53.35%	55.63%	15.28%	25.13%	26.36%	10.53%	4.85%	1.72%	0.24%	8.76%	2.63%	0.24%	0.52%	2.03%	1.17%	2.52%	0.09%
BE4-SsAPOBEC3B R54Q	14.58%	45.37%	31.87%	8.68%	21.18%	23.29%	8.77%	0.39%	0.56%	0.15%	2.39%	1.56%	0.48%	0.31%	0.42%	1.31%	1.71%	0.06%
BE4-AmAPOBEC1	0.32%	52.48%	52.88%	37.56%	11.59%	13.07%	1.04%	0.55%	0.05%	0.02%	1.21%	1.42%	0.51%	0.68%	2.60%	0.29%	0.67%	0.03%

site 4

	gC2c	cC3a	aC5a	aC9t	tC14c	cC15a	gC2c	cC3a	aC5a	aC9t	tC14c	cC15a
BE4-rAPOBEC1	0.10%	1.70%	9.98%	6.16%	4.18%	0.70%	0.02%	0.03%	0.07%	3.43%	16.69%	2.60%
BE4-PpAPOBEC1	0.15%	0.98%	14.52%	7.41%	5.57%	0.45%	0.03%	0.02%	0.14%	1.48%	4.29%	0.36%
BE4-PpAPOBEC1-R33A	0.03%	0.22%	3.76%	0.58%	0.42%	0.02%	0.03%	0.03%	0.04%	0.12%	0.76%	0.05%
BE4-PpAPOBEC1-H122A	0.27%	0.42%	1.92%	0.29%	0.13%	0.03%	0.03%	0.04%	0.03%	0.07%	0.10%	0.04%
BE4-RrA3F	0.75%	0.19%	8.93%	6.49%	1.15%	0.22%	0.04%	0.02%	0.03%	0.41%	7.46%	0.51%
BE4-RrA3F F130L	2.68%	1.37%	5.16%	0.78%	0.44%	0.09%	0.02%	0.01%	0.02%	0.08%	3.44%	0.18%
BE4-SsAPOBEC3B	6.82%	3.38%	3.19%	1.16%	0.84%	0.28%	0.03%	0.05%	0.04%	0.03%	5.10%	2.11%
BE4-SsAPOBEC3B R54Q	5.38%	1.92%	3.18%	0.46%	0.16%	0.08%	0.02%	0.02%	0.03%	0.04%	0.33%	0.14%
BE4-AmAPOBEC1	0.25%	0.67%	3.85%	1.25%	6.71%	3.89%	0.03%	0.02%	0.03%	0.08%	1.56%	0.20%

site 5

	gC2c	cC3a	aC5a	aC18a	gC2c	cC3a	aC5a	aC18a
BE4-rAPOBEC1	0.10%	0.90%	24.59%	0.04%	0.03%	0.03%	0.05%	0.03%
BE4-PpAPOBEC1	0.11%	0.30%	22.82%	0.03%	0.03%	0.02%	0.07%	0.01%
BE4-PpAPOBEC1-R33A	0.04%	0.10%	3.88%	0.02%	0.03%	0.02%	0.02%	0.03%
BE4-PpAPOBEC1-H122A	0.25%	0.29%	2.43%	0.02%	0.03%	0.01%	0.03%	0.02%
BE4-RrA3F	1.31%	0.34%	7.04%	0.02%	0.03%	0.01%	0.03%	0.02%
BE4-RrA3F F130L	5.29%	3.27%	6.52%	0.03%	0.04%	0.01%	0.03%	0.02%
BE4-SsAPOBEC3B	7.91%	5.02%	3.97%	0.02%	0.03%	0.02%	0.04%	0.01%
BE4-SsAPOBEC3B R54Q	5.41%	3.41%	3.01%	0.01%	0.04%	0.03%	0.02%	0.02%
BE4-AmAPOBEC1	0.29%	0.39%	2.11%	0.02%	0.02%	0.03%	0.03%	0.03%

Supplementary Figure 9 continued. Editing window of CBEs shown in Supplementary Figure 8 at site 1 to 10. Values reflect the mean of n=4 independent biological replicates. *In cis* and *in trans* editing are presented as blue and orange heatmaps respectively. All data presented are provided as source data.

	gC2c	cC3c	cC4a	gC7a	tC12a	aC14t
BE4-rAPOBEC1	0.41%	7.44%	22.23%	14.79%	35.98%	1.38%
BE4-PpAPOBEC1	0.39%	1.27%	5.25%	26.30%	28.97%	2.82%
BE4-PpAPOBEC1-R33A	0.19%	0.96%	2.00%	10.82%	15.28%	0.40%
BE4-PpAPOBEC1-H122A	2.52%	1.15%	9.68%	42.45%	1.10%	0.15%
BE4-RrA3F	0.16%	0.27%	11.42%	56.94%	10.59%	0.09%
BE4-RrA3F F130L	0.89%	3.65%	39.64%	50.71%	8.67%	0.24%
BE4-SsAPOBEC3B	16.64%	20.56%	41.25%	45.98%	18.02%	2.50%
BE4-SsAPOBEC3B R54Q	31.02%	28.51%	38.82%	49.43%	4.61%	2.77%
BE4-AmAPOBEC1	0.60%	2.22%	8.37%	21.39%	24.69%	6.76%

	gC2c	cC3c	cC4a	gC7a	tC12a	aC14t
BE4-rAPOBEC1	0.02%	0.02%	0.02%	0.04%	8.22%	0.41%
BE4-PpAPOBEC1	0.03%	0.02%	0.03%	0.03%	3.32%	0.37%
BE4-PpAPOBEC1-R33A	0.02%	0.02%	0.05%	0.02%	0.55%	0.05%
BE4-PpAPOBEC1-H122A	0.02%	0.02%	0.02%	0.04%	0.14%	0.03%
BE4-RrA3F	0.03%	0.02%	0.03%	0.69%	4.77%	0.07%
BE4-RrA3F F130L	0.02%	0.01%	0.03%	0.48%	2.28%	0.02%
BE4-SsAPOBEC3B	0.04%	0.05%	0.06%	0.13%	0.27%	0.06%
BE4-SsAPOBEC3B R54Q	0.03%	0.01%	0.02%	0.11%	0.07%	0.03%
BE4-AmAPOBEC1	0.01%	0.15%	0.23%	0.12%	1.00%	0.04%

site 6

	gC2c	cC3c	cC4a	gC7t	tC9c	cC10a	gC13c	cC14t	tC16t
BE4-rAPOBEC1	0.84%	11.50%	20.34%	20.52%	43.49%	41.11%	0.08%	0.28%	5.63%
BE4-PpAPOBEC1	1.15%	3.42%	8.73%	35.45%	41.09%	38.31%	0.14%	0.10%	5.81%
BE4-PpAPOBEC1-R33A	0.38%	2.64%	4.31%	23.01%	43.56%	18.97%	0.06%	0.08%	1.11%
BE4-PpAPOBEC1-H122A	7.35%	5.56%	18.61%	40.80%	28.41%	9.29%	1.86%	0.09%	0.23%
BE4-RrA3F	15.41%	5.30%	18.08%	50.53%	48.48%	33.97%	4.22%	0.16%	0.21%
BE4-RrA3F F130L	30.94%	16.65%	36.21%	45.47%	44.35%	38.21%	7.87%	0.08%	0.18%
BE4-SsAPOBEC3B	36.67%	31.98%	35.43%	35.63%	34.04%	33.34%	10.09%	1.88%	1.05%
BE4-SsAPOBEC3B R54Q	39.09%	29.70%	29.53%	39.63%	24.95%	13.94%	13.41%	6.94%	0.26%
BE4-AmAPOBEC1	13.89%	3.49%	11.40%	19.31%	25.11%	32.26%	0.78%	0.21%	0.24%

	gC2c	cC3c	cC4a	gC7t	tC9c	cC10a	gC13c	cC14t	tC16t
BE4-rAPOBEC1	0.02%	0.05%	0.04%	0.04%	25.29%	9.78%	0.04%	0.13%	4.39%
BE4-PpAPOBEC1	0.03%	0.03%	0.02%	0.05%	6.66%	0.98%	0.03%	0.03%	9.35%
BE4-PpAPOBEC1-R33A	0.02%	0.02%	0.03%	0.03%	1.80%	0.07%	0.02%	0.03%	2.50%
BE4-PpAPOBEC1-H122A	0.03%	0.02%	0.03%	0.12%	0.15%	0.10%	0.20%	0.04%	0.19%
BE4-RrA3F	0.18%	0.03%	0.03%	2.25%	2.21%	3.27%	6.81%	0.36%	0.33%
BE4-RrA3F F130L	0.10%	0.02%	0.02%	1.81%	1.14%	2.51%	6.55%	0.11%	0.13%
BE4-SsAPOBEC3B	0.54%	0.08%	0.28%	0.73%	0.17%	0.46%	0.52%	0.03%	0.15%
BE4-SsAPOBEC3B R54Q	0.18%	0.09%	0.12%	0.48%	0.11%	0.10%	0.22%	0.03%	0.06%
BE4-AmAPOBEC1	0.03%	0.03%	0.02%	0.03%	0.12%	1.16%	0.08%	0.03%	0.03%

site 7

	gC2c	cC3c	cC4t	tC9t	gC12a	aC14t
BE4-rAPOBEC1	0.34%	5.35%	20.66%	46.61%	0.32%	2.24%
BE4-PpAPOBEC1	0.41%	1.47%	9.72%	46.12%	0.60%	5.95%
BE4-PpAPOBEC1-R33A	0.15%	0.95%	2.91%	43.21%	0.23%	0.46%
BE4-PpAPOBEC1-H122A	4.07%	2.10%	17.92%	25.52%	8.71%	0.38%
BE4-RrA3F	13.33%	3.58%	12.92%	49.70%	11.68%	0.56%
BE4-RrA3F F130L	25.08%	12.50%	34.06%	45.67%	12.46%	0.30%
BE4-SsAPOBEC3B	42.96%	34.92%	37.29%	30.03%	33.83%	2.23%
BE4-SsAPOBEC3B R54Q	47.39%	28.91%	27.57%	21.98%	16.94%	3.88%
BE4-AmAPOBEC1	12.35%	2.95%	9.30%	22.00%	41.95%	19.82%

	gC2c	cC3c	cC4t	tC9t	gC12a	aC14t
BE4-rAPOBEC1	0.02%	0.02%	0.04%	5.71%	0.07%	0.70%
BE4-PpAPOBEC1	0.07%	0.07%	0.04%	2.57%	0.10%	1.89%
BE4-PpAPOBEC1-R33A	0.03%	0.03%	0.03%	0.34%	0.02%	0.07%
BE4-PpAPOBEC1-H122A	0.09%	0.02%	0.04%	0.13%	0.23%	0.07%
BE4-RrA3F	0.24%	0.07%	0.07%	1.12%	4.21%	0.27%
BE4-RrA3F F130L	0.17%	0.04%	0.08%	0.64%	2.08%	0.05%
BE4-SsAPOBEC3B	1.02%	0.10%	0.21%	0.17%	0.98%	0.07%
BE4-SsAPOBEC3B R54Q	0.35%	0.03%	0.11%	0.08%	0.30%	0.07%
BE4-AmAPOBEC1	0.05%	0.03%	0.04%	0.10%	0.36%	0.06%

site 8

	gC2c	cC3t	tC5a	tC10t
BE4-rAPOBEC1	0.37%	2.09%	8.17%	7.78%
BE4-PpAPOBEC1	0.30%	0.84%	7.55%	6.88%
BE4-PpAPOBEC1-R33A	0.08%	0.44%	6.55%	4.51%
BE4-PpAPOBEC1-H122A	1.07%	0.61%	2.66%	1.38%
BE4-RrA3F	3.43%	0.58%	8.43%	9.23%
BE4-RrA3F F130L	3.50%	1.64%	4.20%	4.06%
BE4-SsAPOBEC3B	2.79%	2.23%	2.66%	2.08%
BE4-SsAPOBEC3B R54Q	2.47%	1.57%	2.37%	2.05%
BE4-AmAPOBEC1	3.24%	0.56%	3.16%	6.20%

	gC2c	cC3t	tC5a	tC10t
BE4-rAPOBEC1	0.02%	0.04%	0.34%	0.51%
BE4-PpAPOBEC1	0.03%	0.01%	0.24%	0.22%
BE4-PpAPOBEC1-R33A	0.02%	0.02%	0.04%	0.03%
BE4-PpAPOBEC1-H122A	0.01%	0.02%	0.02%	0.03%
BE4-RrA3F	0.03%	0.02%	0.03%	0.13%
BE4-RrA3F F130L	0.03%	0.02%	0.03%	0.03%
BE4-SsAPOBEC3B	0.49%	0.15%	0.25%	0.06%
BE4-SsAPOBEC3B R54Q	0.14%	0.07%	0.09%	0.03%
BE4-AmAPOBEC1	0.02%	0.03%	0.02%	0.02%

site 9

	gC2c	cC3t	gC7a	aC17c	cC18a
BE4-rAPOBEC1	1.21%	17.42%	29.56%	0.13%	0.05%
BE4-PpAPOBEC1	1.20%	6.42%	40.21%	0.06%	0.03%
BE4-PpAPOBEC1-R33A	0.34%	2.41%	19.80%	0.03%	0.03%
BE4-PpAPOBEC1-H122A	5.32%	5.39%	41.15%	0.03%	0.02%
BE4-RrA3F	20.07%	9.40%	43.52%	0.05%	0.04%
BE4-RrA3F F130L	35.34%	19.31%	49.60%	0.06%	0.05%
BE4-SsAPOBEC3B	42.50%	37.03%	38.66%	0.07%	0.07%
BE4-SsAPOBEC3B R54Q	31.44%	27.83%	44.55%	0.04%	0.05%
BE4-AmAPOBEC1	1.59%	3.39%	26.56%	0.05%	0.03%

	gC2c	cC3t	gC7a	aC17c	cC18a
BE4-rAPOBEC1	0.02%	0.03%	0.16%	0.08%	0.03%
BE4-PpAPOBEC1	0.02%	0.05%	0.10%	0.42%	0.04%
BE4-PpAPOBEC1-R33A	0.03%	0.02%	0.04%	0.28%	0.04%
BE4-PpAPOBEC1-H122A	0.03%	0.03%	0.43%	0.04%	0.03%
BE4-RrA3F	0.03%	0.03%	0.83%	0.04%	0.04%
BE4-RrA3F F130L	0.03%	0.02%	0.62%	0.03%	0.04%
BE4-SsAPOBEC3B	0.09%	0.04%	0.18%	0.03%	0.03%
BE4-SsAPOBEC3B R54Q	0.05%	0.04%	0.57%	0.02%	0.04%
BE4-AmAPOBEC1	0.03%	0.02%	0.06%	0.04%	0.03%

site 10

Supplementary Figure 10. Indel rates of CBEs shown in Supplementary Figure 8 at site 1 to 10. Values used to generate the heatmap reflect the mean of n=4 independent biological replicates. All data presented are provided as source data.

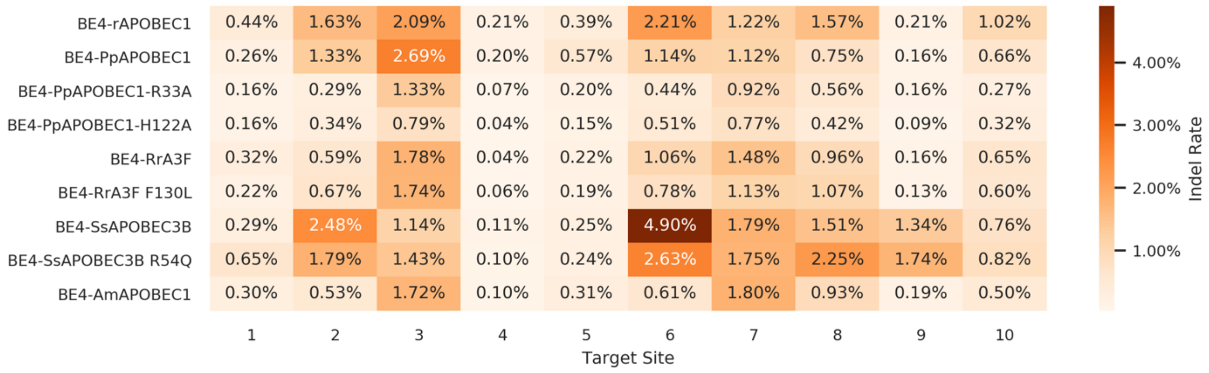

Supplementary Figure 11. Percentage of max C-to-T editing efficiency of CBEs tested in 3rd round screening at site 1, or 24 (*). Data is from single experiment with no replication. The percentage of max C-to-T for the control (BE4-rAPOBEC1) at site 1 was 40.1% and *48.2% at site 24. Star symbol indicates editing efficiency was measured at site 24, while no star indicates editing efficiency was measured at site 1. Uniprot IDs for the deaminases in CBEs are listed.

Deaminase ID	max C-to-T	Deaminase ID	max C-to-T	Deaminase ID	max C-to-T	Deaminase ID	max C-to-T
A0A182D0J1	*0.03	U7QZM1	0.173	A0A1I7EYS3	0.076	A0A2W0H8Y3	0.094
A0A2D6EXD2	*0.031	A0A081CH48	0.322	H8GQX8	0.143	A0A261BDB7	0.068
F7YVM7	*0.064	A0A3D3HMU1	0.216	A0A0S8HZN3	0.071	A0A2E1PHI6	0.06
A0A3M6UNF1	*0.049	A0A1N5WT13	0.207	E3SF31	0.159	A0A378LUA7	0.091
A0A2G3K826	*0.053	X0SAC5	0.048	F8AAC6	0.058	A0A139HQ78	0.752
K1ZCJ4	0.06	A0A3B8IC10	0.05	A0A2H4ZNK4	0.08	A0A2A9FXV0	0.089
A0A1G3PNQ8	0.042	A0A2N9P8B9	0.032	A0A239N5N1	0.039	A0A1A8AG96	0.056
A0A1G0PGF4	0.089	K1KX30	0.077	A0A328VTR2	0.039	A0A3E2VN88	0.042
A0A0P4WGY5	0.047	R4XI84	0.141	A0A103YG48	0.292	A0A2D5ZRJ2	0.169
A0A3D8IG27	0.04	A0A239CVF7	0.127	W5M1M8	0.061	A0A1B8WPS3	0.206
A0A351C8C4	0.103	A0A1Q3NME1	0.075	A0A3N5YPZ2	0.097	A0A1W5ZQK9	0.066
A0A1G6V2K7	0.067	A0A2G6N4N7	0.054	A0A2A9NC86	0.036	A0A378V0W4	0.093
F2NP91	0.018	A0A0G0RBB8	0.023	A0A2D6RD43	0.067	I3XF03	0.051
A0A316TX77	0.043	A0A327L2Q5	0.133	A0A0H3AVL6	0.07	F8IEF3	0.074
R6VYG3	0.03	S2DR30	0.029	A0A242H531	0.117	A0A1G3M638	0.017
A0A3C1HZ18	0.029	A0A369QGF1	0.065	A0A2R6XZE2	0.131	A0A3D9LFR2	0.083
A0A1M6KV24	0.054	A0A1W6X4U4	0.046	A0A139SHT6	0.029	A0A3B9YGB5	0.156
A0A2U0T9B4	0.107	A0A238BW09	0.149	A0A261DBH2	0.077	A0A182F569	0.066
A0A2K9PN08	0.096	A0A1J5H6Z0	0.05	A0A2N0XZK6	0.641	A0A264Z0D4	0.051
F4PWM7	0.033	A0A3C2D945	0.005	A0A1V5R0F9	0.225	A0A1L9Q1R3	0.201

Supplementary Figure 12. Homology models of selected four cytidine deaminases based on existing crystal structures. **a**: homology model of PpAPOBEC1 is based on based on a putative APOBEC3G structure (PDB ID 5K81); RrA3F is based on Vif-binding Domain of hAPOBEC3F (PDB ID 3WUS); AmAPOBEC1 is based on a hAPOBEC3B N-terminal domain (PDB ID 5TKM); SsAPOBEC3B is based on Vif-binding Domain of hAPOBEC3F (PDB ID 3WUS). **b**: sequence alignment of the selected four cytidine deaminases with rAPOBEC1.

a

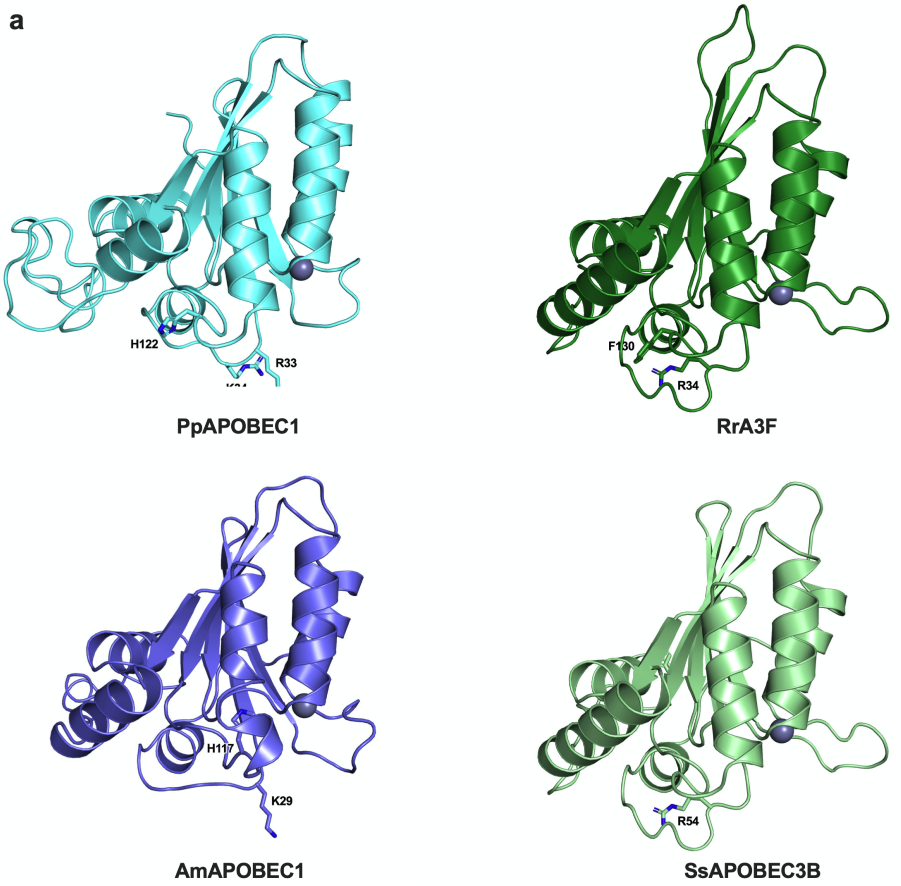

b

```
                 33                     52                      90                   120
rAPOBEC1    -LRKETCLLYEI...NGRHSIWRHTSQNT...SITWFLSWSPCGEC...YIARLYHHADP   59
PpAPOBEC1   -LRKETCLLYEI...KMSRKIWRSSGKNT...SITWFLSWSPCWEC...YVARLFWHMDQ   59
SsAPOBEC3B  SGRNRSYICCQV..EFFQGIFQNQVPPD...YVTWFISWSPCCEC...SAARLYYFWKS   60
AmAPOBEC1   -GTKEAHLLCEI...KKYGKPWLHWCQNQ...YVTWYLSWSPCADC...YVAQLYYHTEE   59
RfA3F       YGRNETWLCFTV...E--KGVFRNQVDPE...QVTWYTSWSPCPEC...YTARLYYFWDT   58
```

Supplementary Figure 13. Guided off-target editing of selected next generation CBEs. **a**, editing efficiency of next generation CBEs on HEK2, HEK3, HEK4 sites and **b**, reported guided off-target sites for HEK2 sgRNA, **c,** HEK3 sgRNA and **d,** HEK4 sgRNA. Base editing efficiencies were reported for the most edited base in the target sites. Values and error bars reflect the mean and s.d. of n=3 independent biological replicates. All data presented are provided as source data.

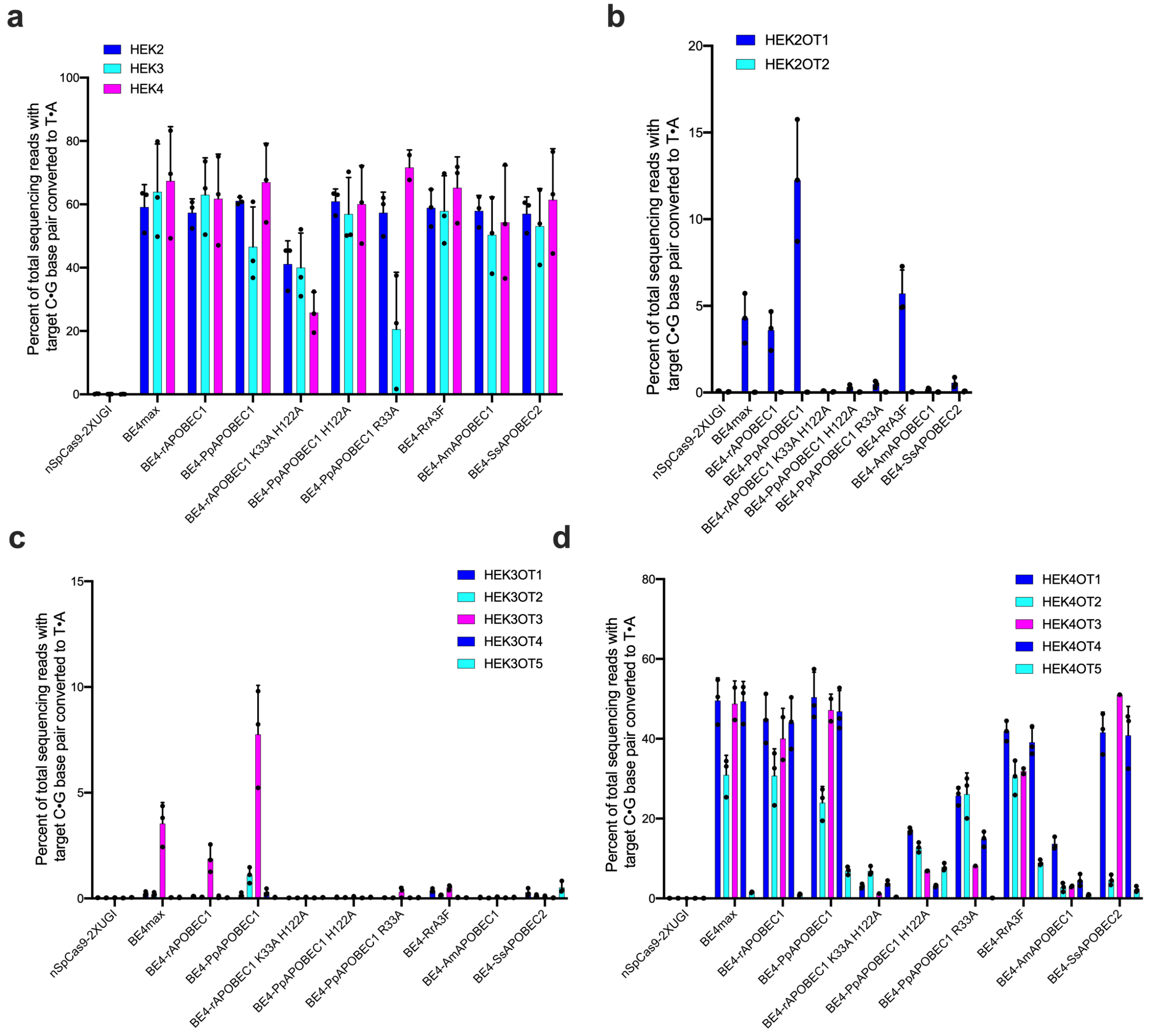

Supplementary Figure 14. Quantification of CBE protein concentration in HEK293T cells transfected with plasmids encoding base editors. Protein concentration of base editors was quantified by measuring the total Cas9 protein concentration and normalized to the amount of total protein in cell lysate. BE protein concentration was normalized to that of BE4-rAPOBEC1. Values and error bars reflect the mean and s.d. of n=2, 3, or 5 independent biological replicates, as can be determined for each sample by the number of included data in the graph. All data presented are provided as source data.

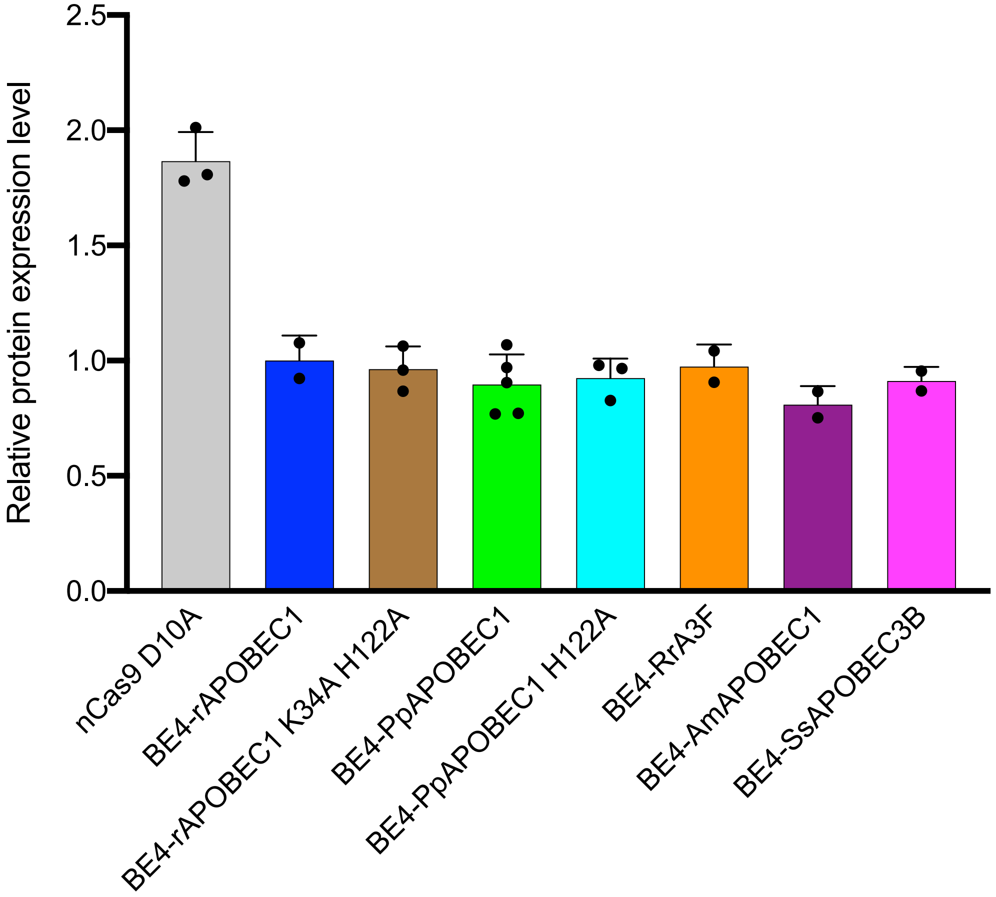

Supplementary Figure 15. Whole genome sequencing experiments to evaluate guide-independent cytidine deamination activity from base editors[1]. **a,** whole genome sequencing of single cell expansions to detect guide-independent cytidine deamination in HEK293T cells treated with base editor mRNA or plasmids. Editing efficiency from plasmids (**b**) or mRNA (**c**) transfection by amplicon sequencing of the target site. B2M guide sequence is ACTCACGCTGGATAGCCTCC. All data presented are provided as source data.

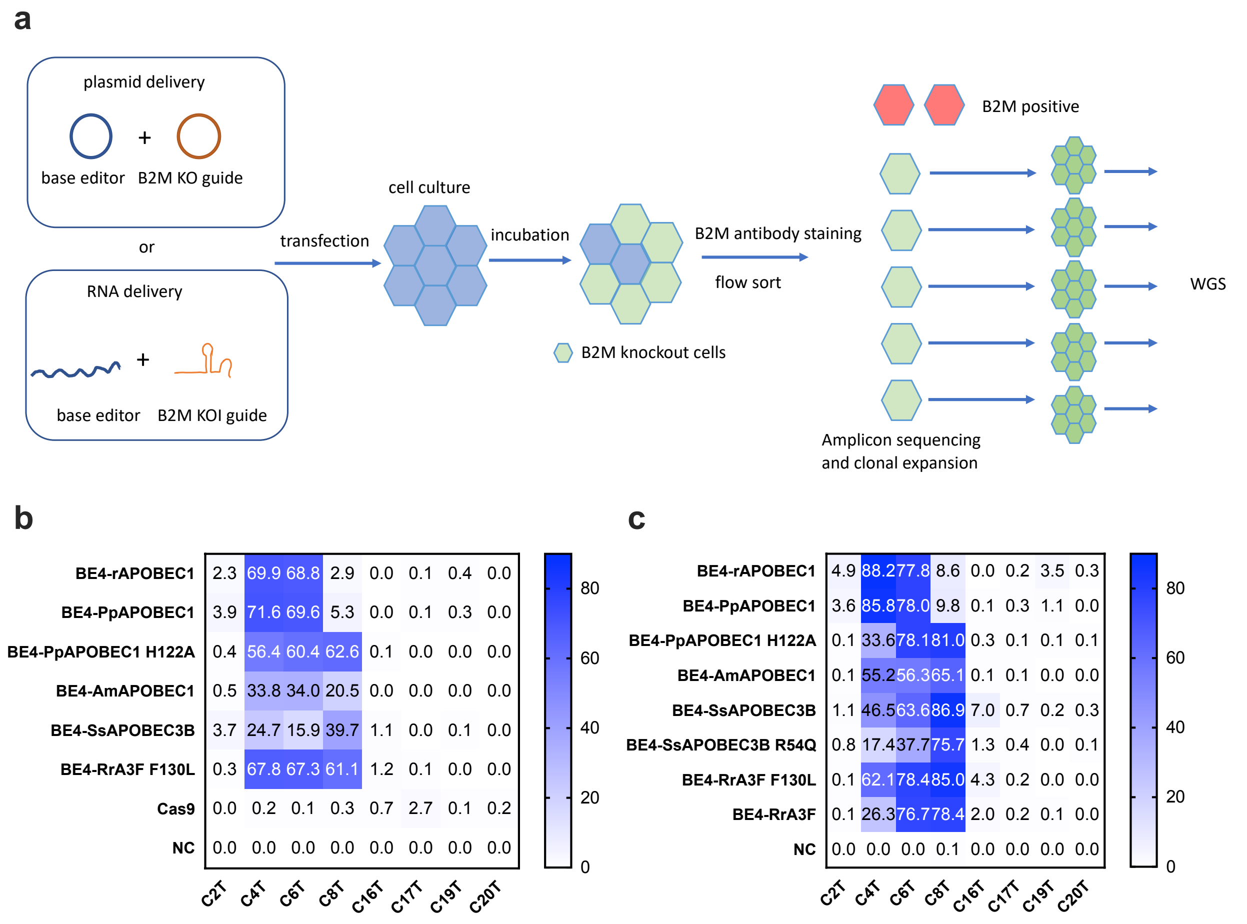

Supplementary Figure 16. C to U editing efficiency of selected CBEs on ssDNA substrates in *in vitro* enzymatic assay. The editing efficiencies were measured at 43 cytosines cytidines in ssDNA oligo 2-5 after 24 h incubation, and group by difference base prior to the target C. The sequences of these 4 substrates were listed in supplementary Table 3. Individual data points represent the mean value from n=2 independent biological replicates of Y=2 experiments. All data presented are provided as source data.

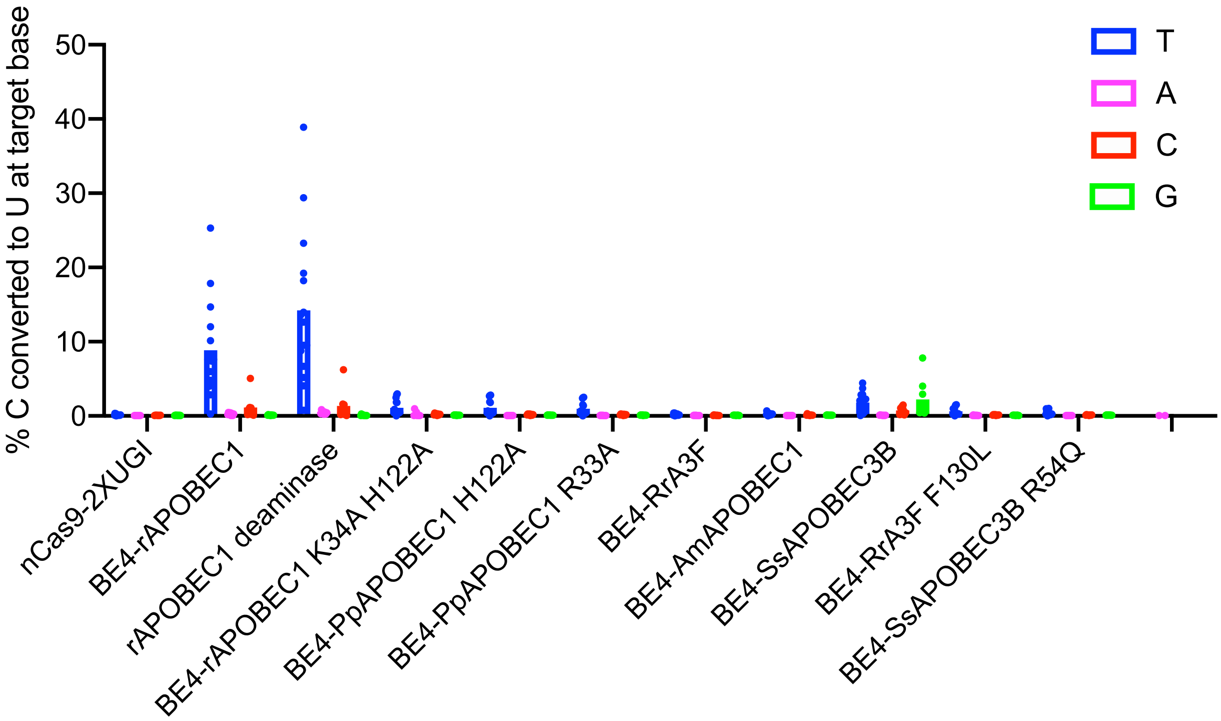

Supplementary Table 1. Amino acid sequences of all deaminases tested in this study.

Gene /construct name	Species/pfam ID	Amino acid sequences (5'-3")
rAPOBEC1	*Rattus norvegicus*	MSSETGPVAVDPTLRRRIEPHEFEVFFDPRELRKETCLLYEINWGGRHSIWRHTSQNTNKHVEVNFIEKFTTERYFCP<br>NTRCSITWFLSWSPCGECSRAITEFLSRYPHVTLFIYIARLYHHADPRNRQGLRDLISSGVTIQIMTEQESGYCWRNFV<br>NYSPSNEAHWPRYPHLWVRLYVLELYCIILGLPPCLNILRRKQPQLTFFTIALQSCHYQRLPPHILWATGLK
mAPOBEC1	*Mus musculus*	MSSETGPVAVDPTLRRRIEPHEFEVFFDPRELRKETCLLYEINWGGRHSVWRHTSQNTSNHVEVNFLEKFTTERYFRP<br>NTRCSITWFLSWSPCGECSRAITEFLSRHPYVTLFIYIARLYHHTDQRNRQGLRDLISSGVTIQIMTEQEYCYCWRNFV<br>NYPPSNEAYWPRYPHLWVKLYVLELYCIILGLPPCLKILRRKQPQLTFFTITLQTCHYQRIPPHLLWATGLK
MaAPOBEC1	*Mesocricetus auratus*	MSSETGPVVVDPTLRRRIEPHEFDAFFDQGELRKETCLLYEIRWGGRHNIWRHTGQNTSRHVEINFIEKFTSERYFYP<br>STRCSIVWFLSWSPCGECSKAITEFLSGHPNVTLFIYAARLYHHTDQRNRQGLRDLISRGVTIRIMTEQEYCYCWRNF<br>VNYPPSNEVYWPRYPNLWMRLYALELYCIHLGLPPCLKIKRRHQYPLTFFRLNLQSCHYQRIPPHILWATGFI
hAPOBEC1	*Homo sapiens*	MTSEKGPSTGDPTLRRRIEPWEFDVFYDPRELRKEACLLYEIKWGMSRKIWRSSGKNTTNHVEVNFIKKFTSERDFHP<br>SMSCSITWFLSWSPCWECSQAIREFLSRHPGVTLVIYVARLFWHMDQQNRQGLRDLVNSGVTIQIMRASEYYHCW<br>RNFVNYPPGDEAHWPQYPPLWMMLYALELHCIILSLPPCLKISRRWQNHLTFFRLHLQNCHYQTIPPHILLATGLIHP<br>SVAWR
PpAPOBEC1	*Pongo pygmaeus*	MTSEKGPSTGDPTLRRRIESWEFDVFYDPRELRKETCLLYEIKWGMSRKIWRSSGKNTTNHVEVNFIKKFTSERRFHS<br>SISCSITWFLSWSPCWECSQAIREFLSQHPGVTLVIYVARLFWHMDQRNRQGLRDLVNSGVTIQIMRASEYYHCWR<br>NFVNYPPGDEAHWPQYPPLWMMLYALELHCIILSLPPCLKISRRWQNHLAFFRLHLQNCHYQTIPPHILLATGLIHPS<br>VTWR
OcAPOBEC1	*Oryctolagus cuniculus*	MASEKGPSNKDYTLRRRIEPWEFEVFFDPQELRKEACLLYEIKWGASSKTWRSSGKNTTNHVEVNFLEKLTSEGRLG<br>PSTCCSITWFLSWSPCWECSMAIREFLSQHPGVTLIIFVARLFQHMDRRNRQGLKDLVTSGVTVRVMSVSEYCYCW<br>ENFVNYPPGKAAQWPRYPPRWMLMYALELYCIILGLPPCLKISRRHQKQLTFFSLTPQYCHYKMIPPYILLATGLLQPS<br>VPWR
MdAPOBEC1	*Monodelphis domestica*	MNSKTGPSVGDATLRRRIKPWEFVAFFNPQELRKETCLLYEIKWGNQNIWRHSNQNTSQHAEINFMEKFTAERHF<br>NSSVRCSITWFLSWSPCWECSKAIRKFLDHYPNVTLAIFISRLYWHMDQQHRQGLKELVHSGVTIQIMSYSEYHYCW<br>RNFVDYPQGEEDYWPKYPYLWIMLYVLELHCIILGLPPCLKISGSHSNQLALFSLDLQDCHYQKIPYNVLVATGLVQPF<br>VTWR
mAPOBEC2	*Mus musculus*	MAQKEEAAEAAAPASQNGDDLENLEDPEKLKELIDLPPFEIVTGVRLPVNFFKFQFRNVEYSSGRNKTFLCYVVEVQS<br>KGGQAQATQGYLEDEHAGAHAEEAFFNTILPAFDPALKYNVTWYVSSSPCAACADRILKTLSKTKNLRLLILVSRLFM<br>WEEPEVQAALKKLKEAGCKLRIMKPQDFEYIWQNFVEQEEGESKAFEPWEDIQENFLYYEEKLADILK
hAPOBEC2	*Homo sapiens*	MAQKEEAAVATEAASQNGEDLENLDDPEKLKELIELPPFEIVTGERLPANFFKFQFRNVEYSSGRNKTFLCYVVEAQG<br>KGGQVQASRGYLEDEHAAAHAEEAFFNTILPAFDPALRYNVTWYVSSSPCAACADRIIKTLSKTKNLRLLILVGRLFM<br>WEEPEIQAALKKLKEAGCKLRIMKPQDFEYVWQNFVEQEEGESKAFQPWEDIQENFLYYEEKLADILK
PpAPOBEC2	*Pongo pygmaeus*	MAQKEEAAAATEAASQNGEDLENLDDPEKLKELIELPPFEIVTGERLPANFFKFQFRNVEYSSGRNKTFLCYVVEAQG<br>KGGQVQASRGYLEDEHAAAHAEEAFFNTILPAFDPALRYNVTWYVSSSPCAACADRIIKTLSKTKNLRLLILVGRLFM<br>WEELEIQDALKKLKEAGCKLRIMKPQDFEYVWQNFVEQEEGESKAFQPWEDIQENFLYYEEKLADILK
BtAPOBEC2	*Bos taurus*	MAQKEEAAAAAEPASQNGEEVENLEDPEKLKELIELPPFEIVTGERLPAHYFKFQFRNVEYSSGRNKTFLCYVVEAQS<br>KGGQVQASRGYLEDEHATNHAEEAFFNSIMPTFDPALRYMVTWYVSSSPCAACADRIVKTLNKTKNLRLLILVGRLF<br>MWEEPEIQAALRKLKEAGCRLRIMKPQDFEYIWQNFVEQEEGESKAFEPWEDIQENFLYYEEKLADILK
mAPOBEC3	*Mus musculus*	MQPQRLGPRAGMGPFCLGCSHRKCYSPIRNLISQETFKFHFKNLGYAKGRKDTFLCYEVTRKDCDSPVSLHHGVFKN<br>KDNIHAEICFLYWFHDKVLKVLSPREEFKITWYMSWSPCFECAEQIVRFLATHHNLSLDIFSSRLYNVQDPETQQNLC<br>RLVQEGAQVAAMDLYEFKKCWKKFVDNGGRRFRPWKRLLTNFRYQDSKLQEILRPCYISVPSSSSSTLSNICLTKGLP<br>ETRFWVEGRRMDPLSEEEFYSQFYNQRVKHLCYYHRMKPYLCYQLEQFNGQAPLKGCLLSEKGKQHAEILFLDKIRS<br>MELSQVTITCYLTWSPCPNCAWQLAAFKRDRPDLILHIYTSRLYFHWKRPFQKGLCSLWQSGILVDVMDLPQFTDC<br>WTNFVNPKRPFWPWKGLEIISRRTQRRLRRIKESWGLQDLVNDFGNLQLGPPMS
hAPOBEC3A	*Homo sapiens*	MEASPASGPRHLMDPHIFTSNFNNGIGRHKTYLCYEVERLDNGTSVKMDQHRGFLHNQAKNLLCGFYGRHAELRF<br>LDLVPSLQLDPAQIYRVTWFISWSPCFSWGCAGEVRAFLQENTHVRLRIFAARIYDYDPLYKEALQMLRDAGAQVSI<br>MTYDEFKHCWDTFVDHQGCPFQPWDGLDEHSQALSGRLRAILQNQGN
hAPOBEC3B	*Homo sapiens*	MNPQIRNPMERMYRDTFYDNFENEPILYGRSYTWLCYEVKIKRGRSNLLWDTGVFRGQVYFKPQYHAEMCFLSWF<br>CGNQLPAYKCFQITWFVSWTPCPDCVAKLAEFLSEHPNVTLTISAARLYYYWERDYRRALCRLSQAGARVTIMDYEE<br>FAYCWENFVYNEGQQFMPWYKFDENYAFLHRTLKEILRYLMDPDTFTFNFNNDPLVLRRRQTYLCYEVERLDNGT<br>WVLMDQHMGFLCNEAKNLLCGFYGRHAELRFLDLVPSLQLDPAQIYRVTWFISWSPCFSWGCAGEVRAFLQENT<br>HVRLRIFAARIYDYDPLYKEALQMLRDAGAQVSIMTYDEFEYCWDTFVYRQGCPFQPWDGLEEHSQALSGRLRAIL<br>QNQGN
hAPOBEC3C	*Homo sapiens*	MNPQIRNPMKAMYPGTFYFQFKNLWEANDRNETWLCFTVEGIKRRSVVSWKTGVFRNQVDSETHCHAERCFLS<br>WFCDDILSPNTKYQVTWYTSWSPCPDCAGEVAEFLARHSNVNLTIFTARLYYFQYPCYQEGLRSLSQEGVAVEIMDY<br>EDFKYCWENFVYNDNEPFKPWKGLKTNFRLLKRRLRESLQ
hAPOBEC3D	*Homo sapiens*	MNPQIRNPMERMYRDTFYDNFENEPILYGRSYTWLCYEVKIKRGRSNLLWDTGVFRGPVLPKRQSNHRQEVYFRFE<br>NHAEMCFLSWFCGNRLPANRRFQITWFVSWNPCLPCVVKVTKFLAEHPNVTLTISAARLYYYRDRDWRWVLLRLH<br>KAGARVKIMDYEDFAYCWENFVCNEGQPFMPWYKFDDNYASLHRTLKEILRNPMEAMYPHIFYFHFKNLLKACGR<br>NESWLCFTMEVTKHHSAVFRKRGVFRNQVDPETHCHAERCFLSWFCDDILSPNTNYEVTWYTSWSPCPECAGEVA

		EFLARHSNVNLTIFTARLCYFWDTDYQEGLCSLSQEGASVKIMGYKDFVSCWKNFVYSDDEPFKPWKGLQTNFRLLKRRLREILQ
hAPOBEC3F	*Homo sapiens*	MKPHFRNTVERMYRDTFSYNFYNRPILSRRNTVWLCYEVKTKGPSRPRLDAKIFRGQVYSQPEHHAEMCFLSWFCGNQLPAYKCFQITWFVSWTPCPDCVAKLAEFLAEHPNVTLTISAARLYYYWERDYRRALCRLSQAGARVKIMDDEEFAYCWENFVYSEGQPFMPWYKFDDNYAFLHRTLKEILRNPMEAMYPHIFYFHFKNLRKAYGRNESWLCFTMEVVKHHSPVSWKRGVFRNQVDPETHCHAERCFLSWFCDDILSPNTNYEVTWYTSWSPCPECAGEVAEFLARHSNVNLTIFTARLYYFWDTDYQEGLRSLSQEGASVEIMGYKDFKYCWENFVYNDDEPFKPWKGLKYNFLFLDSKLQEILE
hAPOBEC3G	*Homo sapiens*	MKPHFRNTVERMYRDTFSYNFYNRPILSRRNTVWLCYEVKTKGPSRPPLDAKIFRGQVYSELKYHPEMRFFHWFSKWRKLHRDQEYEVTWYISWSPCTKCTRDMATFLAEDPKVTLTIFVARLYYFWDPDYQEALRSLCQKRDGPRATMKIMNYDEFQHCWSKFVYSQRELFEPWNNLPKYYILLHIMLGEILRHSMDPPTFTFNFNNEPWVRGRHETYLCYEVERMHNDTWVLLNQRRGFLCNQAPHKHGFLEGRHAELCFLDVIPFWKLDLDQDYRVTCFTSWSPCFSCAQEMAKFISKNKHVSLCIFTARIYDDQGRCQEGLRTLAEAGAKISIMTYSEFKHCWDTFVDHQGCPFQPWDGLDEHSQDLSGRLRAILQNQEN
hAPOBEC4	*Homo sapiens*	MEPIYEEYLANHGTIVKPYYWLSFSLDCSNCPYHIRTGEEARVSLTEFCQIFGFPYGTTFPQTKHLTFYELKTSSGSLVQKGHASSCTGNYIHPESMLFEMNGYLDSAIYNNDSIRHIILYSNNSPCNEANHCCISKMYNFLITYPGITLSIYFSQLYHTEMDFPASAWNREALRSLASLWPRVVLSPISGGIWHSVLHSFISGVSGSHVFQPILTGRALADRHNAYEINAITGVKPYFTDVLLQTKRNPNTKAQEALESYPLNNAFPGQFFQMPSGQLQPNLPPDLRAPVVFVLVPLRDLPPMHMGQNPNKPRNIVRHLNMPQMSFQETKDLGRLPTGRSVEIVEITEQFASSKEADEKKKKKGKK
mAPOBEC4	*Mus musculus*	MDSLLMKQKKFLYHFKNVRWAKGRHETYLCYVVKRRDSATSCSLDFGHLRNKSGCHVELLFLRYISDWDLDPGRCYRVTWFTSWSPCYDCARHVAEFLRWNPNLSLRIFTARLYFCEDRKAEPEGLRRLHRAGVQIGIMTFKDYFYCWNTFVENRERTFKAWEGLHENSVRLTRQLRRILLPLYEVDDLRDAFRMLGF
rAPOBEC4	*Rattus norvegicus*	MEPLYEEYLTHSGTIVKPYYWLSVSLNCTNCPYHIRTGEEARVPYTEFHQTFGFPWSTYPQTKHLTFYELRSSSGNLIQKGLASNCTGSHTHPESMLFERDGYLDSLIFHDSNIRHIILYSNNSPCDEANHCCISKMYNFLMNYPEVTLSVFFSQLYHTENQFPTSAWNREALRGLASLWPQVTLSAISGGIWQSILETFVSGISEGLTAVRPFTAGRTLTDRYNAYEINCITEVKPYFTDALHSWQKENQDQKVWAASENQPLHNTTPAQWQPDMSQDCRTPAVFMLVPYRDLPPIHVNPSPQKPRTVVRHLNTLQLSASKVKALRKSPSGRPVKKEEARKGSTRSQEANETNKSKWKKQTLFIKSNICHLLEREQKKIGILSSWSV
MfAPOBEC4	*Macaca fascicularis*	MEPTYEEYLANHGTIVKPYYWLSFSLDCSNCPYHIRTGEEARVSLTEFCQIFGFPYGTTYPQTKHLTFYELKTSSGSLVQKGHASSCTGNYIHPESMLFEMNGYLDSAIYNNDSIRHIILYCNNSPCNEANHCCISKVYNFLITYPGITLSIYFSQLYHTEMDFPASAWNREALRSLASLWPRVVLSPISGGIWHSVLHSFVSGVSGSHVFQPILTGRALTDRYNAYEINAITGVKPFFTDVLLHTKRNPNTKAQMALESYPLNNAFPGQSFQMTSGIPPDLRAPVVFVLLPLRDLPPMHMGQDPNKPRNIIRHLNMPQMSFQETKDLERLPTRRSVETVEITERFASSKQAEEKTKKKGKK
hAID	*Homo sapiens*	MDSLLMNRRKFLYQFKNVRWAKGRRETYLCYVVKRRDSATSFSLDFGYLRNKNGCHVELLFLRYISDWDLDPGRCYRVTWFTSWSPCYDCARHVADFLRGNPNLSLRIFTARLYFCEDRKAEPEGLRRLHRAGVQIAIMTFKDYFYCWNTFVENHERTFKAWEGLHENSVRLSRQLRRILLPLYEVDDLRDAFRTLGL
BtAID	*Bos taurus*	MDSLLKKQRQFLYQFKNVRWAKGRHETYLCYVVKRRDSPTSFSLDFGHLRNKAGCHVELLFLRYISDWDLDPGRCYRVTWFTSWSPCYDCARHVADFLRGYPNLSLRIFTARLYFCDKERKAEPEGLRRLHRAGVQIAIMTFKDYFYCWNTFVENHERTFKAWEGLHENSVRLSRQLRRILLPLYEVDDLRDAFRTLGL
mAID	*Mus musculus*	MDSLLMNRRKFLYQFKNVRWAKGRRETYLCYVVKRRDSATSFSLDFGYLRNKNGCHVELLFLRYISDWDLDPGRCYRVTWFTSWSPCYDCARHVADFLRGNPNLSLRIFTARLYFCEDRKAEPEGLRRLHRAGVQIAIMTFKDYFYCWNTFVENHERTFKAWEGLHENSVRLSRQLRRILLPLYEVDDLRDAFRTLGL
PmCDA1	*Petromyzon marinus*	MAGYECVRVSEKLDFDTFEFQFENLHYATERHRTYVIFDVKPQSAGGRSRRLWGYIINNPNVCHAELILMSMIDRHLESNPGVYAMTWYMSWSPCANCSSKLNPWLKNLLEEQGHTLTMHFSRIYDRDREGDHRGLRGLKHVSNSFRMGVVGRAEVKECLAEYVEASRRTLTWLDTTESMAAKMRRKLFCILVRCAGMRESGIPLHLFTLQTPLLSGRVVWWRV
PmCDA2	*Petromyzon marinus*	MELREVVDCALASCVRHEPLSRVAFLRCFAAPSQKPRGTVILFYVEGAGRGVTGGHAVNYNKQGTSIHAEVLLLSAVRAALLRRRRCEDGEEATRGCTLHCYSTYSPCRDCVEYIQEFGASTGVRVVIHCCRLYELDVNRRRSEAEGVLRSLSRLGRDFRLMGPRDAIALLLGGRLANTADGESGASGNAWVTETNVVEPLVDMTGFGDEDLHAQVQRNKQIREAYANYASAVSLMLGELHVDPDKFPFLAEFLAQTSVEPSGTPRETRGRPRGASSRGPEIGRQRPADFERALGAYGLFLHPRIVSREADREEIKRDLIVVMRKHNYQGP
PmCDA5	*Petromyzon marinus*	MAGDENVRVSEKLDFDTFEFQFENLHYATERHRTYVIFDVKPQSAGGRSRRLWGYIINNPNVCHAELILMSMIDRHLESNPGVYAMTWYMSWSPCANCSSKLNPWLKNLLEEQGHTLMMHFSRIYDRDREGDHRGLRGLKHVSNSFRMGVVGRAEVKECLAEYVEASRRTLTWLDTTESMAAKMRRKLFCILVRCAGMRESGMPLHLFT
yCD	*Saccharomyces cerevisiae*	MVTGGMASKWDQKGMDIAYEEAALGYKEGGVPIGGCLINNKDGSVLGRGHNMRFQKGSATLHGEISTLENCGRLEGKVYKDTTLYTTLSPCDMCTGAIIMYGIPRCVVGENVNFKSKGEKYLQTRGHEVVVVDDERCKKIMKQFIDERPQDWFEDIGE
F7B644	tr|F7B644|F7B644_HORSE	MPRGRARERQRRNPMEKLDAEAFSFHFLNMEFVYDRNCSYLCYQVEGRLSGSPVLSEQGVFPNEVCGKTRRHAELCFLDWFRGRLSPDEYYCVTWFISWSPCSNCAREVAEFLKRHRNVELSIFAARLYYCRDHEQGLQSLCNRGAQLAVMLRKDFTYCWDNFVHNSGREFSPWENIDANSDLLARKLEDLLKNPMEKLHRKTFSFHFRNLKFAKGRKCSYLCYRVEGRLSGSPGLSEQGVFLNEVCDENCRHAELCFLHWFRGRLSPHADYRVTWFISWSPCSNCAREVAEFLKQHRNVELHISAARLYYWQRNKPGLRNLRSSGAQLAIMFFWDFRDCWDNFVHNSGRHFIPWKKINVNSRLLATKLEDLLKNPLEKLHPNTFSFHFCNLEFAYDRKYSYLCYQVEGRLSGSPGLSEQGVFLNEVCGKTRCHAELCFLDWFRVRLSPDEYYRVTWFISWSPCFYCAREVADFLKQYRNVKLSIFAARLYYCRDHAQGLRSLCSSGAQLAIMFFWDFRYCWDNFVHNSGREFRPWKKINVNSRLLATKLEDILK
D1LZA1	tr|D1LZA1|D1LZA1_PANTI	MEPWRPSPRNPMDRIDPKTFRFQFPNLRYASGRKLCYLCFQVERDYFYYNDSDWGVFRNEVHPWAPCHAEQCFLSWFRDQYPYRDEDYNVTWFLSWSPCPTCAEEVVEFLEEYRNLTLSIFTSRLYYFWHPNYQEGLCKLWDAGVQLDIMSCDEFEYCWDNFVYHKGMRFQRRNLLKDYDFLAAKLQEILSPGQQRKRDWPFPPRPGAQVDPRSWVQEVTEPGI

<table>
<tr><td></td><td></td><td>NTRRHPLHLLVSFLLPRPTMNPLQEDIFYRQFGNQHRVPKPYYYRRKTYLCYQLKLPEGTLIDKDCLRNKKKRHAEICFIDKIKSLTRDTSQRFEIICYITWSPCPFCAEELVAFVKDNPHLSLRIFASRLYVHWRWKYQQGLRHLHASGIPVAVMSLPEFEDCWRNFVDHQDRLFQPWRNLDQYSESIKRRLGKILTPLNDLRNDFRNLKLE</td></tr>
<tr><td>A0A3Q0DM17</td><td>tr|A0A3Q0DM17|A0A3Q0DM17_TARSY</td><td>MPMKRMYSNIYFDHFNNQRLLSGQNAPWLCFKVERVENCMLVPLETGVFGNQVSGCCGKTERPVEPTSLTRSVLVSPNPGTELRAQQPSRKGHLGKLGCVEYPSPGLALVMLGYGASTYCPDSSMYCPETCHHPEMCFLYWFEKTLSHEEQYQITWYVSWSPCVNCAEEVAEFLSVHPKVNLTIYAARLYCYQKLNHRQGLRRLCKEGACVKIMNYEEFDHCWENFVYNNYKSFKPWVKLQDNYELLATELDKILRIPMERMPQKKFRFHFQNLIAKDRNTTWLCFEVKNVRKKHPPDLLERGIFQNQVTPRINCHAEMCFLSWFLENMLLHGKRYQVTWYISWSPCSICAEEVAEFLSAHPKVSLTIYAARLYYFWVPGYRQGLRRLVEEGARVEIMNYEEFDYCWENFVSINNEPFQPWEGLHEKYGYLVTKLNNILG</td></tr>
<tr><td>A0A3Q0DNJ5</td><td>tr|A0A3Q0DNJ5|A0A3Q0DNJ5_TARSY</td><td>MEDNPEPRPRQQMDQDTFIFNFNNDPSVRGRHQTFLCYEVEHLDDDTWVPQDKYLGFLHNQPQSRSNAYCAYHAELCFLELVSSWQLDPAQRYRVTCFISWSPCSSCAQEVAAFLKKNRHVTLRILAARIYDYYQGYEDGLRTLQGVGVDITVMTSAEFGHCWNTFVDHQGSPFQPWEGLDQHSQVIWQRMQDILQVIPAKYLMEKVKYTVTVDILFKGRVPGPRYLMDQNTFTRNFINNLSVSGRRQTLLCYEVERLGGDIWVPLDQLRGFLLSQARDVLNYYQGRHAEPCFLDLVSSWQLDPAQHYRVTWFISWSPCTSCAQAVAAFLRENRHVTLRILAARIYDYHQGYEEGLRTLQRTGAHIDIMTFKEFGHCWNTFVNHKGSPFKSWTGLDQHSQALRKRLQDILHTMASSLWDQSEPKKPIPSQEVTLPESIPPSHGNRFRLVKRPS</td></tr>
<tr><td>G5AYU5</td><td>tr|G5AYU5|G5AYU5_HETGA</td><td>FCFLSCVHRKPIERIYKKAFRFYFRNLRCAYGRNKTFLCYEVKRERDNKVLHKGVVLNQVEPYMPLHAELRFLSWFHDTLLCPLGSYQVTLYVSWSPCSECAEELTTFLAGHRNVTMTIYVAQLYYCNWKSPNREGLKILIAEDARLRVMFYDEFLYCWRNFVKNDYNNFDPWSLLDENSRYHNRILQNILKGWGRPHRVGPEGEQTATPGGSGGHCISVFSLLRRREMTLKEETFRVQFNNAYKAPKPYRRRVTYLCYQLQEANGDPLTKGCLRTKKGYHAESRFIKRICSMDLGQDQSYQVTCFLTWSPCPHCAQELVSFKRAHPHLRLQIFTARLFFHWKRSYQEGLQRLCRAQVPVAVMGHPEFAYCWDNFVDHQPGPFEPPWAKLEYYSSCLKRRLQQILRSWGVDDLTNDFRNLQLGP</td></tr>
<tr><td>A0A2Y9QMV5</td><td>tr|A0A2Y9QMV5|A0A2Y9QMV5_TRIMA</td><td>MLSSPQTPGTRKPMKTLAPDEFSFNFENLRLAHGRNTTFLCFQVETKAPPSLNSPDSGIFQNQDHCPSHHHAEMVFLTWFQKRLSPAQHYEVTWYMSWSPCSRCAVQVAKFLKSNSTVNLSIFVARLYYPRELETKDGLHSLWQAGAQVQIMFFQDFKYCWENFVNNEGKPFQPWKNLDENSKDWDTELKDIHRNTTDLLTEEMFYSQFYNREKKSSIPRKTYLCYQLNEPQPVKRCLHYKKGYHAVTRFIDGIVSMNLDPARSYDITCYFTWSPCNRYARKLVSFIEDYPNLRLKVYTSRLYFHWCWTNMQGLQHLQNSRVTVAVMTFRDFEYCWKNFVDNQGKPFEPWEKLDLYSQSTERRLRRILKPLTPDVLNEDFGNLHL</td></tr>
<tr><td>H0XHI0</td><td>tr|H0XHI0|H0XHI0_OTOGA</td><td>LSCAFRDPMNRMYPKTFCQNFEKEPCPSNQNSSWLCFEVETKNSAVFFHRGVFRNQPAPPPRAPTSVLLSQGPVKTPCHAEECFLTWIQGVLPPDHHYHVTWYVSRGPCANCANLIVHFLAMHRRVTLTIFAAHLNFFWESDFQQGLLRMDQEGVQLHIMGYEEFEYCWDNFVYNQRKQFVPWNGLNENYEFMVSTLEDILRSPLDRIRQKDFSIHFRNSLWLDDKSTWLCFEVKRTKSPVPLYRGVFRNQSPPKTPCHAEVRFFTWLQDLPPDFCCQFTWYLSWSPCADCADLVANFLAKHRNVSLTIFVARLYYYRDPEMHRGLRRMYQEGANVDIMSVIEFEYCWDNFVYNQGKQFVPWNGLNENYEFLVPRLQEILE</td></tr>
<tr><td>A0A3M0K4Y7</td><td>tr|A0A3M0K4Y7|A0A3M0K4Y7_HIRRU</td><td>MYISKKALRRHFDPRVYPRETYLLCELQWEGSRRVWIHWIRNVPDHHAEEYFLEEVFEPRNYGFCNITLYLSWSPCCTCCSKIRDFLKRNPNVKIDIRVARLIYPDYAETRSSLRELNGLQRVSIQVMEAAGLSCIESKNHRISQVERDPKGSSSPTLFTLQDHLKLSNMTESVIQDSVSIQICYQMRILGFQCHIRWKLQPEDFQRNYSPNQIGRVVYLLYEVRWRRGSIWRNWCSNNPEQHAEVNFLENHFHHRPQTPCSITWFLSTSPCGKCSRRILEFLKSQPNVTLEIYAAKLFRHHDIRNRQGLRNLMMNGVTIYIMNLEGNPASLCLSVD</td></tr>
<tr><td>A0A3P4LUZ8</td><td>tr|A0A3P4LUZ8|A0A3P4LUZ8_GULGU</td><td>MSFEDYEYCWETFVDHKGMYFQSWDLLRDNDLLAAELKNILRSTMNPLRQEIFYHQFGNQPRAPRPYHRRKTYLCYQLQPHEGPITARVCLQNKKKRHAEIRFIDNIRALRLDRSQTFEITCYLTWSPCPTCAKALAVFVQDHPHISLRLFASRLFIHWCWKYQEGLRLLHRSRIPVAVMRLQEFEDCWRNFVDNQDEPFQPWNKLEQYSESITRRLRRILGHPQNNLENDFRNLHI</td></tr>
<tr><td>G5BPM8</td><td>tr|G5BPM8|G5BPM8_HETGA</td><td>RRRIEPWQFEASFDPRQLRRETCLLSEVRWGTSPRAWRGCSLNTARHAEVSFMDRLTSEGRLRGPVRCSITWFLSWSPCGACAQAIGEFLRQHPNVSLVIYIARLFWHVDEQNRQGLRDLVTRGVRMQVMSDPEFAHCWRNFVNYSPGQEARWPQVPPVWTWLYSLELHCILLNLPPCLKISRRHHNQLTFFQLILQNCHYQAIPSPVLLASGLIHPFVTW</td></tr>
<tr><td>H2M862</td><td>tr|H2M862|H2M862_ORYLA</td><td>MITKLDSVLLPKKKFIYHYKNMRWARGRHETYLCFVVKRRVGPESLSFDFGHLRNRNGCHVELLFLRHLSALCPGLWGYGATGQGRVSYSITWFCSWSPCANCSFRLAQFLSQTPNLRLRIFVSRLYFCDLEDSREREGLRMLKKVGVHITVMSYKDYFYCWQTFVARKQSKFKPWDGLHQNSVRLSRKLNRILQPCETEDFRDAFKLLGL</td></tr>
<tr><td>H0Y0C6</td><td>tr|H0Y0C6|H0Y0C6_OTOGA</td><td>MYLKTFYRHFNNRPYLSRRNDTWLCFEVKTTSSNSPGSFYSGVFRNQGPRYCPWHTELCFLTWVRPIVSHHHFYQITWYMSWSPCANCAWQVATFLATHENVSLTNYTVRIYYFWRQDYRQGLLRMIEEGTQVYVMSSKEFQHCWENFVDHWGTRWVTCWNRLKKNYEFLVTRLSEILSDPKERISPNTFYNQFNNTPVPRGRKDTWLCFEVKEKNSNSPGSFHRGVFQNQVFSGTSSHARRCPPDHHYEVTWYTSWSPCAHCAWHVVNFLTSNPNVSLTIFAARLYYIYRPEIQQGLRRVFQEGAKVHIMSLKEFKYCWAKLVYNSGMRFMPWYQFNFNFLFPNTTLKGDLH</td></tr>
<tr><td>A0A3Q2Z5X6</td><td>tr|A0A3Q2Z5X6|A0A3Q2Z5X6_HIPCM</td><td>MDVHFMNFIYHYKNMRWAKGRNETYLCFVVKRRVGPNSLTFDFGHLRNRNGCHVELLFLRYLGRRLSYSITWFCSWSPCANCSAALSQFLSRMPNLRLRIFVARLYFCDMEDSHEREGLRLLQKAGVQVTVMSYKDYYYCWQTFVDRKKSHFKAWEDLHQNSVRLSRKLNRILQPCEMDLRDAFKLLGL</td></tr>
<tr><td>A0A2K6NVA7 (RrA3F)</td><td>tr|A0A2K6NVA7|A0A2K6NVA7_RHIRO</td><td>MKPQIRDHRPNPMEAMYPHIFYFHFENLEKAYGRNETWLCFTVEIIKQYLPVPWKKGVFRNQVDPETHCHAEKCFLSWFCNNTLSPKKNYQVTWYTSWSPCPECAGEVAEFLAEHSNVKLTIYTARLYYFWDTDYQEGLRSLSEEGASVEIMDYEDFQYCWENFVYDDGEPFKRWKGLKYNFQSLTRRLREILQ</td></tr>
<tr><td>A0A2K6NY90</td><td>tr|A0A2K6NY90|A0A2K6NY90_RHIRO</td><td>MNPHIRNPMEAMYPGTFYFHFKNLWEADNRNESWLCFAVEVIKHHSTVSWKRGVFRNQVDPETHCHAEKCFLSWFCDNTLSPKKNYQVTWYTSWSPCPECAREVAKFLARHSNVMLTIYTARLYYSQYPNYQEGLRRLNEEGVPVEIMDYEDFKYCWENFVYNGDELFKPWKGLKYNFLFLDSKLQEILE</td></tr>
<tr><td>Q6ICH2</td><td>tr|Q6ICH2|Q6ICH2_HUMAN</td><td>MNPQIRNPMERMYRDTFYDNFENEPILYGRSYTWLCYEVKIKRGRSNLLWDTGVFRGPVLPKRQSNHRQEVDPETHCHAERCFLSWFCDDILSPNTNYEVTWYTSWSPCPECAGEVAEFLARHSNVNLTIFTARLCYFWDTDYQEGLCSLSQEGASVKIMGYKDFVSCWKNFVYSDDEPFKPWKGLQTNFRLLKRRLREILQ</td></tr>
</table>

G8GPV1	tr|G8GPV1|G8GPV1_CERNE	MDGSPASRPGHVMDPGTFTSNFNNKPWVSGQRETYLCYKVERSHNDTWVLLNQHRGFLRNQAKNRLHGDYGCHAELCFLGEVPSWRLDPTQTYRVTWFISWSPCFSGGCAEQVRAFLQENTHVRLRIFAARIYDYDFLYQEALRTLRDAGAQVSIMTYEEFKHCWDTFVDHQGRPFQPWDGLDEHSQALSGRLQAILQNQGN
Q1WBT6	tr|Q1WBT6|Q1WBT6_SYMSY	MALLTAKTFRLQFNNKRRVTKPYYPRKALLCYQLTPQNGSTPTRGYFKNKKKRHAEIRFINKIKSMGLDETQCYQVTCYLTWSPCPSCAWELVDFIKAHDHLNLGIFASRLYYHWCRHQQEGLRLLCGSQVPVEVMGFPEFADCWENFVDHEEPLSFNPSEMLEELDKNSRAIKRRLEKIK
A0A3B4CS14	tr|A0A3B4CS14|A0A3B4CS14_PYGNA	MDNTNRRKFIYHYKNVRWARGRHETYLCFVVKKRNSPDSLSFDFGHLRNRNGCHVELLFLRYIEVLCPGLWGSGVDGVRVSYAVTWFCSWSPCSNCAQRLTNFLSQTPNLRLRIFVARLYFCDEEDSLEREGLRHLQRAGVQITVMTYKDFFYCWQTFVASRERCFKAWEGLRQNSVRLSRKLNRILQVFISTPVISPLITTHLGQSWAGG
A0A087XZI4	tr|A0A087XZI4|A0A087XZI4_POEFO	RKVSYSVTWFCSWSPCANCSIRLAQFLHQTPNLRLRIFVSRLYFCDLEDSREREGLRILKKAGVHITVMSYKDYFYCWQTFVAKSQSKFKPWDGLHQNYIRLSRKLNRILQPALDIKKFIYHYKNLRWARGRCETYLCFVVKKKLHLFMFVIVGRNRLFDLNVTMNNKSLYLIPLHLQLLFLRHLGALCPGLWGYGVTGERKVSYSVTWFCSWSPCANCSIRLAQFLHQTPNLRLRIFVSRLYFCDLEDSREREGLRILKKAGVHITVMSYKDYFYCWQTFVAKSQSKFKPWDGLHQNYIRLSRKLNRILQVQFF
A0A341AEK4	tr|A0A341AEK4|A0A341AEK4_9CETA	MASDRGPSAGDATSRRRIEPWEFEVSFDPRELCKETRLLYEIKWGRSQHVWRHSGKNTTNHVECNFIEKFTSERPFHRSVSCCITWFLSWSPCWECSKAIREFLNQHPRVTLFIYVARLFQHMDPQNRQGLRDLIHSGVTIQIMGPTEYDYCWRNFVNYPPGKEAHWPRYPPPLMKLYALELHCIILVP
E2D879	tr|E2D879|E2D879_MUSMI	RNLISRETFNFNFENLCYAKGRKNTFLCYEVTRKDCDSPVSLCHGVFKNKGSIHAEICFLYWFHDKVLKVLTPREEFKVTWYMSWSPCFECAEQVVRFLATHHNLNLTIFSSRLYNVSDPDTQQKLCRLVQEGAQVAVMDLSEFKKCWEKFVDNDGQQFRPWKRLRTNFRYQNSKLQEIL
A0A2K5RDN6	tr|A0A2K5RDN6|A0A2K5RDN6_CEBCA	MWEAQSPGLSREWGSVAISPEDPGPLHIGRFLSCAFRHPMNAMYPGIFNFHFRNLRKAYGRNETWLCFTVEGIMNRSTVSWKSGVFRNQVGSDPFCHAEMCFLSWFRHNMLSPKKDYEVTWYASWSPCPECAGQVAEFLARHGNVRLTIFTAHLYYFWNPSFRQGLRRLSQEGASVLIMGYEDFEYCWDNFVYNDGQPFKPWKRLQDNSLSLYITLQEILQ
A0A2K5RDN7	tr|A0A2K5RDN7|A0A2K5RDN7_CEBCA	MEASPASRPRPLMGPRTFTENFTNNPEVFGRHQTYLCYEVKCQGPDGTRDLMTEQRDFLCNQARNLLSGFDGRHAERCFLDRVPSWRLDPAQTYRVTCFISWSPCFSCAREVAEFLQENPHVNLRIFAARIYDCRPRYEEGLQMLQNAGAQVSIMTSEEFRHCWDTFVDHQGHPFQPWEGLDEHSQALSRRLQAILQGNRWMILSL
A0A1C9CJ69	tr|A0A1C9CJ69|A0A1C9CJ69_CERAL	NPMKAMDPHIFYFHFKNLRKAYGRNETWLCFAVEIIKQRSTVPWRTGVFRNQVDPESHCHAERCFLSWFCEDILSPNTDYRVTWYTSWSPCLDCAGEVAEFLARHSNVELAIFAARLYYFWDTHYQQGLRSLSEKGASVEIMGYEDFKYCRENFVCDDGKPFKPWKGLKTNFRFLKRRLQEILE
A0A2R2Z4D2	tr|A0A2R2Z4D2|A0A2R2Z4D2_PTEAL	MHLQVWRKVTEAWREGYTLKPWSRNPMERLYHDYFYFHFYNLPTPKHRNGCYICYQVEGTKKHSRMPLLRGVFENQESLDMMLSPGEKYRVTWYISWSPCFACVDEVIKFLREHTNVELIIFAARLYHSDILQYRQGLRKLHDAGVHVAIMSYYEFKHCLNDFVFHQGRSFCPWNDLNKNSKNLSNTLEDILRNQED
B7T161	tr|B7T161|B7T161_SHEEP	MTEGWAGSGLPGRGDCVWTPQTRNTMNLLRETLFKQQFGNQPRVPPPYYRRKTYLCYQLKELDDLMLDKGCFRNKKQRHAEIRFIDKINSLNLNPSQSYKIICYITWSPCPNCASELVDFITRNDHLNLQIFASRLYFHWIKPFCRGLHQLQKAGISVAVMTHTEFEDCWEQFVDNQLRPFQPWDKLEQYSASIRRRLQRILTAPT
A0A2R2X2G4	tr|A0A2R2X2G4|A0A2R2X2G4_PTEAL	MAGLGQACEGCCGQMPEISYPMGRLDPKTFSFEFKNLPYAYGRKSSYLCFQVEREQHSSPVPSDWGVFKNQFCGTEPYHAELCFLNWFRAEKLSPYEHYDVTWFLSWSPCSTCAEEIAIFLSNHKNVRLNIFVSRIYYFWKPAFRQGLQELDHLGVQLDAMSFDEFKYCWENFVDNQGMPFRCWKKVHQNYKSVLRKLNEILRRR
G1Q1M4	tr|G1Q1M4|G1Q1M4_MYOLU	YAELSFLDLFQSWNLDRGRQYRLTWYMSWSPYPDCAQKLVEFLGENSHVTLRIFAADIHSLCSGYEDGLRKLRDARAQLAIMTRDELQYCWVTFVDNQGQPFRPWPNLVEHIKTKKQELKDILGNPMRRMYPKTFNFNFQNLNSYGRKSTFLCFEVETWEDGSVLDYQNGVFQNQLDPGHAELCFIEWFHEKVLFPDEVRCPDAQYHVTWYISWSPCFECAEQVAGFLNEHENVDLSISAARLYLCEDEDEQGLQDLVAAGAKVAMMAPEDFEYCWDNFVYNRGWPFTYWKHVRRNYGRLQEKLDEILW
A0A1S3AN78	tr|A0A1S3AN78|A0A1S3AN78_ERIEU	RRIEPWEFEDFFDPRQFRPETCLLYEVRWGSSRNAWRSTARNTTRHAEVNFLERFAAERHFDKPVSCSITWFLSWSPCWECSQAIGAFLSQHPQVTLAIHVTRLFHHEDEQNRQGLRDLLARGVTLQVMGDSEYAHCWRTFVNSPPGAEGHYPRYPSDFTRLYALELHCIILGLPPCLEILRRYQNQFTLFRLVPQNCHYQMIPHLNFFVVRHYFF
A0A151P7C9 (AmAPOBEC1)	tr|A0A151P7C9|A0A151P7C9_ALLMI	MADSSEKMRGQYISRDTFEKNYKPIDGTKEAHLLCEIKWGKYGKPWLHWCQNQRMNIHAEDYFMNNIFKAKKHPVHCYVTWYLSWSPCADCASKIVKFLEERPYLKLTIYVAQLYYHTEEENRKGLRLLRSKKVIIRVMDISDYNYCWKVFVSNQNGNEDYWPLQFDPWVKENYSRLLDIFWESKCRSPNPW
Q4VUI3	tr|Q4VUI3|Q4VUI3_XENLA	MTMDSMLLKRNKFIYHYKNLRWARGRHETYLCYIVKRRYSSVSCALDFGYLRNRNGCHAEMLFLRYLSIWVGHDPHRNYRVTWFSSWSPCYDCAKRTLEFLKGHPNFSLRIFSARLYFCEERNAEPEGLRKLQKAGVRLSVMSYKDYFYCWNTFVETRESGFEAWDGLHENSVRLARKLRRILQPPYDMEDLREVFVLLGL
E2RL86	tr|E2RL86|E2RL86_CANLF	MNPLQEETFYQQFSNQRVPKPTYQRRTYLCYQLKPHEGSVIAKVCLQNQEKRHAEICFIDDIKSRQLDPSQKFEITCYVTWSPCPTCAKKLIAFVNDHPHISLRLFASRLYFHWRQKYKRELRHLQKSGIPLAVMSYLEFKDCWEKFVDHKGRPFQPWNKLKQYSESIGRRLQRILQPLNNLENDFRNLRL
G1LWB0	tr|G1LWB0|G1LWB0_AILME	SSAAPASIHLLDEDTFTENFRNDDWPSRTYLCYKVEGPDQGSGVPLGQDKGILHNKPAQGPEPSRHAECYLLEQIQSWNLDPKLHYGVTCFLSWSPCAKCAQKMARFLQENSHVSLKLFASRLYTRERWDEDYKEGLRTLKRAGASIAIMTYREFEHCWKTFVLHDQEGSCFQPWPFLHKESQKFSEKLQAILQVGVLLLSLPPPLPSSPLSSPWPFPAPLRASTG
A0A1U7S7K7	tr|A0A1U7S7K7|A0A1U7S7K7_ALLSI	MGEHWQYAGSGEYIPQDQFEENFDPSVLLAETHLLSELTWGGRPYKHWYENTEHCHAEIHFLENFSSKNRSCTITWYLSWSPCAECSARIADFMQENTNVKLNIHVARLYLHDDEHTRQGLRYLMKMKRVTIQVMTIPDYTYCWNTFLEDDGEDESDDYGGYAGVHEDEDESDDDDYLPTHFAPWIMLYSLELSCILQGFAPCLKIIQGNHMSPTFQLHVQDQEQKRLLEPANPWGAD
A0A2R2X2J8	tr|A0A2R2X2J8|A0A2R2X2J8_PTEVA	MPRIGNMNLLSEKTFNYHFGNQLRVKKPQGRRRTYLCYKLKLPNETLVKGYFINKKKNHAEIRFINKIRSLNLDQTQSYKITCYITWSPCSYCAGKLVALVKSCPHLSLQIFTSRLYYHWLWKNQAGLRYLWKINISVLVMKEPEFADCWDNFVNHQSRRFKPWEKLTQYSNSTERRLLRILRINRTDLFLAQSSEQDPGLNDLVDAIKRLFLDAHRPRD

<table>
<tr><td>A0A151P6M4</td><td>tr|A0A151P6M4|A0A151P6M4_ALLMI</td><td>MAVEEEKGLLGTSQGWKIELKDFQENYMPSTWPKVTHLLYEIRWGKGSKVWRNWCSNTLTQHAEVNCLENAFGKLQFNPPVPCHITWFLSWSPCCQCCRRILQFLRAHSHITLVIKAAQLFKHMDERNRQGLRDLVQSGVHVQVMDLPDYRYCWRTFVSHPHEGEGDFWPWFFPLWITFYTLELQHILLQQHALSYNL</td></tr>
<tr><td>A0A2K6MNR2</td><td>tr|A0A2K6MNR2|A0A2K6MNR2_RHIBE</td><td>IWLCFTMEIIKQCSTVSWKRGVFRNQVDPETHCHAERCFLSWFWEDTLSPNTNYQVTWYTSWSPCLDCAGEVAEFLARHSNVKLAIFAARLYYFWDTDYQQGLRSLSEEGTSVEIMGYEDFKYCWENFVYNGDEPFKPWKGLKYNFLFLDSKLQEILE</td></tr>
<tr><td>D3U1S2 (SsAPOBEC3B)</td><td>tr|D3U1S2|D3U1S2_PIG</td><td>MDPQRLRQWPGPGPASRGGYGQRPRIRNPEEWFHELSPRTFSFHFRNLRFASGRNRSYICCQVEGKNCFFQGIFQNQVPPDPPCHAELCFLSWFQSWGLSPDEHYYVTWFISWSPCCECAAKVAQFLEENRNVSLSLSAARLYYFWKSESREGLRRLSDLGAQVGIMSFQDFQHCWNNFVHNLGMPFQPWKKLHKNYQRLVTELKQILREEPATYGSPQAQGKVRIGSTAAGLRHSHSHTRSEAHLRPNHSSRQHRILNPPREARARTCVLVDASWICYR</td></tr>
<tr><td>F1CGT0</td><td>tr|F1CGT0|F1CGT0_ANOCA</td><td>KAAILLSNLFFRWQMEPEAFQRNFDPREFPECTLLLYEIHWDNNTSRNWCTNKPGLHAEENFLQIFNEKIDIKQDTPCSITWFLSWSPCYPCSQAIIKFLEAHPNVSLEIKAARLYMHQIDCNKEGLRNLGRNRVSIMNLPDYRHCWTTFVVPRGANEDYWPQDFLPAITNYSRELDSILQD</td></tr>
<tr><td>C7AGG3</td><td>tr|C7AGG3|C7AGG3_HORSE</td><td>MDPQAPTQRGGLGQAYQGGDYVQAPGNGNTQHLLSEDVFKKQFGNQRRVTKPYYRRKTYVCYQLKLLRGPTIAKGYFRNKKKRHAEIRFIDKINSLGLDQDQSYEITCYVTWSPCATCACKLIKFTRKFPNLSLRIFVSRLYYHWFRQNQQGLRQLWASSIPVVVMGYQEFADCWENFADNRGNPFQSWEKLTEYSKGIKRRLQKILEPLNLNGLEDAMGNLKLGSVDLG</td></tr>
<tr><td>A0A250YMK7</td><td>tr|A0A250YMK7|A0A250YMK7_CASCN</td><td>MSLLKEDIFLYQFNNQQQVQKPYFRRRTYLCYQLEQPNGSRPQWPAKGCLQNKKGHHAEIRFIKRIHSMGLEQDQDYQITCYITWSPCLACACALAELKNHFPRLTLRIFASRLYFHWIRKFQMGLQHLYKSGVLVAVMSLPEFTDCWEKFVNHRQVFFTPWDKLEEHSRSIQRRLRRILQSWDVDDLTDDFRNLRL</td></tr>
<tr><td>B7T160</td><td>tr|B7T160|B7T160_SHEEP</td><td>MPWISDHVARLDPETFYFQFHNLLYAYGRNCSYICYRVKTWKHRSPVSFDWGVFHNQVYAGTHCHSERRFLSWFCAKKLRPDECYHITWFMSWSPCMKCAELVAGFLGMYQNVTLSIFTARLYYFQKPQYRKGLLRLSDQGACVDIMSYQEFKYCWKKFVYSQRRPFRPWKKLKRNYQLLAAELEDILG</td></tr>
<tr><td>A0A182D0J1</td><td>tr|A0A182D0J1|A0A182D0J1_BLAVI</td><td>MTNPESPPQAPCDFNEDALLNREPLRGSPIKFVSPVDYPDLVFALAGPVGVDIDYIQQSISDCLKSFDYSTEFIRITEIMQDIKCSKTIDCTDMLKEYQSKIEYANELRRAYRAKDLLAALTISAISKLREQIKERDEATNKSNIQPSRRKLAWVVRQLKTPEEVRLLRAVYGKQFVLVSIYSSPQRREDFLISKIKIKSRGTIDNNTSSEGAQRLIERDSKEDNEYGQNLSGTFCLGDIFVDSNNKESAIVSIDRFLNAFFGSNEISPTRDEYGMYLAKTASLRSCDLSRQVGAAIFSKTGEIISLGSNEVPKAGGGTYWTGDNADSRDIRLGHDPNEINKVEIFAEIISRLLEDKLLSNDLLNKDAASIVTILLSKNEGKRYKDLRVMDIIEFGRIIHAEMSAICDAARNGRAIIGATLFCTTFPCHLCAKHIVASGIGRIVYLEPYPKSYAKKLHSDSIQVEDHSDSEKVSFEPFIGISPSRYRELFEGGRRKDPFGEALKWKNDPRKPVIDVVVPPHFEAEKLVIAQLGKLIVSGTG</td></tr>
<tr><td>A0A2D6EXD2</td><td>tr|A0A2D6EXD2|A0A2D6EXD2_9ARCH</td><td>MIIGLVGTIGAGKQTIIDYLQEKYGYNALSCSDVLREILKKQGKPVTRDNLREIGNKTREEGGNGAIAKILLEKLRNNWKANYIVDSLRHPDEVSVLRTSPLFHLVAVDADLRIRFERVKARKREEEPTTLPAFVERDQKEMFGTGNEQRIRETMELADELVLNNGTVEELKQRIDDLNLVSDERLRPSWDDYFMRLARLAAQRSNCMSRKVGAIITKDRRVIATGYNGTPRGVKNCNEGGCERCNSAVAKGTAISECLCLHGEENAIIEAGRVRSEGATIYTSFLPCLWCTKMIIQAGLKEVVFSEVYDLHEASIKLFETSGVLIRRLK</td></tr>
<tr><td>F7YVM7</td><td>tr|F7YVM7|F7YVM7_9THEM</td><td>MNEFKYMSLALKLAKKGKYTTSPNPMVGAVIVKDGKILATGYHKKAGQPHAEINALSKLNFQAQNCEMYVTLEPCSHYGRTPPCADAIIRSGIRKVVIATLDPNPLVNGKGVEKLKNAGIEVVCGVLEEKAKKLNEKFFKYITTKIPFVALKIAQTLDGKIALKNGESKWITSEKSREYVHKLRMEYDAVLTGIGTILKDDPQLNVRLKKVYKQPLRIILDSKLKIPLSAKVLEDPSKVIILTTALADKEKLEELRSKGVEVIITNEKNGIVDLESALKILGEKKITSVMVEAGPTLLTSFLKESLFDKIYLFIAPKIFGADSKSVFSELGLEDISKSQKFSLESVKKIGEDLLLELYPKQLKKLEE</td></tr>
<tr><td>A0A3M6UNF1</td><td>tr|A0A3M6UNF1|A0A3M6UNF1_9CNID</td><td>MEEKSELENELMRSTSPKPSVPNGSKGNECEQRETRITKENLYMVLALWMEEFPVVEQTSSAKRLNKVGVVFVLPTDRVLAADCSRDGVHGVARVMVNHCGKLEGCKVFVSRKPCSLCAKLLVQSKVSRVFYLPIEPESENKGEIARADNLFKNSSVGQSVFVPCVEQKVLDKLEDKLPKEIITPDDISECRDNLLKKCGWSAEWFARAQASLPWPCFEGKMKSQVDNDFKSLIKWIAVVKAPMDKGVAFPKVKLTSDSRVVPDCDADNFPDSKTAYHMMIFAKMLARQTDDPKTGVGAVIVRGKVPDIVSLGWNGFPSKALYGEFPRASDDDRALQKKFPYVIHAEQNALMVRNVKDLTDGILFVTKPPCDECAPMIKLSGVKTIVIGEKIEKSRGGELSYNLIKEYIKEGIMTCYQMEATKTKAKRLASDPETRKRLKSSCSNSNDV</td></tr>
<tr><td>A0A2G3K826</td><td>tr|A0A2G3K826|A0A2G3K826_9BURK</td><td>MTKIIDDVNTAAAAVLDQATAAANQTTFAVGGVMVNNQTGEVISAIHNNVIIPLSNNVSFTFDPTAHGERQLVYWYYANKEALKLPEPNQITVITSLDPCAMCTGALLTAGFNVGVVAIDTYAGINCAQNFQFATLPANLRTKAQKNFGYYASGAANFKPLTRSYVGGPSVAFKNGVVTPANLRDCGTVFTQSVDTVRNTSNSTGLAPSQMSNPAELPSNSAILQAYRAIYKKAFTIKIDNPRLPDAQILTELKAVLADAPNARNAVAFIDPFGNLVLCMADAFNTSPVHAAFMNVTQEYAKTRWDLMNKYAQASTTDNPALYLTHPKYGTFVYLYAPDPDDSITIMSLGAYGSTMEGPIPNMFPSNLQFYYPPRNGAQFSELVPVVNELPPFYTQNVNISLMQVPGVTQAPTK</td></tr>
<tr><td>K1ZCJ4</td><td>tr|K1ZCJ4|K1ZCJ4_9BACT</td><td>MSSRAKKNRSTNLKKSIGQKSIENKPTDQKKDQVLVAYVPVIHEGYRRFFRHFPAVKELWLISQELSHELRSLQKDIRALKASETKKLLQTWGQFQKIKLLTPSSLAILQKTTTQLVFPDEEISHHLVEKYFAQNRVLFASFFLRWDKKSSLKKHDLQEYSEISNKEFDQMMIAIAQQEADKSDDWWRQVGGLIFKDETILLLAHNQHTPTEAEAYFAGDPRADFHQGEYLKISTAIHAEAYLIAQAAKQGISLEGADLYVTTFPCPVCAKQVAYSGIKRVFFREGYSLLDGETILKANGVKLIRVTV</td></tr>
<tr><td>A0A1G3PNQ8</td><td>tr|A0A1G3PNQ8|A0A1G3PNQ8_9SPIR</td><td>MRDLPLLVLGLTGPMGAGCTRFARDISKMEPGKVIKKQGLLDQVAHEISELSKKASEIRLQCISNGKNSELAELKRLNRRLNAKLAERACLHVIAKSSLPEPLFISLNTIVIKIAVDSITAPEFAEWAKNHAKVADLLKWLRTQWESELTLYETWGQDAGRFSQDELEKMDAMFAEFERIGDEILKEDFETYFGKRNNDFSIRMFSENIRLSGNPFRPAENGGGGGKYDEPSMVMIARETDRYIRFYRTRSDQKRSHFFIIDEIKNPREAEYFRARHQNFFLVSIFSSSEIRASRMRRGLGHDAGVSDADFQHLFRELDSRDWGADDFDAHGLHRQNIYRCFNLADIAINNDVEDERFSEVLFNKFIRYYALMLSPGCVQPTPQETYMHLAYSLSLRSTCISRQVGAVITDLEDRILSLGWNEVPEGQIGCGLKVKKDYTDKENPLFEMEIWDNVITAEDLAVWDDEDSICVKDILSRIEIKTKLKSVSLTPEERADVLKALRIKRLEYSRSLHAEENAILQVASRGGVGLKDGTIYVTTFPCELCSKKIYQVGISKIYYTEPYPNSISEKVILKDGIRNIKILQFEGVKSYSYFKLFKPGFDKKDAQMLEGRGI</td></tr>
</table>

A0A1G0PGF4	tr|A0A1G0PGF4|A0A1G0PGF4_9BACT	MKHNNQLRKEIEKLLGQNSIIKNDELKKLQKEYKIETDELLISFLPYAAEFAKVPISKYKVGAVVLGKSGNIYFGSNMEFEAGALSATVHAEQSAVNNAWLNGETGINKIAVTAAPCGYCRQFLNELTTAKQLHVLLKDKNLEAAKVFKLTELLPEAFGPRDLEIEGGLMKVENHKLKIENINDELINAALEAANKSYAPYSKNYSGVSIQLSDGTIFSGRYSENAAYNPSLLPFQSALAFMNMNTKKGSNNKIVDAVLVEAVSNISQKDAAGTLLNSISKTKLRYYKIKN
A0A0P4WGY5	tr|A0A0P4WGY5|A0A0P4WGY5_9EUCA	MEENSSATSQPKCASRTKQGGNDLSTDMSNLSVGETKRTDFLPWDDYFMAVAFLSAMRSKDPSSQVGACIVNADKKIVGIGYNGMPIGCSDDELPWNKESLDPLQTKYMYVCHAEMNAIMNKNSSDLAGCCVYVALFPCNECAKLVIQAGIREVVFFSDKHQQKPETVASKKMLNMAGVAYRQYTPSQSKIELNLSLKEQEKSEPTADITQSSERDQNSKRKDYLSWEEYFMAMAHLSALRSKDPITQVGACIVNSKKKIVGIGYNGMPLGCNDDLMPWGNSSSNKLETKYMYVCHAGVNAIMNKNSCDVSGCTLYVALFPCNECAKVIIQAGIKTIIYASDTNKDQASILASKKMLDMAGIKYRADNLSQRKIVIDFKTIDWNSRFMNDHQNDPTCL
A0A3D8IG27	tr|A0A3D8IG27|A0A3D8IG27_9HELI	MRKNILYFILTLFFLSGLYATSLPEDNVVSGVIYEKIDTVSAEVDHIYPMLALAIVYKDWQEKNMLNKQGHNIGLVIVDENNMPVFWVRNSVHATHNGTQHGEVRLVSNLLNCEGFNKYLDKYTLYTTLEPCIMCAGMLSMVQIPKVVYAQKDLSCGNTQEIISTAKYPRYYKAFTVENGYKKDLEECFEQYKICKNDSITDFLVNDSAKEIFRKASNDLQDYKVKFKENRRVIKVAQEFLQNIQTKDNLDVLQCPKNM
A0A351C8C4	tr|A0A351C8C4|A0A351C8C4_9BACT	MNELTKQSEHLRNEALRIATRSYVPYTGQQEGVIILLENGDLIPGVRVENASFQLTIPALQNALSTMYALQRTDISMIVSSIPFTDSDLAYTGGMAEIAWEMVGASLLLVAGAHIPEAGTFIDPARGENLLDVSREAALNAFIPESDFPVGSAIQTSDDVVIDGCNVEHSDWSKIICAERNVLSTARSYGLGQITTIYVSCPKEPGGTPCGACRQVIVELAPDATVWMDRGNQEPIAMKATKLLPGHFTGNVLKKQ
A0A1G6V2K7	tr|A0A1G6V2K7|A0A1G6V2K7_PEPNI	MPIVRVNEIGARLPEDWEALETAIWQAYVSREDLPDAGELDLTLVDDATIQELNKTHRQLDKSTDVLSFPMYDDRDDLAADVQAGLPVILGDIMISVPTAERQAQAYGHSFKREMAYLLVHGLLHIAGYDHMSAEEKSAMRRAEEAILADVDVPRDTAPSKTAAVLDEADVQALIDAARAARLQAYAPYSGYAVGAALLAADGRRFCGVNVENASYGATCCAERTALFAAVTAGARDFIALALVTEGDEPAPPCGLCRQALAEFSPDLAIYLAGPTGETYRRTSLAALFPEAFSLSTKESV
F2NP91	tr|F2NP91|F2NP91_MARHT	MPVMETHALEARFKEALARLCPEGRLLAAVSGGGDSVALLYLLKAAGRDTIVAHLDHALRPDSAADAAFVEKLAQRLGFPLETEHVDVRALAHRKRINLEAAAREVRYAFLARVARRWKARCILTAHTLDDNAETVLLQILRGAGRGLGIRPLQRRVARPLLEFSRAELRAYLEARGARWLEDPTNRSLELDRNYLRHAVLPRITARFPHALEALARFSQAQQADDWALEALSARHLIPDRRWPVPAYRALPLERAPEALRRRAIRGVLEALGVRPEARLVADVEAALGGRAQTLPGGVVVRRQRGTLFFIPPTVRFPKVQPPAGLEARPPRPGDYLVFPYGRKRLVDFLNERGVPRELKRRWPVGAVGAEVRWVYGLWPEPDEDRYMRRALVLARAAARQGEVPIGAVLVRDGAVLAEAANAVEASRDATAHAELLALRTALRRVGEKVLPGATLYVTLEPCPMCYGAILEARVARVVYGVENLKAGAFTVHGLEPRVALEAGRVEGECAKVLKDFFARLRPGRDGA
A0A316TX77	tr|A0A316TX77|A0A316TX77_9BACT	MINGYTPYSGNQNTCYVKGESGTFYPGVRIENVSYPLTISSVQAAVCSCLANSDNPVEYYTGDHQPELLQVWADEYDMKPGGKLPDSPLKLFDPLVPSIPDIKKELDVLTEKSVTPNSGFPVSALLQTEKGYIRGVNIELSSWALGLCAERVAISRALTAGYTQFKSIHIYAPEADFVSPCGACRQVLLEVMPDADTELYHGDGTLSKHIVSDLLPFGFTSHKLKK
R6VYG3	tr|R6VYG3|R6VYG3_9FIRM	MIHKGTQTIETKRLILRAFTPDDAEAAFENWMSDPKVTEFLRWKTHADISDSRKIVNEWANGSADPEFYQWAIVPKDVNEPIGTISVVDRNDALGIFHIGYCIGSKWWHKGITSEAFSAVIHFLFEEVGANRIESQHDPENIHSGDVMKKCGLTFEGTLRQADFNNRGIVDACVYSILQSEWQNNTSVWQRLYNAALTVQNDRVVSPFIDAGGVAAALMTKKGNIYTGICIDTASTLGMCAERNAVANMLTNGESRIDKIVAVMPDGKVGAPCGACREYMMQLDRDSGDIEILLDLETEKTVRLKDLIPDWWGAERFGDTE
A0A3C1HZ18	tr|A0A3C1HZ18|A0A3C1HZ18_9BACI	MGDIMENWNELSEPWKRCFLQAWKAYCHGSIPIGAVLVDSEGEIFLEGRNRVHELTAPEGQLCDCRIAHAEMNVLVQVKTSDYEKLSGATIYSTMEPCIQCFGAIILSRIKNISFAAIDDKLAGATTLEDRHGFIKSRNLNIAGPFSHLGEIQIILRTDFLLRIFDSEYADPLIAAHEKDYPIGVALGRHYHRNNRLQVAKKETIPFGELFNEFSFDIKRAREGYTLGK
A0A1M6KV24	tr|A0A1M6KV24|A0A1M6KV24_9BACT	MEASQQNILLKIEGKGPVAEINFTVTLPEWLVEQVQSGSTVFLTQKEKMRFVLELARKNVAQETGGPFAAAVFSLESGELVSAGVNVVVESRCSSAHAEVVALSLAQKAVDSHDLGAAGLPRMVLVSSAEPCAMCMGAIPWSGVKQVICGARDEDVRSVGFDEGAKPLEWVEDFAERGIEVIRDVLREEATEVLWDYRERGGEIY
A0A2U0T9B4	tr|A0A2U0T9B4|A0A2U0T9B4_9RHIZ	METAELISRLLDVIEKDIAPVTAKGVARGNKLFGAAILKKSDLAVIVAETNNEIENPLWHGEMQAIKRFFELPADQRPATRDCLFLATHEPCSLCLSGITWSGFDNFYYLFSHQDSRDGFAIPYDIQILKSVYAVPEPETGTVSPARDLYNRSNDFWTSHGLQDMIAGLARSNREALLARIDDLNALYAELSERYQRDKGGKGIPLP
A0A2K9PN08	tr|A0A2K9PN08|A0A2K9PN08_9FLAO	MSDKKESKIKISKTSESIELDEIHSLLSYSIVQKFWENDDRNGRGYNVGVILVDENKNIVDWDINSVNKTENSTQHGEMRLISRYLDKDELYSLKGYTMYPTLEPCAMCAGMMTMTNVYRTVNGQMDYFYSKALERLSIDTRECGGYPPYPRTVISEISPSSISTRLDAEYKQYTNAGNKPIITKFLSTYKAKTIYDDAFNQFINFKCKFPENKTKYENAIKFYNSLPESI
F4PWM7	tr|F4PWM7|F4PWM7_CAVFA	MRFSLSLLFVILSVLLAGVLACKDPYNPETVDYGQCASATKANYEVRSDSKVLTPADLPADELAVHESRMRHIIDIARVNNKKFVSSIYFPNGTLACIGINTGKPNMIAHGEIVAIQNCTEIHGISMYTNYSIYTTGEPCSMCASAILWSRFKTVVWSTYNSDLYCKICMSNIPIDSSYIFSRAYGLGIEAPVAIGGVVKAEGDAWFGTYCNRPTSIYYIAPKCACQDPAKVSPLKFTQTRTTVWVEGGDKVVTQWNAIISNPSNSTIVDPPIVISPSVVFKGAPWGISAASEPNTYKLSYNKVLFPGQTFSFGYSVYGLEEVAFTALEA
U7QZM1	tr|U7QZM1|U7QZM1_PHOTE	MNKTRRKLLATLGIMSISMSFIAQAGEKKTQVINNILSKQEITEHEKYMREAIKEAIKNPKHPFGAVIVNRNNGEILSRGVNTGRNNPILHGEIQAINHYITQYGNQGWENVALYTTGEPCSMCMSALVWIGIREVIWATSISVIRNSGIRQIDISAHEIAERASSFYNPITLVGGILANETDKLFLERKRGN
A0A081CH48	tr|A0A081CH48|A0A081CH48_PSEA2	MASRRHLLATQVTGNHRKLSLWHLRGWLSPYTKLVDAVYFLTTNSFYHSLQTPPVQSITMLLSSIITSLALAAQASAYREGLHPEFQSGLSINSVPATDRDHWMRLANSAIYYPPVSHPCPQAPFGTAIVNTTSNELICAIANRVGSTGDPTQHGEITAIQHCTNVMRKKGLSPQEIIAAWKQLSLYTNAEPCTMCLSAIRWAGFKEVIYGTSVGTISENGRNQIYIPSNLVLEKSYSFGHATLMLGNILTHETDPFFQHQFNESAPCPVGCERTQVGEARVKTCEPVPNWQKLVRLEYSEDSRVGSEPVAHTPLHLEL
A0A3D3HMU1	tr|A0A3D3HMU1|A0A3D3HMU1_9GAMM	MDYSDAILGAITSIRRNSKQPGVNVTDNVTDSSTQYNNDEYWMRRALALAREAGEAGEIPVGAVLVKDNQQVAGGFNQPIRSHDPAAHAEILTLREAGAVLGNYRLIDTTLYVTLEPCMMCAGALVHSRIKRLVFGAAEPKTGAAGSFIDLLTLPRLNHYMEVTGGVLGEECSVLLSDFFRRRRAEKKALKRQNSESGSDSAS

<table>
<tr><td>A0A1N5WT13</td><td>tr|A0A1N5WT13|A0A1N5WT13_9ACTN</td><td>MLEKIERRLVAAAEAVVRSPSTGDAHTVAAAAMDANGDIYSGVNVFHFTGGPCAELVVIGSAAAANAPPLITIVAVGDGDRGVIAPCGRCRQVMLDLHPDVFVIVPTGDGQLAAKPVRELLPFGYVARTGSTAPRVVYFHPRHYDTISSGLKTATVRFQDSVQTGPAVFVFDDGESIRRLDAVVEKVESRRLDHLTEEDAHHEALPDSDALRDAIKTQYPMLGDGDVVDVATFRLTAISAPDPDPRSSYPPAVSRCNPAGPRADLLVGQS</td></tr>
<tr><td>X0SAC5</td><td>tr|X0SAC5|X0SAC5_9ZZZZ</td><td>MTKDGRVIASAHDTEVTDQDSTAHAEINAIRKASKIYRKDLTGCLIISTHEPCPMCTGSIIWSNISKVVYGVSIRDSIKAGRDMINLSCKEIIKKPNAEINIYDGILKKECLKLYNNDTRKLVKKFRKYEWINIEENLLNKRMQWFENNKTMIRKLKGNDLEKAYHLILMKIGIKRSEAPIVKKSESKIIFHSKNYCPSLEACIILDLDTREVCKEIYERPTEELIRRLNSKLRFTRNYDCIRPYSDYCEEIIILEK</td></tr>
<tr><td>A0A3B8IC10</td><td>tr|A0A3B8IC10|A0A3B8IC10_9BACT</td><td>MPSHEDFIHQCLELGKEALLQGNPPVGSVIVWQDQVIGRGIENGRSSGDITQHAELLALQEAVATGQRDKLKEAIIYSTHEPCVMCAYPIRQYKIPTVVYSVAVPELGGHTSSWHLLTTEDVPKWGKAPKIITGISAEEVEALNAAFQDSLKKG</td></tr>
<tr><td>A0A2N9P8B9</td><td>tr|A0A2N9P8B9|A0A2N9P8B9_9FLAO</td><td>MFIFKLISPPVSIEVYQDKIIQKLYICFMENIFTDEYFMKKALQEAETAFQQGEIPVGAVIVIDNRIIARSHNLTEMLNDVTAHAEMQAITASANFLGGKYLKDCTLYVTLEPCQMCAGALYWSQISKIVYGATDEQRGYRAMGAQLHPKTKVISGIMQNECTHLMKDFFKQRRSKSTKD</td></tr>
<tr><td>K1KX30</td><td>tr|K1KX30|K1KX30_9BACT</td><td>MVKNPVNNNELYFGKHSEIPMNEEQKAYMKMAVDLSRSGMESGKGGPFGCVIVKDGKVIGIGSNSVLETNDPTAHAEIVAIRDACRNLGHFQLDGCEVYTSCEPCPMCLGAIYWARPSKVFFANDKRDAAEAGFDDDFIYQELELPYEKRKIPFEQGMQDTAKEVFQEWILKEDKTLY</td></tr>
<tr><td>R4XI84</td><td>tr|R4XI84|R4XI84_TAPDE</td><td>MSSEIEPPSTDVHKHAVAEAADESGAADAFMQIALQQAETALLNKEVPVGCVFVHQPTGTVLATGANQTNASLNGTLHAEFVAIESILRDHPPSIFRESDLYVTVEPCVMCASALRQLQVRKVYFGCGNDRFGGCGSVFSIHSDASKTGDAAYMVESGIFRKEAIMLLRRFYLLQNESAPKPALKSTRVLKEHFDE</td></tr>
<tr><td>A0A239CVF7</td><td>tr|A0A239CVF7|A0A239CVF7_9DELT</td><td>MSPASKKHFPSLFSLFLLLTIGLICGTAHAQPQGHTADDTAATLANASLKEHEPFIRRCYQLAIDAGKKGNHPFGALLVHKGKIVLEAENTVLTDNDFTNHAEMNLIAEAARTLSRQIIPEATVYTSCAPCAMCTATLAMAGFTRIVYGVSHDALNKRFGLKGKSVSCPALFKTMGMELEFVGPVLEKEGLRVFDFWPEKDPHAQMLKKQARK</td></tr>
<tr><td>A0A1Q3NME1</td><td>tr|A0A1Q3NME1|A0A1Q3NME1_9BACT</td><td>MTEFNYDWAKLAFSSKRPLTNLKATFIIAPREISEKRFTQLLKEYLPKGDILLGISKEDYVEGLEGQPQFAMLQQKTLQKLIDKVNDASAHKVYTLRYFQRELPAIIEKLTPPRVVGIHGSWHHSFHTLPIYYLLSEKRIPYQLVAAFSDEDEARAYEVATDKKIVRPTLEGSFDDTTVLQLTDEVAKSSYDYGFQTGAILAEKVNGVYQPVAAGFNKVVPYQTYALLNGASRETNFSPANDMNHYDTIHAEMQILVEAAKQGISLKDKTLFVNLMPCPSCARTLSQTELSEIVYRIDHSGGYAVDLLTKVGKDIRRIVY</td></tr>
<tr><td>A0A2G6N4N7</td><td>tr|A0A2G6N4N7|A0A2G6N4N7_9DELT</td><td>MKERTVSYSDRHFMAEALEMAESALTQGEFPVGCVIADGTAVVARGHRTGTTAGAVNEIDHAEINALRHLGLAGEHLDRTDLTIYSTMEPCLMCFAAIVLSGINRIVYAYEDVMGGGTGCDLTGLPPLYRDAPLTLVAGVRRRASLNLFRRFFTDPENGYWAGSLLSRYTLNQTKDSHRL</td></tr>
<tr><td>A0A0G0RBB8</td><td>tr|A0A0G0RBB8|A0A0G0RBB8_9BACT</td><td>MQSVQYNKLTHLQRRALDEAEQVLENSYNPYSHFYVGACLISEDEQLIAGTNFENAAYGSAICAERAAVLRANAMSIRRFRGIAIIARGEDFNTTEVTGPCGSCRQVLYEISQVSGCDLQVILATSKKDKIVITTIRELLPLAFGPLDLGVDIGKY</td></tr>
<tr><td>A0A327L2Q5</td><td>tr|A0A327L2Q5|A0A327L2Q5_9RHIZ</td><td>MVTSRDGEDEAMMARCVALSRIAVGKGEYPFGAVVAREGRIVAEAINRTIRDGDVSRHAEVIALARAQKAIGRRELRECSLYSNVEPCAMCSYCIREAWVGRVVYALGSPVMGGVSKWNILRDDGLSGRMPQVFDAAPEVVSGVLVEQAQAAWRDWSPLAWEMITLRGLMTDPSARPECRTRAARPRSLWHHLVALIERPPRPYVDPTSAAEGHADL</td></tr>
<tr><td>S2DR30</td><td>tr|S2DR30|S2DR30_9BACT</td><td>MKMKKKIEITVSLEVIQKSEWSKEDRSLIERAIHAVEHAHAPYSNFMVGTALLLDNGQIFSANNQENVSFPVGICAERAVLSYAMGNFPNNRPVKLAVVAKRRSDSTWATVTPCGLCRQTINEYEVKFGHPIEILMLNPGEEILKASGIDQLLPFRFNDLNS</td></tr>
<tr><td>A0A369QGF1</td><td>tr|A0A369QGF1|A0A369QGF1_9BACT</td><td>MEEHEKWMHWCLNLAQQALQQGDFPVGAVVVQKGKLIGQGVEAGQLKKDITCHAEMEAIRDARQTINTADLQNCILYSTHEPCIMCSYVIRHHKISRVVVGTTVPEVGGSSSAYPLLSAPDISIWVAPPHLVTGVLAEACQALSQAYKQKFKK</td></tr>
<tr><td>A0A1W6X4U4</td><td>tr|A0A1W6X4U4|A0A1W6X4U4_9RHIZ</td><td>MTNPSRQERWDRRFLELAKVFGTWSKDRSAGTGCVIVGPDRLLRASGYNGFARGIDDEVPERHERPAKYSWTEHAERNAIYNAAKLGISLDGCTAYVNWFPCIDCARAIVQAGIVRLVGLHPDHADQRWGSEFKFATEMLRESGIEIILYDIPELAARK</td></tr>
<tr><td>A0A238BW09</td><td>tr|A0A238BW09|A0A238BW09_9BILA</td><td>MEEMARKIRTKAKKANSYCNTMTFLISKASIVLLKAECKRIELTVVIFRFLIKMNASEPNNELCDMTVIKSMLKITHVIFDLDGLLIDTEVVFSKVNQCLLSKYNKKFTPHLRGLVTGMPKKAAVTYILEHEKLSAKVDVDEYCKKYDEMAEEMLPKCSLMPGVMKLVRHLKTHSIPMAICTGATKKEFEIKTRYHKELLDLISLRVLSGDDPAVKRGKPAPDPFLVTMDRFKQKPEKAENVLVFEDAANGVCAAIAAGMNVIMVPDLTYMKIPEGLQNKINSFSDNLIISNDLNVALMSLKKELSEEEVHFLNRAFEIAVDAVLNNEVPVGCVFVFEGQEVAFGRNDVNRTKNPTYHAEMVALKMMKQWCMDNGRDLEEIMRRTTLYVTLEPCIMCASALYHLRLKKILYGAANERFGGLVSVGTREKYGAKHFIEIMPNLSVDRAVKLLKEFYEKQNPFCPEEKRKVKKPKKSGNNNDNSDDAVALNV</td></tr>
<tr><td>A0A1J5H6Z0</td><td>tr|A0A1J5H6Z0|A0A1J5H6Z0_9BACT</td><td>MAYQPSEKFMQMAIDKTREGVLSGQTPFGACIVKDGKVVACEHNTVWQDTDITSHGEVHTIRAACKAIGSIDLSGCILYSTCEPCPMCFSAIHWARIDTVVYGAFIADAQDAGFNELTISNEKMKEFGGSPVNFISGFMRDENVALFKLWKEQGANNVY</td></tr>
<tr><td>A0A3C2D945</td><td>tr|A0A3C2D945|A0A3C2D945_9BACT</td><td>MKTTEIRIIVHEYQNIDELTENDQYLLHEARRITEFAYAPYSGFHVGAAILLGNGMIVKGNNQENSAYPSGLCAERVALFYANANYPDSEVKTIAISAAKNGILVNDPIKPCGGCRQTLSEAEVRFGSPIRIILDGQDSILVLHGVESLLPLSFSKKDLASPLAATGR</td></tr>
<tr><td>A0A1I7EYS3</td><td>tr|A0A1I7EYS3|A0A1I7EYS3_9BURK</td><td>MKFKLDPSRPPDEDDYYLGVALAVRRKANCTGNRVAAVIVKNKRVIATGYNGVPEDMPNCLDGGCLRCSNPGGQFKSGTRYDLCICVHAEQNALLTTAARFGISVEGAHLYTTMQPCFGCAKEILQAKIEKVFYLHPWVPTDVDPVMDAAMKAEYAKIIGKLKVKKLDFDDPVATWAVTTMRQAALASDKNPDKKTPPKTAKKKVAKKKSRTSPR</td></tr>
<tr><td>H8GQX8</td><td>tr|H8GQX8|H8GQX8_METAL</td><td>MNHEHFMRRAIELARQAPQYPFGAVIVRRDDGQCVGQGFNRSDLNPTYHGEMVAINDCAVRHCAEDWRGFDLYTTAEPCAMCQGAIEWAGIGRVFYGTSIPYLQKLGWWQIDLRAAEVSARAVFRDTLIVGGILETECNALFAAARRGCFGTGSE</td></tr>
</table>

<table>
<tr><td>A0A0S8HZN3</td><td>tr|A0A0S8HZN3|A0A0S8HZN3_9CHLR</td><td>MDEHDIRFLRASFDVARNARKNGNHPFGALLVDEHGRIVMEAENTVITAKDCTGHAETNLMREASSKYDSDFLANCTIYTSTEPCPMCAGAIFWSNVRRVVYGLSEESLYEIAGRGSEEVLFLSCREIFERGKKLIEVIGPLLEDEAREVHMGFWR</td></tr>
<tr><td>E3SF31</td><td>tr|E3SF31|E3SF31_9CAUD</td><td>MKPTTVLQIAYLVSQESKCCSWKVGAVIEKNGRIISTGYNGSPAGGVNCCEHAEEQGWLLNKPKPVLIPGHKSECVRFSQVDRFVLAKAHREAHSAWSKNNEIHAELNAILFAARMGSSIEGATMYVTLSPCPDCAKAISQSGIKKLVYCETYDKNIPGWDDILKNAGIEVFNVPKRSLDKLNWENINEFCGE</td></tr>
<tr><td>F8AAC6</td><td>tr|F8AAC6|F8AAC6_THEID</td><td>MIRAPWHEYFMLLAKIVALRSGCNSRPSGAVIVKNKRILATGYNGPMPGAWHCTDRGPGYCFRREKGIPDIDKYNFCRATHAEANAIAQAARFGISVEGASLYCTLAPCYVCLKLIASAGIKKVYYEHDYGSRDFERDQFWKEAIKEAGLEKFEQITVSQEVMEQLQEILPYPTSKRRLAPTEFLDEFEDGKKYGVPSIEVLFNKLNYLTRQALKDITFVIEKTTVTEEPEGISFYLSGKMVELSELINTVKKQINADQNFYFLAKHNAIEAKIEILREAENIRLKAFLNECPLESFKRIAESLDYILYQVSNSLSLPTRLELSVNLLRI</td></tr>
<tr><td>A0A2H4ZNK4</td><td>tr|A0A2H4ZNK4|A0A2H4ZNK4_9EUKA</td><td>MKKQLSRKIQEEWMSRLLRNAYDAGTYGEVPIAAVILNESGQCIGWGRNCREKDQNPLGHAEIIALRQASYLKKSWRFNECTMLVTLEPCPMCAGALLQARINHIIYGASDYKRGGFGGVLDLSKNSSAHHKIEITRGVKSIQSCQLLETWFRRRRRV</td></tr>
<tr><td>A0A239N5N1</td><td>tr|A0A239N5N1|A0A239N5N1_9PSED</td><td>MEGRAGIIPFDEGGAAMGPAEEDSPMQHLAYMREALALARANVEAGGRPFGAVLVRDGEVIARAANGTHLDHDPTAHAELLALRAAGRALGSPRLDGCVVYASGHPCPMCLAAMHLSGVSAAYYAYSNADGEPYGLSTAAVYAQMAQPVEWQSLPLQALRPEDEEGLYGFWRERRP</td></tr>
<tr><td>A0A328VTR2</td><td>tr|A0A328VTR2|A0A328VTR2_9PSED</td><td>MHPEHLALLQQAPASTHADDTWARLCCEQALLAVEEGCYAVGALLVDGAGELLCSGRNQVFAPAYASAAHAEMRVLDQLEAEHAQVDRRSLTLYVSLEPCLMCYGRILLAGITRVRYLARDRDGGFALRHGRLPPAWANLASGLSVVQAKADPYWLDLAEHAIGRLQDRQTLRQRVIRAWRGQRTLTDEFSSTKRTHSG</td></tr>
<tr><td>A0A103YG48</td><td>tr|A0A103YG48|A0A103YG48_CYNCS</td><td>YIRELHASSLRRDEHEIQNPKILVIVDRLSSPSLHVSLSLSLSLVIFPPFIPLNQTPTHMENAKVVEAKDGTIAVASAFSGHQEVVQDRDHKFLTRAVEEAYKGVECGDGGPFGAVVVHKDEVVASCHNMVLKHTDPTAHAEVTAIREACKKLNKIELSDCEIYASCEPCPMCFGAIHLSRIKRLIYGAKAEAAIAIGFDDFIADALRGTGFYQKAHLEIKQADGNGAMIAEQVFEKTKAKFAIDHKFLTRAVEEAYKGVECGDGRPFGALVVHKDEVVVSCHNMVLNYTDPTAHAEITAIREACKKLNRIELSDCEMYSSCEPCPMCFGAIQISRIKRLVYGAKAEASIASGIPIGDFISDALKGTGFHEKANFEIKQADGNGAMIAEQVFERTKAMFPKR</td></tr>
<tr><td>W5M1M8</td><td>tr|W5M1M8|W5M1M8_LEPOC</td><td>NSSTRESRVMAQMEINGGASPPKKPGKGQSAADQDMITGLINKALQAKEFAYCPYSNFRVGAALMTNDGRVFTGCNVENACYNLGVCAERTAILKAVSEGYESFRAIAVSSDLQDQFISPCGACRQVMREFGTGWDVFLTKVDGSYVRMTVDELLPMSFGPDDLKKKKVFSLQNGHEVSTQFYTHSPCEAGENNN</td></tr>
<tr><td>A0A3N5YPZ2</td><td>tr|A0A3N5YPZ2|A0A3N5YPZ2_9ALTE</td><td>MSNSETEHIQALVDAAQAAQKQSYSPYSSFQVGAAIFADDGNTYSGCNIENVAYPLGQCAEATAIGMMIMQGAKRIEDIMIASPNDQVCPPCGGCRQKISEFGTAETKIHMVTRSGEVSTVTLGELLPLAFDSL</td></tr>
<tr><td>A0A2A9NC86</td><td>tr|A0A2A9NC86|A0A2A9NC86_9AGAR</td><td>MTNSTLSNEDRTRLIQGAFQARKKTYSPYSNFPVGAALLTTDGRIIEGANIENASYGGTICAERTAIVKAVSDGYRHFAGIAVTTKMPTRVSPCGICRQVLREFCSLDMPVLLVPGDYPQRNPVDDDGADKPGVITEGGVRETTLGALLPDSFGPENLPPRA</td></tr>
<tr><td>A0A2D6RD43</td><td>tr|A0A2D6RD43|A0A2D6RD43_9GAMM</td><td>MNIENLITENDETLIRRCIELAGESVKNGDKPFGALLAKDGNIIFESSNNAKTKVPYHAEILTLMDAQDKLNTTDLSDYALYSNCEPCPMCSFMIREYKLDKVVFSVHSPYMGGQSRWNILEDDVLTRFKPYFSKPPNVVGGVLESEGKRIFDKVGLWMFGKE</td></tr>
<tr><td>A0A0H3AVL6</td><td>tr|A0A0H3AVL6|A0A0H3AVL6_BRUO2</td><td>MHAKGYSQQERRIIPFANRFRFRELCSNKSLHGLRAKFPEQYTKWDPMRKAASITKANSATPMDIALEEAHAAGERGEVPIGAVIVRDGEIIARAGNRTREFNDVTAHAEILTIRQAGEMLGSERLIDCDLYVTLEPCAMCAAAISFARIRRLYYGASDPKGGGIEHGGRFYTQPTCHHAPEIYPGFCEADARKILKDFFREKR</td></tr>
<tr><td>A0A242H531</td><td>tr|A0A242H531|A0A242H531_9ENTE</td><td>MFIVKNNIEVIQQQAELDAKFMKQALKLAKDASNNGNEPFGAVLVKNDKVILTGENQIHTESDPTYHAELGIIRDFCTSQKITDLSEYTLYTSCEPCCMCAGAMVWSNLDRMVYGLGHDELAEIAGFNIMIGSEEIFSKSPNRPEVAKGVLKEAAVPVYVDYFQR</td></tr>
<tr><td>A0A2R6XZE2</td><td>tr|A0A2R6XZE2|A0A2R6XZE2_9BACL</td><td>MSGRISWHEYFMAQAKLIALRATCTRLMVGAVIVRDRRVIAGGYNGSIAGDEHCIDVGCKVRDGHCIRTIHAEQNALMQCAKFGVSTDGAELYVTHFPCLNCTKLLIQAGIRHIYYEVPYRVDPYAIELLEKAGVGTTQITVDLNAYVQVMSKVSTDPALTYVPESKAQKDEYGQSVGKIV</td></tr>
<tr><td>A0A139SHT6</td><td>tr|A0A139SHT6|A0A139SHT6_9BACT</td><td>MSEANASSESLPSRNSPVELIAEAAGKFGRRPTWDEYFMATAVLISTRSSCERLNVGCVIVTAGESHKNRIVAAGYNGHLPGSPHTSRMRDGHEQATVHAEQNAISDAARRGSSVEGCTAYVTHYPCINCAKILASAGIAKICYRLDYHNDPLVKPMLAEAGIEIVQLGEAAS</td></tr>
<tr><td>A0A261DBH2</td><td>tr|A0A261DBH2|A0A261DBH2_9RICK</td><td>MVMKKKLITVKRSTEFNNFFMEEALKQAQFALDKNEIPVGAIIVNRITNKVIAKAHNIVEQTKNPVLHAEIVAINQSCQILSSKNLSDCDMYVTLEPCVMCSGAISFARIGRLFYAANDPKQGAIENGGRFFNSKSCFYRPEIYSGFSAKISENLIKEFFYNVRYQKCNP</td></tr>
<tr><td>A0A2N0XZK6</td><td>tr|A0A2N0XZK6|A0A2N0XZK6_9VIBR</td><td>MTDNSLHESYMRQAFELSKSALPGCRPNPPVGCVFVKDGEVVSSGFSQPPGNHHAEAGAIAAYTGSYDGLVAYVTLEPCSFQGRTPSCAKALVRVRPEKVYVAILDPDTRNSGAGIKILEDAGIDVEVGLLGEEVASFLNPYLIRN</td></tr>
<tr><td>A0A1V5R0F9</td><td>tr|A0A1V5R0F9|A0A1V5R0F9_9BACT</td><td>MTKKETTKLHALDDFCMKKALLLAKRAFRADEVPVGALVVDSSNKVIGRGYNQVEKRKSQRAHAEQLAIEQACKKIGDWRLEGCTLYVTLEPCTMCMGLIKLSRIERVVFGAASPLFGYQLDKNRKSQLYKKGVIKIRKGVGKATAAALLKDFFKNKRM</td></tr>
<tr><td>A0A2W0H8Y3</td><td>tr|A0A2W0H8Y3|A0A2W0H8Y3_9BACI</td><td>MKNNGRLDHEYFMTEALQEAKEAGQRGDLPIGAVIVHNGRIIARGSNMRKTAGIKISHAENNAMHNCAPYLMKHASECVIYTTLEPCIMCLTTLVMANIDSIVFAADDKYMNMKPFIDANSYIRDRIHQYKGGVCRGESEALLRKYSPYAAELALNGTHPHHRKGGA</td></tr>
<tr><td>A0A261BDB7</td><td>tr|A0A261BDB7|A0A261BDB7_CAERE</td><td>LYKLYIFRMTTTKANLTQFEQELVDKAVGAMEKAYCKYSGFKVGAALVCEDGEIIIGANHENASYGATICAERSAMVTALTKGHRKFKLLAVATELEAPCSPCGICRQYLIEFGDYKVILGSSTSDQIIETTTYGLLPYAFTPKSLDDHEKEAEERNHQEGEKKH</td></tr>
</table>

A0A2E1PHI6	tr|A0A2E1PHI6|A0A2E1PHI6_9GAMM	MKELLIHSWLMLNSNSKLIMERVIELSEINLKNGKIPIAAVIVDKKNYEIISESQNEDSPIGHAELLAITKALKKLNTNRLDSTNLFVTIEPCPMCAYAISKCHINRLYFGSEDEKGGGVINGPRIFESHNLKKIDYVSHCYHEKTTQLMQSFFQLKRNQQL
A0A378LUA7	tr|A0A378LUA7|A0A378LUA7_9GAMM	MDTIIKKMISNAHNTLAHSYSPYSKFSVASCICTDKDNFYTGVNVENSAYGLAICAETSAISAMVTAGEKRIKSMVVMAGTNILCSPCGACRQRIYEFSTPDTLIHLCDKNSILRTFKINELLPEAFKFDFNP
A0A139HQ78	tr|A0A139HQ78|A0A139HQ78_9PEZI	MADSLKSKPGHARHDTALIHGLSQSDVQKLSESCVDAKSKAYCPYSHFRVGCAVLLANGDVVQGANVENAAYPVGTCAERVALGTAVGAKKGDFRALAVSTDISPPASPCGMCRQFIREFCELNTPILMYDKDGKSVVMTLEQLLPMSFGPDKLLPPGQLENGLMQTQTQSSFVTRAFSTTSSRRQDDTPQVPQSHYDFFPQTFPQGPPPKTSFSPDLKQLRKEFLQLQAKAHPDLAPQDQKRRAEALSMRINEAYKTLQSPLRRAQYLLSQQGIDVEDETAKLDDSSLLMEVMEAREAVEEVEDEEQLNEIRAENNGRIEESVRVLEDAFRDNEFEKAAQEAIRLRYWVNIEESIQGWEKGNGGGILHH
A0A2A9FXV0	tr|A0A2A9FXV0|A0A2A9FXV0_9VIBR	MCNLKENKDMDKYFHFACDATIEGMREGTGGPFGATLTRNGEVVCSVANTVLKDMDISGHAEMVAVREACKKLDTLDLSDCVMYATCEPCPMCVSVMLWAGIKTCYYASTHLDAAKHGFSDQQLRDYLDGSDTSTLNMVHIEDNRDDCAKIWTEFRHLNETKNDG
A0A1A8AG96	tr|A0A1A8AG96|A0A1A8AG96_NOTFU	MEHSDRWSRAEPGLSTSSRETRDGSTQTDCKLQGHGPRLSKVNLFTLLSLWMELFPQEQDEENGQSQIRRSGLVVVREGKVVGLHCSGADLHAGQAAILQHGASLANCQLFFSRRPCATCLKMIINAGVRQITFWPGDPEISMLTSNQTHSQRTSQSITEASLDATAVEKLKSNSRPQICVLMQPLAPGVLQFVDETSRRSDFMERMMDDDPELDSEKLFNSDRLRHLKDFCRHFLIQTDQRHKDILSQMGLKNFCVEPYFSNLRSNMTELVEVLAAVAAGMPQQHYGFYREESLSLDPHPVDVSQAVARHCIVQARLLSYRTEDPKVGVGAVIWAKGQSACCCGTGRLYLIGCGYNAYPAGSKYAEYPQMDNKQEDRERRKYRYIVHAEQNALTFRTRDIKPDECSMLFVTKCPCDECIPLIRGAGVKHIYTSDQDRDKDKGDISYLRFGSLKGVCKFIWQRSPPVSSASSLHLTNGCVGKHVRQAEQQIYKNKKLCTKGSSGSSDIC
A0A3E2VN88	tr|A0A3E2VN88|A0A3E2VN88_9FIRM	MEKEITNMDKQKLIQMAVDGLGRSYAPYSHFHVSAALLCADGTVYTGNNIENAAYTPSVCAERCAIFKAVGDGRREFEAIAVCGGPDGVIEDYCPPCGVCRQVMREFCDPSSFRVLVAKTAEDYREYTLEQLLPDGFGPDHLTGSGER
A0A2D5ZRJ2	tr|A0A2D5ZRJ2|A0A2D5ZRJ2_9BACT	MARPVHLHTGERRTEEGATESRAVAAVATAITRAPRAPPRPATGRERDGPPPRRVFGGGLRVGDPSGYDRGESKPIGGPLTEKRSDWHSYFMRIAGEVATRATCDRKHVGAVIVRNRTILSTGYNGSIRGMPHCDDVGHDMVDGHCIATIHAEANAILQAARNGVMIQDGSIYITASPCWNCFKLVANAGLKRVYYGEFYRDKRSFEVARRLGIDLMHIEV
A0A1B8WPS3	tr|A0A1B8WPS3|A0A1B8WPS3_9BACI	MEGVQLIYQFQWGNLIMTVNKEDLYLIDVARNTIKTLYVDGKHHVGAAVRTKTGKIYSAVHLEANIGRVSVCAEAIALGKAISEGESEFDTIVAVRHPDPTQENQKIEVVSPCGICRELISDYGKGTNVILKNKEGYIKTVISDLLPNKYIREDN
A0A1W5ZQK9	tr|A0A1W5ZQK9|A0A1W5ZQK9_9BACI	MNRFMERAVSLAAENVRVGGQPFGAVLVKDDELVAEGVNEMHLNYDVSGHAELLAIRRAQGELQTHDLSGYTMYASGEPCPMCLSAMYFAGIKDVFYCATVEEAAQVGLEKSKNVYDDLQKSKGERSLVMKQMPLEDDQEDPMKLWDERTNHNGTS
A0A378V0W4	tr|A0A378V0W4|A0A378V0W4_MYCFO	MVHAQFDPTARQALAATAVEAKTRKDLTWQQIADAAELSPAFVTAAVLGQHALPARSAEAVAALLGLDDDAALLLQTIPIRGSIPGGIPTDPTIYRFYEMLQVYGTTLKALVHEQFGDGIISAINFKLDVRKVADPEGGERAVITLDGKYLPPNPFDRVRYRGGLMDFAQRTIDIARQNVAEGGRPFATVIVKNGEILAESPNLVAQTHDPTAHAEILAIRKACTRIGTEHLIGATIYVLAQPCPMCLGSLYYCSPDEVVFLTTRDAYEPHYVDDRKYFELNMFYDEFAKPWDQRRLPMRYEPRDAAVDVYKLWQERNGGERRVPGAPTSTRPGKNPRGE
I3XF03	tr|I3XF03|I3XF03_RHIFR	MKQRCMSPKSAQRFWDNDMHNNKDRPMSENELFVAAREAMAKAHAPYSKFPVGAAIRAEDGQIYTGANIENLSFPEGWCAETTAISHMVMAGQRKIMEVAVIAEKLALCPPCGGCRQRLAEFSGASTRIYLCDETGIKKSLALSDLLPHSFETEILG
F8IEF3	tr|F8IEF3|F8IEF3_ALIAT	MDAKELETRGWLCMRAVDVIDKKRRGEALAEEELRFLIEGYVAGRIPDYQMSAFLMAVVWRGMTREETLVLTRLLADSGERLDLSGIPGVKVDKHSTGGVGDKATLVVLPLVASIGVPVIKMSGRGLGHTGGTIDKLESIPGFRTDLSVAELVAQVRQVGIALGGQTADLAPADKKLYALRDVTGTVESLPLIASSVMSKKLAGGADAIVLDVKVGDGAFMKSRSDARRLARLMVEIGEAAGRRTVAVLSNMDQPLGCAIGNALEVAEAIRVLSGEGPFDLAEIALALAEEMTVLAGVAATREEARRMLRQSVAEGRALETLRRWIAAQGGDPAVVDDPSRLPQAPVQMPYLPKKAGFVAKLSALAFGLAAMRLGAGRETKEEAIDPSVGIVLHAKVGDRVQTHRPMFTVHARTGEDALRCIQELEAAIQISDDPVEAPPLILARIDRSEALPYADLMDAAREARDRAYVPYSGFAVGAALELADGRMVTGANVENASYGLTNCAERSAVFRAVAEGGPGTKPEIRAVAVIADSPEPVSPCGACRQVLAEFCSPDTPVYLGNLQGDVRETTVGALLPGAFTDAQMANVRRQDKEA
A0A1G3M638	tr|A0A1G3M638|A0A1G3M638_9SPIR	MKTTNINALDKWDLRFLQMAEHVAEWSKDPSTKVGAVIVRPDRTIASVGFNGFARGVRDTVERLWNRELKYPLTVHAELNAILSAHEPVRGHSLYVSPLSPCSNCAGVIIQSGIARVVAKCGQVNNPAQWSESFNLALTAFAEAGVSVILVEH
A0A3D9LFR2	tr|A0A3D9LFR2|A0A3D9LFR2_9MICC	MEQNDHGSSGAFSDPFEDDIPLTASLPRITGTGSGIDWQRLESTARAAMTRAYVPYSRFPVGAAALVEDGRVVAGCNIENASLGLTLCAECSLVSNLQMSGGGRIVAFYCVDGNGEVLMPCGRCRQLLYEFHAPGMRLMGPDGELTMDEVLPLAFGPADMTHLSDSAASTDDPGRTR
A0A3B9YGB5	tr|A0A3B9YGB5|A0A3B9YGB5_9BACT	MAKPISKKYRKLIETAKAARKKAYSPYSRYQVGAAVLTESGRIYSGANMENASYGLCMCAERVAIANAVTRGEKVLQAVCVVGKKARPCGACRQVMLEFSTKETELLMVDIDPNARRDTVIRTRVYSMLPNPFDPFESGMLPQHPQNLLRRRKSPQPRRKRRSRPVHREVSR
A0A182F569	tr|A0A182F569|A0A182F569_ANOAL	MPRPSQFRVSSSQSLSNSQIQASQSSDSVVDITSYVNAVVKALLNLSCTKTIIKRADLVNIALKGNGRLIGRVLQDANIELKEIYGYELIEVEKSKTMILCSTLAAGSMDELNDANRRRYTFLYLILGYIFMKNGSVPETIVWEFLETLGIEEQQEHNYFGDVRKLYDSLFKQAYLTRTKQALEGLNDDVMLISWGVRSKHEVSKKDILAGFCKVMNRDPVDFKAQYIEANEKDDKMNNNINGTVDGRNTVEYSSLDASVKELIEAAIKVRNNAYCPYSNFAVGAALRTVGGDIVTGCNVENGTFGPSVCAERTAVCKAVSEGHREFTAVAVVAFQETEFTAPCGTCRQTLSEFSRKDIPIYLVKPSPVRVMVTSLFQLLPHAFSPSFLNK
A0A264Z0D4	tr|A0A264Z0D4|A0A264Z0D4_9BACI	MEPKKLIEEAIVASKQAYVQYSNFHVGAALLTKDGKLYHGCNIENASYGLTNCAERTAIFKAVSEGEKEFQAIAVVGDTEGPISPCGACRQVLAEFFSPDTVVILANLKGDHVVTNINELLPGFFSSKDLQKKVKNCFEKNALGSSCLRPI

A0A1L9Q1R3	tr|A0A1L9Q1R3|A0A1L9Q1R3_ASPVE	MPLSAEEAALVETATATINSIPLSEDYSVASAAKASDGRVFTGVNVYHFTGGPCAELVVLGVAAAAGAAQLTHIVAVANEQRGILSPCGRCRQVLLDLQPNIQVIVGKEGSEQSVPVAQLLPFSYRQPDQHTPVIFKALTSSGPVVVDFFATWCGPCKAVAPVVGKLSETYTDVRFIQVDVDKARSISQEHDIRAMPTFVLYKDGKLLDKRVVGGNMKELEEQIKAIIA

Supplementary Table 2. Sequences of sgRNAs used in this study. Target sites for guided off-target[2], targeted RNA-seq[3] and Figure 5b[4] are the same as previous publication and not listed.

S. pyogenes SgRNA scaffold*:*
GUUUUAGAGCUAGAAAUAGCAAGUUAAAAUAAGGCUAGUCCGUUAUCAACUUGAAAAAGUGGCACCGAGUCGGUGC

S. aureus SgRNA scaffold*:*
GUUUUAGUACUCUGUAAUGAAAAUUACAGAAUCUACUAAAACAAGGCAAAAUGCCGUGUUUAUCUCGUCAACUUGUUGGCGAGA

site	protospacer sequence	PAM	Cas9 scaffold	PAM	Cas9 scaffold
1	GAUGUGUCUACUGUUACUUACA	AGGAAT	S. aureus	AGG	S. pyogenes
2	GCACCCAGGGGUUCUGCAGAGC	AGGGAT	S. aureus	AGG	S. pyogenes
3	GCAUUCCACUCCGUCCGCCUC	CGGAGT	S. aureus	CGG	S. pyogenes
4	GCCACAGACUUUUCCAUUUGC	AGGAGT	S. aureus	AGG	S. pyogenes
5	GCCACAGUGGGAGGGGACAUG	GGGAAT	S. aureus	GGG	S. pyogenes
6	GCCCAGCAAUUCACUGUGAAG	AGGGAT	S. aureus	AGG	S. pyogenes
7	GCCCAGCUCCAGCCUCUGAUG	AGGGGT	S. aureus	AGG	S. pyogenes
8	GCCCUGAUCUGCACUGAACAG	AGGGGT	S. aureus	AGG	S. pyogenes
9	GCCUCAAGUCUGGUUAUUUUAG	GGGGAT	S. aureus	GGG	S. pyogenes
10	GCCUGGCAGAUGAGAACCAGG	AGGAAT	S. aureus	AGG	S. pyogenes
11	GCGAAAGGCUCGCGGCGAAGGA	AGGAAT	S. aureus	AGG	S. pyogenes
12	GCUCCUCUCACCCUUAUGACUC	AGGGAT	S. aureus	AGG	S. pyogenes
13	GCUGCAAGGGUUGGCCAGGCU	GGGAAT	S. aureus	GGG	S. pyogenes
14	GGCCUCCGUAUCACUCUCUGAC	TGGGGT	S. aureus	TGG	S. pyogenes
15	GGGUACCUGAGUGGGGUGCAUU	TGGGGT	S. aureus	TGG	S. pyogenes
16	GGUCGACCCUUGGUAUCCAUG	GGGGAT	S. aureus	GGG	S. pyogenes
17	GGUCGUAGCCAGUCCGAACCC	CGGAGT	S. aureus	CGG	S. pyogenes
18	GUAACUGAACCCCUGCAAUCAA	TGGGAT	S. aureus	TGG	S. pyogenes
19	GGCCUCCGUAUCACUCUCUGAC	TGGGGT	S. aureus	TGG	S. pyogenes
20	GCUUUCCUUAGCUGUAAAAGAA	AGGGAT	S. aureus	AGG	S. pyogenes
21	GUGGCACUGCGGCUGGAGGU	GGGGGT	S. aureus	GGG	S. pyogenes
22	GUAGGGCCUUCGCGCACCUCA	TGGAAT	S. aureus	TGG	S. pyogenes
23	GGCCUCCCCAAAGCCUGGCCA	GGGAGT	S. aureus	GGG	S. pyogenes
24	GCACAUUCACGGUCUCAGUGC	AAGGAT	S. aureus	AAG	S. pyogenes
25	GGAAACCUUGAAUAAGAAUGGA	AGGGGT	S. aureus	AGG	S. pyogenes
26	GUAUUACUAUUAUUAUCUGAGA	TGGGGT	S. aureus	TGG	S. pyogenes
27	GUGGGACUGAUCCCUUAAUGUG	TGGGGT	S. aureus	TGG	S. pyogenes
28	GAAAGAGACAGAGAAGGGGCA	GGGGGT	S. aureus	GGG	S. pyogenes
29	GAAGGCUUUACUGUAUUACAGA	AGGGGT	S. aureus	AGG	S. pyogenes
30	GACCAAAACGAGGGACAUUUA	GGGGAT	S. aureus	GGG	S. pyogenes
31	GACCAGGUCAGCAAACAUGUU	TGGAAT	S. aureus	TGG	S. pyogenes
32	GACUCAGCGCCCCUGCCGGGCC	TGGGAT	S. aureus	TGG	S. pyogenes
33	GAGAAGAAACCAGGGAACAGGU	AGGAGT	S. aureus	AGG	S. pyogenes
34	GAGUGGGAACUUUCUGAUGCCA	TGGAAT	S. aureus	TGG	S. pyogenes

Supplementary Table 3. DNA sequences of oligos used in this study. Primers for guided off-target[2] and targeted RNA-seq[3] are the same as previous publication and not listed.

Oligos used *in vitro* assays (adaptor sequences were highlighted in yellow, * stands for phosphorothioate bonds):

Oligo1:
T*G*GTTTGTGTATTGGGTGAAGGTGAAAGGGTGAAAAAAATTG**TCT**GTAAGTAAGGGTGGTAAAGAA
TAAATGTAGGAATTTTGGTGGG*A*T
Oligo2:
G*G*TTTGTGTATTGGGTGCCTTCTATTTCCAGCTCGAAGCGAAAAAACAGATAAGTTCATAACCGCAT
GTAGGAATTTTGGTGGGA*T*A
Oligo3:
G*G*TTTGTGTATTGGGTGTATCTTAACAATGTTAATAACGTATAAAGGCTGTTCATTCCCTCGCGCAT
GTAGGAATTTTGGTGGGA*T*A
Oligo4:
G*G*TTTGTGTATTGGGTGCGTCAACTTTTCCAGTTCTTAATTAAGACCTTTAGCCGTGTGTGTAAATG
TAGGAATTTTGGTGGGA*T*A
Oligo5:
G*G*TTTGTGTATTGGGTGGTTTAGCGATTTAACTTCTTAAAAAAAAACGTCAGCCCTAAGGTATTATG
TAGGAATTTTGGTGGGA*T*A

Primers used in amplicon sequencing:

HTS_FP_site1	ACACTCTTTCCCTACACGACGCTCTTCCGATCTACTGTCTTTTGATCTACAGCAGTTAAT
HTS_FP_site2	ACACTCTTTCCCTACACGACGCTCTTCCGATCTAGCCTCTTTCCTGCTAGAGC
HTS_FP_site3	ACACTCTTTCCCTACACGACGCTCTTCCGATCTCTTTCGCTGCCCTTTCCTCT
HTS_FP_site4	ACACTCTTTCCCTACACGACGCTCTTCCGATCTGATATCTCCAGGCTCCTGTCCATTCT
HTS_FP_site5	ACACTCTTTCCCTACACGACGCTCTTCCGATCTCCATCCTAAGTGAAGCAGCATATTTGA
HTS_FP_site6	ACACTCTTTCCCTACACGACGCTCTTCCGATCTAGGTGGGGGTGACTCCTTTTTTGGA
HTS_FP_site7	ACACTCTTTCCCTACACGACGCTCTTCCGATCTCTTGTCTGTCCAAGGAGAATGAGGTC
HTS_FP_site8	ACACTCTTTCCCTACACGACGCTCTTCCGATCTGACCTGGAGGCCTGGGATCCACA
HTS_FP_site9	ACACTCTTTCCCTACACGACGCTCTTCCGATCTCCTTTAGGACACATGCTGTCTACCACA
HTS_FP_site10	ACACTCTTTCCCTACACGACGCTCTTCCGATCTGCCAAAGTCTGAGGTTTAGTTGACTAA
HTS_FP_site11	ACACTCTTTCCCTACACGACGCTCTTCCGATCTAAGCGGGTTCTCATTGTTCCCGTGTCT
HTS_FP_site12	ACACTCTTTCCCTACACGACGCTCTTCCGATCTAACCAGTCCCTGTCCTGAATCTATCTA
HTS_FP_site13	ACACTCTTTCCCTACACGACGCTCTTCCGATCTTTGCTTTCGGGTATCTACTAGGAGTCA
HTS_FP_site14	ACACTCTTTCCCTACACGACGCTCTTCCGATCTGGGCTATCAAACCTCATGATTGGC
HTS_FP_site15	ACACTCTTTCCCTACACGACGCTCTTCCGATCTAAGCTGTCCAGCTGGAAGCCTGGTAA
HTS_FP_site16	ACACTCTTTCCCTACACGACGCTCTTCCGATCTGCCTAAGTTATATGCAAACATCATGCC
HTS_FP_site17	ACACTCTTTCCCTACACGACGCTCTTCCGATCTGCTGCTGGAATACCGAGGAC
HTS_FP_site18	ACACTCTTTCCCTACACGACGCTCTTCCGATCTACGAGGTAAGTGTGTGGATTAGTTTCA

HTS_FP_site19	ACACTCTTTCCCTACACGACGCTCTTCCGATCTAGTGGTTACTTTGCCGGGTT
HTS_FP_site20	ACACTCTTTCCCTACACGACGCTCTTCCGATCTACCCTAGCCCTCGGTGCCCTTAGTT
HTS_FP_site21	ACACTCTTTCCCTACACGACGCTCTTCCGATCTNNNNGAACCCAGGTAGCCAGAGAC
HTS_FP_site22	ACACTCTTTCCCTACACGACGCTCTTCCGATCTNNNNCATTGCAGAGAGGCGTATCA
HTS_FP_site23	ACACTCTTTCCCTACACGACGCTCTTCCGATCTNNNNCAGAGTGCTGCTTGCTGCT
HTS_FP_site24	ACACTCTTTCCCTACACGACGCTCTTCCGATCTNNNNGAAACCATGTCTCTGGATGCC
HTS_FP_site25	ACACTCTTTCCCTACACGACGCTCTTCCGATCTNNNNAGGCCTTTTCTTGGGGATGC
HTS_FP_site26	ACACTCTTTCCCTACACGACGCTCTTCCGATCTGTGGGAACATCACCGGAGCCTGG
HTS_FP_site27	ACACTCTTTCCCTACACGACGCTCTTCCGATCTCTGACACTAAATATGTGGTTTTTGCT
HTS_FP_site28	ACACTCTTTCCCTACACGACGCTCTTCCGATCTCGAACTCCTAGGCTCAAGTAATCCA
HTS_FP_site29	ACACTCTTTCCCTACACGACGCTCTTCCGATCTGCCAGTAATTGCATTAAACCCTCACTA
HTS_FP_site30	ACACTCTTTCCCTACACGACGCTCTTCCGATCTGGCTCCCACTCTCTCCCAGTGTCCTCA
HTS_FP_site31	ACACTCTTTCCCTACACGACGCTCTTCCGATCTTCTGCCTGTGTGAAGCTCCC
HTS_FP_site32	ACACTCTTTCCCTACACGACGCTCTTCCGATCTGGGAGTCCTCCCTTCACCCCTGC
HTS_FP_site33	ACACTCTTTCCCTACACGACGCTCTTCCGATCTGTGCCAAGGCATAAAAGCCTTCCCTG
HTS_FP_site34	ACACTCTTTCCCTACACGACGCTCTTCCGATCTACTCGCTGGCCTGGCCTTTCTTCTC
HTS_RP_site1	TGGAGTTCAGACGTGTGCTCTTCCGATCTAAGAAACAGATTACAGAAGTAGATGCA
HTS_RP_site2	TGGAGTTCAGACGTGTGCTCTTCCGATCTTCTCTCCTATGTGCTGGCCT
HTS_RP_site3	TGGAGTTCAGACGTGTGCTCTTCCGATCTCTACACTGGAACCCCGACTC
HTS_RP_site4	TGGAGTTCAGACGTGTGCTCTTCCGATCTCCAGCCGATATTTCAGAACTAATCAGA
HTS_RP_site5	TGGAGTTCAGACGTGTGCTCTTCCGATCTAACAATGGCAAGGGCCTGCCCTG
HTS_RP_site6	TGGAGTTCAGACGTGTGCTCTTCCGATCTGGGCAGAAGGAAAAATCTATCCTGGAA
HTS_RP_site7	TGGAGTTCAGACGTGTGCTCTTCCGATCTGCACAGAACCCGCTGCTAGAGACTCCA
HTS_RP_site8	TGGAGTTCAGACGTGTGCTCTTCCGATCTGGAAAGTCTGGTTAGAGCTCAGAGGGA
HTS_RP_site9	TGGAGTTCAGACGTGTGCTCTTCCGATCTGTGGTGGAGTGCTCTGTGTTTGTCT
HTS_RP_site10	TGGAGTTCAGACGTGTGCTCTTCCGATCTATTACAGGTGTGGGCCACCTTGCCC
HTS_RP_site11	TGGAGTTCAGACGTGTGCTCTTCCGATCTTCCGCCTCCGGAGTAGGGCTGCAGAGA
HTS_RP_site12	TGGAGTTCAGACGTGTGCTCTTCCGATCTGGAAGGCAGACTGTATCTGGTCTTTT
HTS_RP_site13	TGGAGTTCAGACGTGTGCTCTTCCGATCTTCTAGCAGGAAAGAGGCTCAGGCCCA
HTS_RP_site14	TGGAGTTCAGACGTGTGCTCTTCCGATCTACACACAGACACTGCAGAGAATAACA
HTS_RP_site15	TGGAGTTCAGACGTGTGCTCTTCCGATCTCCGCCCAGCACTCGCAGAGCAGA
HTS_RP_site16	TGGAGTTCAGACGTGTGCTCTTCCGATCTGATGAGAATGCACCATGATTCCAATCA
HTS_RP_site17	TGGAGTTCAGACGTGTGCTCTTCCGATCTGCAACTCTCTTTTCTCCGGGA
HTS_RP_site18	TGGAGTTCAGACGTGTGCTCTTCCGATCTCTACCAAGGAGAGTCATTCCTTTCAGA
HTS_RP_site19	TGGAGTTCAGACGTGTGCTCTTCCGATCTAAGACAGTCTGGGAAGCGTG
HTS_RP_site20	TGGAGTTCAGACGTGTGCTCTTCCGATCTACTTGGTATTTTAGAAGACTGTTTTCT
HTS_RP_site21	TGGAGTTCAGACGTGTGCTCTTCCGATCTTCCTTTCAACCCGAACGGAG
HTS_RP_site22	TGGAGTTCAGACGTGTGCTCTTCCGATCTGGGGTCCCAGGTGCTGAC
HTS_RP_site23	TGGAGTTCAGACGTGTGCTCTTCCGATCTAAAAGGGAGATTGGAGACACGGAGA

HTS_RP_site24	TGGAGTTCAGACGTGTGCTCTTCCGATCTAGAGAAATCACACTAGCTAGCCT
HTS_RP_site25	TGGAGTTCAGACGTGTGCTCTTCCGATCTTGCTCTCAACAAAAGTGAAGAACA
HTS_RP_site26	TGGAGTTCAGACGTGTGCTCTTCCGATCTTGCATAACCTACACACATCCTCTGATA
HTS_RP_site27	TGGAGTTCAGACGTGTGCTCTTCCGATCTGGATTGCGGAAATCCCCAACTTATAGC
HTS_RP_site28	TGGAGTTCAGACGTGTGCTCTTCCGATCTGCCTGGACTCCAGACAGGCTTCC
HTS_RP_site29	TGGAGTTCAGACGTGTGCTCTTCCGATCTAAGGCCAAGAATCTTGCTAGTAGTGGA
HTS_RP_site30	TGGAGTTCAGACGTGTGCTCTTCCGATCTGGATAGAGCAAAAGAAGTAGTGCCTGG
HTS_RP_site31	TGGAGTTCAGACGTGTGCTCTTCCGATCTTGAAACTGTCACTGAAACATCTGGT
HTS_RP_site32	TGGAGTTCAGACGTGTGCTCTTCCGATCTGTTCTCAAGAAAAGGCCACCCCTCAG
HTS_RP_site33	TGGAGTTCAGACGTGTGCTCTTCCGATCTTGCTTAGAGGGTAAAAACCCAGGAGGA
HTS_RP_site34	TGGAGTTCAGACGTGTGCTCTTCCGATCTGGGAGAGAGGCAGGGCGGGCATG

Supplementary Table 4. DNA sequences of mammalian expression plasmids for the core CBEs showed in this study. The deaminase sequence is highlighted for BE4-rAPOBEC1. For the rest of constructs, only the deaminase sequences are shown. The backbone sequences are identical.

BE4-rAPOBEC1
TGCTTCGCGATGTACGGGCCAGATATACGCGTTGACATTGATTATTGACTAGTTATTAATAGTAATCAATTACGGGGTCATTAGT
TCATAGCCCATATATGGAGTTCCGCGTTACATAACTTACGGTAAATGGCCCGCCTGGCTGACCGCCCAACGACCCCCGCCCAT
TGACGTCAATAATGACGTATGTTCCCATAGTAACGCCAATAGGGACTTTCCATTGACGTCAATGGGTGGAGTATTTACGGTAAAC
TGCCCACTTGGCAGTACATCAAGTGTATCATATGCCAAGTACGCCCCCTATTGACGTCAATGACGGTAAATGGCCCGCCTGGCA
TTATGCCCAGTACATGACCTTATGGGACTTTCCTACTTGGCAGTACATCTACGTATTAGTCATCGCTATTACCATGGTGATGCGG
TTTTGGCAGTACATCAATGGGCGTGGATAGCGGTTTGACTCACGGGGATTTCCAAGTCTCCACCCCATTGACGTCAATGGGAGT
TTGTTTTGGCACCAAAATCAACGGGACTTTCCAAAATGTCGTAACAACTCCGCCCCATTGACGCAAATGGGCGGTAGGCGTGTA
CGGTGGGAGGTCTATATAAGCAGAGCTGGTTTAGTGAACCGTCAGATCCGCTAGAGATCCGCGGCCGCTAATACGACTCACTA
TAGGGAGAGCCGCCACCATGAGCAGCGAGACAGGCCCTGTGGCCGTGGACCCCACCCTGCGGCGGAGAATCGAGCCTCATG
AGTTCGAGGTGTTCTTCGACCCTCGGGAACTGAGAAAAGAGACATGCCTGCTGTACGAGATCAACTGGGGCGGAAGACACAGC
ATCTGGCGGCACACCAGCCAGAACACCAACAAGCACGTGGAAGTGAATTTCATCGAGAAGTTCACCACCGAAAGATACTTCTG
CCCCAACACCAGATGCAGCATCACATGGTTCCTGTCTTGGTCCCCTTGCGGCGAGTGCTCTAGAGCCATCACCGAGTTCCTGA
GCAGATATCCTCACGTGACACTGTTCATCTACATCGCCAGACTGTATCACCACGCCGATCCTAGAAATAGACAGGGCCTGCGG
GACCTGATCAGCTCCGGCGTGACCATCCAGATCATGACCGAGCAGGAGAGCGGCTACTGTTGGAGAAACTTCGTGAACTACTC
TCCTAGCAACGAGGCCCACTGGCCTAGATACCCCCACCTGTGGGTGCGGCTGTACGTGCTGGAACTGTACTGCATCATCCTGG
GACTGCCTCCATGTCTGAACATCCTGAGAAGAAAGCAGCCTCAGCTGACCTTCTTCACAATCGCCCTGCAGAGCTGCCACTAC
CAGAGACTGCCCCCCCACATCCTGTGGGCCACCGGCCTGAAGCTTAAGAGCGGAGGATCTCTTAAGAGCGGAGGATCTAGCG
GCGGCTCTAGCGGATCTGAGACACCTGGCACAAGCGAGTCTGCCACACCTGAGAGTAGCGGCGGATCTTCTGGTGGCTCTGA
CAAGAAGTACAGCATCGGCCTGGCCATCGGCACCAACTCTGTGGGCTGGGCCGTGATCACCGACGAGTACAAGGTGCCCAGC
AAGAAATTCAAGGTGCTGGGCAACACCGACCGGCACAGCATCAAGAAGAACCTGATCGGAGCCCTGCTGTTCGACAGCGGCG
AAACAGCCGAGGCCACCCGGCTGAAGAGAACCGCCAGAAGAAGATACACCAGACGGAAGAACCGGATCTGCTATCTGCAAGA
GATCTTCAGCAACGAGATGGCCAAGGTGGACGACAGCTTCTTCCACAGACTGGAAGAGTCCTTCCTGGTGGAAGAGGATAAGA
AGCACGAGCGGCACCCCATCTTCGGCAACATCGTGGACGAGGTGGCCTACCACGAGAAGTACCCCACCATCTACCACCTGAG
AAAGAAACTGGTGGACAGCACCGACAAGGCCGACCTGCGGCTGATCTATCTGGCCCTGGCCCACATGATCAAGTTCCGGGGC
CACTTCCTGATCGAGGGCGACCTGAACCCCGACAACAGCGACGTGGACAAGCTGTTCATCCAGCTGGTGCAGACCTACAACCA
GCTGTTCGAGGAAAACCCCATCAACGCCAGCGGCGTGGACGCCAAGGCCATCCTGTCTGCCAGACTGAGCAAGAGCAGACGG
CTGGAAAATCTGATCGCCCAGCTGCCCGGCGAGAAGAAGAATGGCCTGTTCGGAAACCTGATTGCCCTGAGCCTGGGCCTGA
CCCCCAACTTCAAGAGCAACTTCGACCTGGCCGAGGATGCCAAACTGCAGCTGAGCAAGGACACCTACGACGACGACCTGGA
CAACCTGCTGGCCCAGATCGGCGACCAGTACGCCGACCTGTTTCTGGCCGCCAAGAACCTGTCCGACGCCATCCTGCTGAGC
GACATCCTGAGAGTGAACACCGAGATCACCAAGGCCCCCCTGAGCGCCTCTATGATCAAGAGATACGACGAGCACCACCAGG
ACCTGACCCTGCTGAAAGCTCTCGTGCGGCAGCAGCTGCCTGAGAAGTACAAAGAGATTTTCTTCGACCAGAGCAAGAACGGC
TACGCCGGCTACATTGACGGCGGAGCCAGCCAGGAAGAGTTCTACAAGTTCATCAAGCCCATCCTGGAAAAGATGGACGGCAC
CGAGGAACTGCTCGTGAAGCTGAACAGAGAGGACCTGCTGCGGAAGCAGCGGACCTTCGACAACGGCAGCATCCCCCACCAG
ATCCACCTGGGAGAGCTGCACGCCATTCTGCGGCGGCAGGAAGATTTTTACCCATTCCTGAAGGACAACCGGGAAAAGATCGA
GAAGATCCTGACCTTCCGCATCCCCTACTACGTGGGCCCTCTGGCCAGGGGAAACAGCAGATTCGCCTGGATGACCAGAAAGA
GCGAGGAAACCATCACCCCCTGGAACTTCGAGGAAGTGGTGGACAAGGGCGCTTCCGCCCAGAGCTTCATCGAGCGGATGAC
CAACTTCGATAAGAACCTGCCCAACGAGAAGGTGCTGCCCAAGCACAGCCTGCTGTACGAGTACTTCACCGTGTATAACGAGC
TGACCAAAGTGAAATACGTGACCGAGGGAATGAGAAAGCCCGCCTTCCTGAGCGGCGAGCAGAAAAAGGCCATCGTGGACCT
GCTGTTCAAGACCAACCGGAAAGTGACCGTGAAGCAGCTGAAAGAGGACTACTTCAAGAAAATCGAGTGCTTCGACTCCGTGG
AAATCTCCGGCGTGGAAGATCGGTTCAACGCCTCCCTGGGCACATACCACGATCTGCTGAAAATTATCAAGGACAAGGACTTC
CTGGACAATGAGGAAAACGAGGACATTCTGGAAGATATCGTGCTGACCCTGACACTGTTTGAGGACAGAGAGATGATCGAGGA
ACGGCTGAAAACCTATGCCCACCTGTTCGACGACAAAGTGATGAAGCAGCTGAAGCGGCGGAGATACACCGGCTGGGGCAGG
CTGAGCCGGAAGCTGATCAACGGCATCCGGGACAAGCAGTCCGGCAAGACAATCCTGGATTTCCTGAAGTCCGACGGCTTCG
CCAACAGAAACTTCATGCAGCTGATCCACGACGACAGCCTGACCTTTAAAGAGGACATCCAGAAAGCCCAGGTGTCCGGCCAG
GGCGATAGCCTGCACGAGCACATTGCCAATCTGGCCGGCAGCCCCGCCATTAAGAAGGGCATCCTGCAGACAGTGAAGGTGG
TGGACGAGCTCGTGAAAGTGATGGGCCGGCACAAGCCCGAGAACATCGTGATCGAAATGGCCAGAGAGAACCAGACCACCCA
GAAGGGACAGAAGAACAGCCGCGAGAGAATGAAGCGGATCGAAGAGGGCATCAAAGAGCTGGGCAGCCAGATCCTGAAAGAA
CACCCCGTGGAAAACACCCAGCTGCAGAACGAGAAGCTGTACCTGTACTACCTGCAGAATGGGCGGGATATGTACGTGGACCA
GGAACTGGACATCAACCGGCTGTCCGACTACGATGTGGACCATATCGTGCCTCAGAGCTTTCTGAAGGACGACTCCATCGACA
ACAAGGTGCTGACCAGAAGCGACAAGAACCGGGGCAAGAGCGACAACGTGCCCTCCGAAGAGGTCGTGAAGAAGATGAAGAA
CTACTGGCGGCAGCTGCTGAACGCCAAGCTGATTACCCAGAGAAAGTTCGACAATCTGACCAAGGCCGAGAGAGGCGGCCTG
AGCGAACTGGATAAGGCCGGCTTCATCAAGAGACAGCTGGTGGAAACCCGGCAGATCACAAAGCACGTGGCACAGATCCTGG
ACTCCCGGATGAACACTAAGTACGACGAGAATGACAAGCTGATCCGGGAAGTGAAAGTGATCACCCTGAAGTCCAAGCTGGTG
TCCGATTTCCGGAAGGATTTCCAGTTTTACAAAGTGCGCGAGATCAACAACTACCACCACGCCCACGACGCCTACCTGAACGC
CGTCGTGGGAACCGCCCTGATCAAAAAGTACCCTAAGCTGGAAAGCGAGTTCGTGTACGGCGACTACAAGGTGTACGACGTGC
GGAAGATGATCGCCAAGAGCGAGCAGGAAATCGGCAAGGCTACCGCCAAGTACTTCTTCTACAGCAACATCATGAACTTTTTCA
AGACCGAGATTACCCTGGCCAACGGCGAGATCCGGAAGCGGCCTCTGATCGAGACAAACGGCGAAACCGGGGAGATCGTGTG

GGATAAGGGCCGGGATTTTGCCACCGTGCGGAAAGTGCTGAGCATGCCCCAAGTGAATATCGTGAAAAAGACCGAGGTGCAG
ACAGGCGGCTTCAGCAAAGAGTCTATCCTGCCCAAGAGGAACAGCGATAAGCTGATCGCCAGAAAGAAGGACTGGGACCCTAA
GAAGTACGGCGGCTTCGACAGCCCCACCGTGGCCTATTCTGTGCTGGTGGTGGCCAAAGTGGAAAAGGGCAAGTCCAAGAAA
CTGAAGAGTGTGAAAGAGCTGCTGGGGATCACCATCATGGAAAGAAGCAGCTTCGAGAAGAATCCCATCGACTTTCTGGAAGC
CAAGGGCTACAAAGAAGTGAAAAAGGACCTGATCATCAAGCTGCCTAAGTACTCCCTGTTCGAGCTGGAAAACGGCCGGAAGA
GAATGCTGGCCTCTGCCGGCGAACTGCAGAAGGGAAACGAACTGGCCCTGCCCTCCAAATATGTGAACTTCCTGTACCTGGCC
AGCCACTATGAGAAGCTGAAGGGCTCCCCCGAGGATAATGAGCAGAAACAGCTGTTTGTGGAACAGCACAAGCACTACCTGGA
CGAGATCATCGAGCAGATCAGCGAGTTCTCCAAGAGAGTGATCCTGGCCGACGCTAATCTGGACAAAGTGCTGTCCGCCTACA
ACAAGCACCGGGATAAGCCCATCAGAGAGCAGGCCGAGAATATCATCCACCTGTTTACCCTGACCAATCTGGGAGCCCCTGCC
GCCTTCAAGTACTTTGACACCACCATCGACCGGAAGAGGTACACCAGCACCAAAGAGGTGCTGGACGCCACCCTGATCCACCA
GAGCATCACCGGCCTGTACGAGACACGGATCGACCTGTCTCAGCTGGGAGGTGACTCTGGTGGAAGCGGAGGATCTGGCGG
CAGCACCAATCTGAGCGACATCATCGAGAAAGAGACAGGCAAGCAGCTGGTCATCCAAGAGTCCATCCTGATGCTGCCTGAAG
AGGTGGAAGAAGTGATCGGCAACAAGCCCGAGTCCGACATCCTGGTGCACACCGCCTACGATGAGAGCACCGACGAGAACGT
GATGCTGCTGACCTCTGACGCCCCTGAGTACAAGCCTTGGGCTCTCGTGATCCAGGACAGCAACGGCGAGAACAAGATCAAGA
TGCTGAGCGGCGGCTCTGGTGGCTCTGGCGGATCTACAAACCTGTCCGATATTATTGAGAAAGAAACCGGGAAACAGCTCGTG
ATTCAAGAGTCTATTCTCATGCTCCCGGAAGAAGTCGAGGAAGTCATTGGAAACAAGCCTGAGAGCGATATTCTGGTCCATACA
GCCTACGACGAGTCTACCGATGAGAATGTCATGCTCCTCACCAGCGACGCTCCCGAGTATAAGCCATGGGCACTTGTCATTCA
GGACTCCAATGGGGAAAACAAAATCAAAATGCTCCCAAAGAAAAAACGCAAGGTGGAGGGAGCTGATAAGCGCACCGCCGATG
GTTCCGAGTTCGAAAGCCCCAAGAAGAAGAGGAAAGTCTAACCGGTCATCATCACCATCACCATTGAGTTTAAACCCGCTGATC
AGCCTCGACTGTGCCTTCTAGTTGCCAGCCATCTGTTGTTTGCCCCTCCCCCGTGCCTTCCTTGACCCTGGAAGGTGCCACTCC
CACTGTCCTTTCCTAATAAAATGAGGAAATTGCATCGCATTGTCTGAGTAGGTGTCATTCTATTCTGGGGGGTGGGGTGGGGCA
GGACAGCAAGGGGGAGGATTGGGAAGACAATAGCAGGCATGCTGGGGATGCGGTGGGCTCTATGGCTTCTGAGGCGGAAAG
AACCAGCTGGGGCTCGATACCGTCGACCTCTAGCTAGAGCTTGGCGTAATCATGGTCATAGCTGTTTCCTGTGTGAAATTGTTA
TCCGCTCACAATTCCACACAACATACGAGCCGGAAGCATAAAGTGTAAAGCCTAGGGTGCCTAATGAGTGAGCTAACTCACATT
AATTGCGTTGCGCTCACTGCCCGCTTTCCAGTCGGGAAACCTGTCGTGCCAGCTGCATTAATGAATCGGCCAACGCGCGGGGA
GAGGCGGTTTGCGTATTGGGCGCTCTTCCGCTTCCTCGCTCACTGACTCGCTGCGCTCGGTCGTTCGGCTGCGGCGAGCGGT
ATCAGCTCACTCAAAGGCGGTAATACGGTTATCCACAGAATCAGGGGATAACGCAGGAAAGAACATGTGAGCAAAAGGCCAGC
AAAAGGCCAGGAACCGTAAAAAGGCCGCGTTGCTGGCGTTTTTCCATAGGCTCCGCCCCCCTGACGAGCATCACAAAAATCGA
CGCTCAAGTCAGAGGTGGCGAAACCCGACAGGACTATAAAGATACCAGGCGTTTCCCCCTGGAAGCTCCCTCGTGCGCTCTCC
TGTTCCGACCCTGCCGCTTACCGGATACCTGTCCGCCTTTCTCCCTTCGGGAAGCGTGGCGCTTTCTCATAGCTCACGCTGTA
GGTATCTCAGTTCGGTGTAGGTCGTTCGCTCCAAGCTGGGCTGTGTGCACGAACCCCCCGTTCAGCCCGACCGCTGCGCCTTA
TCCGGTAACTATCGTCTTGAGTCCAACCCGGTAAGACACGACTTATCGCCACTGGCAGCAGCCACTGGTAACAGGATTAGCAG
AGCGAGGTATGTAGGCGGTGCTACAGAGTTCTTGAAGTGGTGGCCTAACTACGGCTACACTAGAAGAACAGTATTTGGTATCTG
CGCTCTGCTGAAGCCAGTTACCTTCGGAAAAAGAGTTGGTAGCTCTTGATCCGGCAAACAAACCACCGCTGGTAGCGGTGGTT
TTTTTGTTTGCAAGCAGCAGATTACGCGCAGAAAAAAAGGATCTCAAGAAGATCCTTTGATCTTTTCTACGGGGTCTGACGCTCA
GTGGAACGAAAACTCACGTTAAGGGATTTTGGTCATGAGATTATCAAAAAGGATCTTCACCTAGATCCTTTTAAATTAAAAATGAA
GTTTTAAATCAATCTAAAGTATATATGAGTAAACTTGGTCTGACAGTTACCAATGCTTAATCAGTGAGGCACCTATCTCAGCGATC
TGTCTATTTCGTTCATCCATAGTTGCCTGACTCCCCGTCGTGTAGATAACTACGATACGGGAGGGCTTACCATCTGGCCCCAGT
GCTGCAATGATACCGCGAGACCCACGCTCACCGGCTCCAGATTTATCAGCAATAAACCAGCCAGCCGGAAGGGCCGAGCGCA
GAAGTGGTCCTGCAACTTTATCCGCCTCCATCCAGTCTATTAATTGTTGCCGGGAAGCTAGAGTAAGTAGTTCGCCAGTTAATA
GTTTGCGCAACGTTGTTGCCATTGCTACAGGCATCGTGGTGTCACGCTCGTCGTTTGGTATGGCTTCATTCAGCTCCGGTTCCC
AACGATCAAGGCGAGTTACATGATCCCCCATGTTGTGCAAAAAAGCGGTTAGCTCCTTCGGTCCTCCGATCGTTGTCAGAAGTA
AGTTGGCCGCAGTGTTATCACTCATGGTTATGGCAGCACTGCATAATTCTCTTACTGTCATGCCATCCGTAAGATGCTTTTCTGT
GACTGGTGAGTACTCAACCAAGTCATTCTGAGAATAGTGTATGCGGCGACCGAGTTGCTCTTGCCCGGCGTCAATACGGGATA
ATACCGCGCCACATAGCAGAACTTTAAAAGTGCTCATCATTGGAAAACGTTCTTCGGGGCGAAAACTCTCAAGGATCTTACCGC
TGTTGAGATCCAGTTCGATGTAACCCACTCGTGCACCCAACTGATCTTCAGCATCTTTTACTTTCACCAGCGTTTCTGGGTGAGC
AAAAACAGGAAGGCAAAATGCCGCAAAAAAGGGAATAAGGGCGACACGGAAATGTTGAATACTCATACTCTTCCTTTTTCAATAT
TATTGAAGCATTTATCAGGGTTATTGTCTCATGAGCGGATACATATTTGAATGTATTTAGAAAAATAAACAAATAGGGGTTCCGCG
CACATTTCCCCGAAAAGTGCCACCTGACGTCGACGGATCGGGAGATCGATCTCCCGATCCCCTAGGGTCTTACTCTCAGTACA
ATCTGCTCTGATGCCGCATAGTTAAGCCAGTATCTGCTCCCTGCTTGTGTGTTGGAGGTCGCTGAGTAGTGCGCGAGCAAAATT
TAAGCTACAACAAGGCAAGGCTTGACCGACAATTGCATGAAGAATCTGCTTAGGGTTAGGCGTTTTGCGC

BE4-PpAPOBEC1

ATGACCTCTGAGAAGGGCCCTAGCACAGGCGACCCCACCCTGCGGCGGAGAATCGAGAGCTGGGAGTTCGACGTGTTCTACG
ACCCTAGAGAACTGAGAAAGGAAACCTGCCTGCTGTACGAGATCAAGTGGGGCATGAGCAGAAAGATCTGGCGGAGCTCTGG
CAAGAACACCACCAACCACGTGGAAGTGAATTTCATCAAGAAGTTCACCAGCGAGAGAAGGTTCCACAGCAGCATCAGCTGCA
GCATCACCTGGTTCCTGAGCTGGTCCCCTTGCTGGGAATGCAGCCAGGCCATCAGAGAGTTCCTGAGCCAACACCCCGGAGT
GACACTGGTGATCTACGTGGCCAGACTGTTCTGGCACATGGACCAGAGAAACAGACAGGGCCTGAGAGATCTGGTCAACAGC
GGCGTGACTATCCAGATCATGCGGGCCAGCGAGTACTACCACTGTTGGCGGAACTTCGTGAACTACCCCCCCGGCGATGAGG
CCCACTGGCCTCAGTACCCTCCTCTGTGGATGATGCTGTACGCCCTGGAACTGCACTGCATCATCCTGTCTCTGCCTCCATGTC
TGAAGATCTCTAGAAGATGGCAGAACCACCTGGCCTTCTTCAGACTGCACCTGCAGAATTGCCACTACCAGACCATCCCCCCC
CACATCCTGCTGGCTACAGGCCTGATCCACCCTTCTGTGACCTGGAGA

BE4-RrA3F

ATGAAGCCCCAGATCAGGGACCACCGCCCCAATCCTATGGAGGCCATGTACCCTCACATCTTCTATTTTCACTTCGAGAACCTG
GAGAAGGCCTACGGCCGGAATGAGACCTGGCTGTGCTTTACAGTGGAGATCATCAAGCAGTATCTGCCAGTGCCCTGGAAGAA

GGGCGTGTTCCGGAACCAGGTGGATCCAGAGACCCACTGCCACGCCGAGAAGTGTTTTCTGTCCTGGTTCTGTAACAATACAC
TGTCTCCCAAGAAGAATTACCAGGTGACCTGGTATACAAGCTGGTCCCCTTGCCCAGAGTGTGCAGGAGAGGTGGCAGAGTTT
CTGGCAGAGCACAGCAACGTGAAGCTGACCATCTACACAGCCCGGCTGTACTATTTCTGGGACACCGATTATCAGGAGGGCCT
GAGATCTCTGAGCGAGGAGGGCGCCTCCGTGGAGATCATGGACTACGAGGATTTTCAGTATTGCTGGGAGAACTTCGTGTACG
ACGATGGCGAGCCTTTTAAGAGGTGGAAGGGCCTGAAGTATAATTTCCAGTCTCTGACACGGAGACTGCGCGAGATCCTGCAG

BE4-AmAPOBEC1

ATGGCCGACAGCTCCGAGAAGATGAGGGGCCAGTACATCAGCCGCGACACCTTTGAGAAGAATTATAAGCCCATCGATGGCAC
AAAGGAGGCCCACCTGCTGTGCGAGATCAAGTGGGGCAAGTACGGCAAGCCTTGGCTGCACTGGTGTCAGAATCAGCGGATG
AACATCCACGCCGAGGACTATTTCATGAACAATATCTTTAAGGCCAAGAAGCACCCTGTGCACTGCTACGTGACCTGGTATCTG
TCTTGGAGCCCATGCGCCGATTGTGCCTCCAAGATCGTGAAGTTCCTGGAGGAGCGGCCCTACCTGAAGCTGACCATCTATGT
GGCCCAGCTGTACTATCACACAGAGGAGGAGAATAGGAAGGGCCTGCGGCTGCTGCGGAGCAAGAAAGTGATCATCCGCGTG
ATGGACATCTCCGATTACAACTATTGCTGGAAGGTGTTCGTGTCTAACCAGAATGGCAACGAGGACTACTGGCCACTGCAGTTT
GATCCCTGGGTGAAGGAGAATTATTCTCGGCTGCTGGATATCTTCTGGGAGTCCAAGTGTAGATCTCCCAACCCTTGG

BE4-SsAPOBEC3B

ATGGACCCACAGAGGCTGCGCCAGTGGCCCGGCCCTGGCCCAGCAAGCAGGGGCGGCTACGGCCAGCGGCCAAGAATCAG
GAACCCCGAGGAGTGGTTTCACGAGCTGTCTCCCCGGACCTTCAGCTTTCACTTCCGCAACCTGAGGTTCGCATCCGGCCGCA
ATCGGTCTTATATCTGCTGTCAGGTGGAGGGCAAGAACTGCTTCTTTCAGGGCATCTTTCAGAATCAGGTGCCACCTGACCCAC
CATGCCACGCAGAGCTGTGCTTCCTGTCTTGGTTCCAGAGCTGGGGCCTGTCCCCCGATGAGCACTACTATGTGACATGGTTT
ATCTCTTGGAGCCCTTGCTGTGAGTGTGCCGCCAAGGTGGCCCAGTTCCTGGAGGAGAACCGCAACGTGAGCCTGTCTCTGA
GCGCCGCAAGGCTGTACTATTTCTGGAAGTCCGAGTCTAGAGAGGGACTGCGGAGACTGAGCGACCTGGGAGCACAAGTGGG
AATCATGTCCTTTCAGGATTTCCAGCACTGCTGGAACAATTTTGTGCACAACCTGGGCATGCCCTTCCAGCCTTGGAAGAAGCT
GCACAAGAATTACCAGAGGCTGGTGACCGAGCTGAAGCAGATCCTGCGCGAGGAGCCTGCCACATATGGCTCTCCACAGGCC
CAGGGCAAGGTGAGAATCGGAAGCACCGCAGCAGGACTGAGGCACAGCCACTCCCACACACGCTCCGAGGCACACCTGAGG
CCTAACCACAGCTCCAGACAGCACAGGATCCTGAATCCTCCACGGGAGGCCAGAGCCAGGACCTGCGTGCTGGTGGATGCCT
CTTGGATCTGTTACAGA

Supplementary Note 1: Methods of creating similarity network of cytidine deaminases. To focus the search space within the APOBEC1-like protein family, human APOBEC1 was used as a query sequence for a protein BLAST search against the NCBI non-redundant protein sequences database (nr_v5). The top 1000 sequences were used to generate a sequence similarity network (SSN) with a protein BLAST -log(E-value) edge-threshold of 115. A set of 43 deaminases was selected to sample the sequence space within the SSN.

To identify deaminases from other families that could act as base-editing enzymes, we sampled 80 sequences from a SSN built from all deaminases with the following InterPro annotations IPR002125 (Cytidine and deoxycytidylate deaminase domain), IPR016192 (APOBEC/CMP deaminase, zinc-binding), and IPR016193 (Cytidine deaminase-like). This set of 82,043 sequences was first clustered at 55% identity using Cd-HIT[5] before generating a SSN network by protein BLAST with a -log(E-value) edge-threshold of 50. Sequences were chosen based on their centrality within a cluster of sequence in the network.

Supplementary Note 2: Discussion about protein expression level of base editors.

We would like to examine if different protein expression level of editors contributes to changes in *cis/trans* editing profile. Quantification of base editor protein was performed on cells transfected with editor plasmids, we showed that HiFi mutations like K34A and H122A didn't cause significant changes in base editor transcription and translation. For all 4 new CBEs we characterized in this study, the protein expression level was not dramatically lower than BE4-rAPOBEC1 (**Supplementary Figure 14)**. As a result, we believe that changes in *cis/trans* editing profile came from the intrinsic characteristics of deaminases.

Supplementary Note 3: Targeted NGS analysis details

1. To generate FASTQ files from the base call files (BCF) generated by the MiSeq, demultiplexing was performed by running Illumina bcl2fastq (v2.20.0.422) with the following parameters:

```
bcl2fastq \
        --ignore-missing-bcls \
        --ignore-missing-filter \
        --ignore-missing-positions \
        --ignore-missing-controls \
        --auto-set-to-zero-barcode-mismatches \
        --find-adapters-with-sliding-window \
        --adapter-stringency 0.9 \
        --mask-short-adapter-reads 35 \
        --minimum-trimmed-read-length 35 \
```

2. The FASTQ files created in step (1) were processed using trimmomatic (v0.39)[6] with parameters set up to clip Illumina TruSeq adapters, exclude reads shorter than 20 bases, and trim the remaining 3' end of reads if the average base quality (Phred score) in a 4-bp sliding window dropped below 15. In addition, any bases with quality scores of 3 or lower at the end of reads were removed. Finally, because the round 1 PCR primers include four randomized bases after the read 1 primer sequence, the first four bases of each read were trimmed. The command used to execute trimmomatic is shown below:

```
trimmomatic SE -phred33 $input_fastq $output_fastq \
        ILLUMINACLIP:illumine_adapters.fa:2:30:10 \
        LEADING:3 TRAILING:3 \
        SLIDINGWINDOW:4:15 \
        MINLEN:20 \
        HEADCROP:4
```

3. Reads were aligned to amplicon sequences using bowtie2 (v2.35)[7], in end-to-end mode with the alignment parameters specified by the --very sensitive flag. Reference sequences were determined as the expected amplicon sequences (including primers) for each primer pair based on the human genome (GRCh38).The SAM files created by bowtie2 were converted to BAM files, sorted, and indexed using the samtools package (v1.9)[8]. Only samples with at least 5,000 aligned reads were considered for analysis.

4. The BAM files created in step (3) were processed using the bam-readcounts tool (https://github.com/genome/bam-readcount) to generate plain text files summarizing the number of non-reference bases, deletions and insertions at each position in the alignment. The minimum base quality (Phred score) for counting a non-reference base was set to 29 in order to exclude low confidence base calls from statistics about editing rates. Only reads with insertions and/or deletions that overlapped the base editor target site (defined as its protospacer + PAM sequence) were counted towards insertion and deletion rates. Editing rates for each position in the target site were calculated as the fraction of non-reference bases of a given type (e.g., G) to the total number of bases passing the base quality threshold at a given position in the alignment.

Supplementary Note 4: Transcriptome sequencing analysis method.

FASTQ files were downloaded from Novagene and aligned to the human genome (Gencode GRCh38v31) using STAR (v2.7.2a). Genome alignments were then duplicate marked and sorted with Picard (v2.20.5). Reads that contain Ns in their cigar string because they span splicing junctions were split using GATK (v4.1.3.0) and then base quality score recalibration was performed with Picard. Variant calls were generated with GATK Haplotype Caller with standard settings for variant calling in RNA: minimum-mapping-quality 30, minimum-base-quality 20, dont-use-soft-clipped-bases, standard-call-conf 20.

To identify somatic mutations private to our base-editor treated samples, background filtration was performed using an nCas9 treated sample. Only substitutions on canonical chromosomes were considered. A mutation was determined to be private to the base-editor treated sample if its genomic position had ≥30x coverage in the base-editor treated sample and ≥20x coverage in the nCas9 sample with 99% of reads containing the reference base.

Supplementary Note 5: Whole genome NGS analysis details.

1. Lane level FASTQ files were separately aligned to the human genome (Gencode GRCh38v31 primary assembly) using BWA (0.7.17-r1188) mem with parameters set to specify the ReadGroup. The -M flag was also set to mark shorted split hits a secondary alignment.

2. Lane level genome alignments for each sample created in step (1) were merged, sorted by coordinate, and duplicate marked using Picard (v2.20.5) using default settings. GATK (V4.1.3.0) was used to do base quality score recalibration.

3. Variants were called using GATK (v4.1.3.0) HaplotypeCaller. Only reads with a mapping quality ≥ 30 were considered and the minimum base quality (Phred score) for counting a non-reference base was set to 20. Standard settings for variant calling in DNA-seq were used.

4. Mutations private to base-editor treated samples were identified using background filtration. The highest coverage ‘No Treatment’ sample was used as the background sample. Only substitutions on canonical chromosomes were considered. Mutations were considered private to the base-editor treated sample if they met the following criteria:

a. The genomic position of the mutation had coverage ≥ 10 reads in the treated and untreated sample.
b. The untreated sample had ≥ 99% of reads supporting the reference, non-mutant, base at the position of the mutation.

References:

1. McGrath, E. et al. Targeting specificity of APOBEC-based cytosine base editor in human iPSCs determined by whole genome sequencing. *Nat Commun* **10**, 5353 (2019).
2. Tsai, S.Q. et al. GUIDE-seq enables genome-wide profiling of off-target cleavage by CRISPR-Cas nucleases. *Nat Biotechnol* **33**, 187-197 (2015).
3. Rees, H.A., Wilson, C., Doman, J.L. & Liu, D.R. Analysis and minimization of cellular RNA editing by DNA adenine base editors. *Sci Adv* **5**, eaax5717 (2019).
4. Doman, J.L., Raguram, A., Newby, G.A. & Liu, D.R. Evaluation and minimization of Cas9-independent off-target DNA editing by cytosine base editors. *Nat Biotechnol* (2020).
5. Li, W. & Godzik, A. Cd-hit: a fast program for clustering and comparing large sets of protein or nucleotide sequences. *Bioinformatics* **22**, 1658-1659 (2006).
6. Bolger, A.M., Lohse, M. & Usadel, B. Trimmomatic: a flexible trimmer for Illumina sequence data. *Bioinformatics* **30**, 2114-2120 (2014).
7. Langmead, B. & Salzberg, S.L. Fast gapped-read alignment with Bowtie 2. *Nat Methods* **9**, 357-359 (2012).
8. Li, H. et al. The Sequence Alignment/Map format and SAMtools. *Bioinformatics* **25**, 2078-2079 (2009).
