# Supplementary material for: Cytosine base editors with minimized unguided DNA and RNA off-target events and high on-target activity
Source: Nat Commun. 2020 Apr 28;11:2052. doi: 10.1038/s41467-020-15887-5 (PMC7189382; doi:10.1038/s41467-020-15887-5)
Supplement: Supplementary file 2 — Reporting Summary [file 41467_2020_15887_MOESM2_ESM.pdf]

nature research

Corresponding author(s): Nicole M. Gaudelli, Giuseppe Ciaramella

Last updated by author(s): 3-18-2020

Reporting Summary

Nature Research wishes to improve the reproducibility of the work that we publish. This form provides structure for consistency and transparency in reporting. For further information on Nature Research policies, see Authors & Referees and the Editorial Policy Checklist .

Statistics

For all statistical analyses, confirm that the following items are present in the figure legend, table legend, main text, or Methods section.

n/a	Confirmed	
☐	☒	The exact sample size (*n*) for each experimental group/condition, given as a discrete number and unit of measurement
☐	☒	A statement on whether measurements were taken from distinct samples or whether the same sample was measured repeatedly
☐	☒	The statistical test(s) used AND whether they are one- or two-sided<br>*Only common tests should be described solely by name; describe more complex techniques in the Methods section.*
☒	☐	A description of all covariates tested
☒	☐	A description of any assumptions or corrections, such as tests of normality and adjustment for multiple comparisons
☐	☒	A full description of the statistical parameters including central tendency (e.g. means) or other basic estimates (e.g. regression coefficient) AND variation (e.g. standard deviation) or associated estimates of uncertainty (e.g. confidence intervals)
☐	☒	For null hypothesis testing, the test statistic (e.g. *F*, *t*, *r*) with confidence intervals, effect sizes, degrees of freedom and *P* value noted<br>*Give P values as exact values whenever suitable.*
☒	☐	For Bayesian analysis, information on the choice of priors and Markov chain Monte Carlo settings
☒	☐	For hierarchical and complex designs, identification of the appropriate level for tests and full reporting of outcomes
☒	☐	Estimates of effect sizes (e.g. Cohen's *d*, Pearson's *r*), indicating how they were calculated

Our web collection on statistics for biologists contains articles on many of the points above.

Software and code

Policy information about availability of computer code

Data collection	Illumina MiSeq Control Software (v3.1)
Data analysis	Illumina bcl2fastq (v2.20.0.422), trimmomatic (v0.39), bowtie2 (v2.35), samtools (v1.9), bam-readcounts (v0.8), R (v3.4.3), Excel (v16.32), Prism8 (v 8.3.0), STAR (v2.7.2a), Picard (v2.20.5), GATK (v4.1.3.0), BWA (0.7.17-r1188)

For manuscripts utilizing custom algorithms or software that are central to the research but not yet described in published literature, software must be made available to editors/reviewers. We strongly encourage code deposition in a community repository (e.g. GitHub). See the Nature Research guidelines for submitting code & software for further information.

Data

Policy information about availability of data

All manuscripts must include a data availability statement. This statement should provide the following information, where applicable:
- Accession codes, unique identifiers, or web links for publicly available datasets
- A list of figures that have associated raw data
- A description of any restrictions on data availability

Next-generation sequencing data underlying all experiments are deposited in the NCBI Sequence Read Archive (PRJNA595157).

Field-specific reporting

Please select the one below that is the best fit for your research. If you are not sure, read the appropriate sections before making your selection.

☒ Life sciences ☐ Behavioural & social sciences ☐ Ecological, evolutionary & environmental sciences

For a reference copy of the document with all sections, see nature.com/documents/nr-reporting-summary-flat.pdf

Life sciences study design

All studies must disclose on these points even when the disclosure is negative.

Sample size	No sample size calculations were performed in advance.
Data exclusions	NGS samples withe the less than 5000 reads were excluded from the study.
Replication	All measurements reported in the study were collected in at least two independent biological replicates.
Randomization	Randomization was not considered, as all major experimental variables were fully under the control of the experimenters.
Blinding	Blinding was not considered, as all data analysis was performed following uniform automated processes.

Reporting for specific materials, systems and methods

We require information from authors about some types of materials, experimental systems and methods used in many studies. Here, indicate whether each material, system or method listed is relevant to your study. If you are not sure if a list item applies to your research, read the appropriate section before selecting a response.

Materials & experimental systems

n/a	Involved in the study	
☐	☒	Antibodies
☐	☒	Eukaryotic cell lines
☒	☐	Palaeontology
☒	☐	Animals and other organisms
☒	☐	Human research participants
☒	☐	Clinical data

Methods

n/a	Involved in the study	
☒	☐	ChIP-seq
☒	☐	Flow cytometry
☒	☐	MRI-based neuroimaging

Antibodies

Antibodies used	CRISPR-Cas9 Antibody (7A9-3A3) (Novus Biologicials, NBP2-36440); B2M antibody 2M2 (Biolegend, 316306, Lot 13262121)
Validation	CRISCas9 antibody: https://www.novusbio.com/products/crispr-cas9-antibody-7a9-3a3_nbp2-36440#reviews-publications<br>Product insert shows staining of HEK293t with Cas9 antibody versus Hela WT control<br><br>B2M: https://www.biolegend.com/en-us/products/pe-anti-human-beta2-microglobulin-antibody-3080<br>Product insert shows staining of human lymphocytes with B2M-PE versus isotype control

Eukaryotic cell lines

Policy information about cell lines

Cell line source(s)	Hek293T: American Type Cell Culture Collection (ATCC)
Authentication	Done by supplier.
Mycoplasma contamination	Tested negative for mycoplasma contamination by the supplier, ATCC.
Commonly misidentified lines (See ICLAC register)	The cell line used is not listed as commonly misidentified.
